# The effect of antenatal psychological well-being on maternal health status

**DOI:** 10.1371/journal.pone.0336684

**Published:** 2025-11-14

**Authors:** Mays Shudayfat, Esra’ O. Taybeh, Mervat Alsous

**Affiliations:** 1 Department of Applied Pharmaceutical Sciences and Clinical Pharmacy, Isra University, Amman, Jordan; 2 Department of Biopharmaceutics and Clinical Pharmacy, The University of Jordan, Amman, Jordan; Korea University - Seoul Campus: Korea University, KOREA, REPUBLIC OF

## Abstract

**Background:**

The presence of psychological pressures may negatively affect the health of pregnant women pre-, during, and post-childbirth and may lead to many complications.

**Aim:**

The aim of the present study was to assess the psychological well-being among pregnant women in Jordan and to explore the relationship between psychological pressures and gestational health consequences.

**Method:**

A cross-sectional study was performed with pregnant women at 24–28 weeks of gestation, who were recruited during maternity clinic visits at Mafraq and Princess Badea hospitals between February and July 2022. Data, including pre- and current gestational weight, height, and pre- and current body weight, were collected through a data collection form, along with a whole blood sample obtained at the clinic to measure fasting plasma glucose (FPG) and HbA1c levels. The Depression, Anxiety, and Stress Scale (DASS) was employed to assess the psychological well-being of the women.

**Results:**

A total of 385 pregnant women participated in the study. The majority of pregnant women experienced a moderate level of depression (77.7%) and anxiety (74.5%), and around half experienced a moderate stress level (44.9%). FPG showed “normal” in 168 (43.6%) participants and “abnormal” in 217 (56.4%). However, the HbA1c level showed “normal” in 190 (49.4%) participants and “abnormal” in 195 (50.6%) with a significant correlation of 0.885. DASS with gestational diabetes mellitus showed a significant difference with a depression factor of p = 0.015, and anxiety factor of p = 0.010, while there was an insignificant difference with stress. One hundred and three (26.8%) participants showed normal weight gain while 282 (73.2%) showed abnormal weight, and there was an insignificant difference between DASS and weight.

**Conclusion:**

The findings show a positive relation between DASS scores and the incidence of gestational diabetes mellitus (GDM). The study emphasizes the importance of further investigation and tailored interventions for depression and anxiety symptoms during pregnancy.

## Introduction

Health problems during pregnancy have attracted a lot of attention from scholars all over the world. However, there is no unified conclusion regarding the effect of psychological well-being on the pregnant population in terms of gestational diabetes mellitus (GDM), gestational weight, and childbirth complications. In a study, anxiety and depression was shown to lead to an increased release of cortisol and insulin resistance, and an increased risk of developing GDM in pregnant women [1]. On the other hand, a previous study believes that anxiety and depression do not increase the incidence of GDM in pregnant women [2].

The previous literature indicated that stress during pregnancy could have a negative impact on prenatal outcomes with excess gestational weight disrupting the hypothalamic-pituitary axis (HPA) [3]. This will lead to a central fat distribution and promotion of intake of nutrient-dense “comfort” food as well as an elevation of cortisol levels. Despite of the potential association, previous studies did not show a consistent association between psychosocial stress during pregnancy and the risk of excess or inadequate gestational weight gain among pregnant women [4].

As reported in the literature, exposure to prenatal psychological issues not only affects the mother’s health but could also have direct and indirect effects on the long-term functioning, the developmental status, and the overall health and well-being of the child [5]. Indeed, the indirect effect of prenatal stressors on the development and health status of a child relates to an increased risk of negative birth outcomes [6]. For instance, stress can predispose mothers to prenatal depression, which affects the postnatal care quality by impeding the interaction between infants and their mothers during the postnatal period [5]. Direct effects of stress on the infant health status include the altering of the neurobiological development of the child [7]. Psychological stress during pregnancy has also been associated with a variety of adverse pregnancy outcomes, including spontaneous preterm birth, neonatal morbidity, and low birth weight [8].

Excessive or inadequate gestational weight gain has serious health consequences for pregnant women and fetuses. Women who gain excessive weight during pregnancy are more likely to have a cesarean section delivery, pre-eclampsia, and excessive weight retention after delivery. On the other hand, inadequate gestational gain can increase the risk of preterm birth. Existing studies have suggested that maternal psychological stress may increase pregnant weight by disrupting the hypothalamic-pituitary axis, resulting in the elevation of cortisol levels and the enhanced intake of nutrients; however, previous observational studies have failed to demonstrate such a relation [4]. Moreover, the wellbeing of the infant may be affected when the mother is subjected to psychosocial stress during her pregnancy. The current knowledge explores the effect of prenatal stress on childhood asthma, eczema, obesity, childhood cancers, and birth defects [9].

In Jordan, the prevalence of depression, anxiety, and stress in the general population is already high [10–13], which makes it reasonable to expect elevated levels among pregnant women. This study is novel in assessing different psychological illnesses in one single analysis and examining their relationships with maternal health. Furthermore, it is the first to explore how antenatal stressors can be addressed by healthcare providers during routine antenatal care in Jordan. The aim of the current study was to evaluate the psychological well-being of pregnant women in Jordan and to investigate the relationship between psychological issues with GDM and gestational weight. This was done because previous studies evaluating the impact of psychological well-being on maternal health have produced conflicting results and because researchers in Jordan have not given enough attention to the consequences of psychological disorders during pregnancy.

## Method

### Study design and setting

The cross-sectional quantitative design was utilized in this study and research participants were recruited during maternity clinic visits at Mafraq (in Mafraq city) and Princess Badea (in Irbid) hospitals between February 20 and July 28, 2022.

### Ethical approval

Before commencing the data collection, ethical approval was obtained from the Research Ethics Committee at Isra University (SREC/22/01/023) and from the Ministry of Health (MoH) under the reference number (1874,14/2/2022). Prior to the administration of the instruments, participants received verbal information related to the nature and purposes of the study and were notified of their right to totally reject participation from the beginning, withdraw at any time during the study, or reject answering any question they did not want to answer. Furthermore, participants were given the chance to ask questions at any time. In addition, participants were asked to read and sign a consent form before completing the instrument. Privacy and confidentiality were maintained as outlined in the consent form. In addition, in this study participants were not asked to include their names within the questionnaire, so the data did not contain any identifiable information.

### Sample size

To achieve a statistically significant sample size, a minimum of 373 patients was determined based on data from a total of 12,768 deliveries conducted at both Mafraq and Princess Badea hospitals in 2021, as reported in the Jordan Ministry of Health’s annual report for that year. The calculations were performed with a 5% margin of error, a 95% confidence level, and an assumed 50% response distribution, utilizing the Raosoft sample size calculator (http://www.raosoft.com/samplesize.html).

### Sampling procedure

A convenience sample was drawn from our target population comprising pregnant women at 24–28 weeks (approximately six and a half months) of gestation who were receiving care at Mafraq and Princess Badea hospitals. Inclusion criteria encompassed pregnant women meeting the following conditions: being at 24–28 weeks of pregnancy, having no pre-gestational psychological disorders, having no previously diagnosed endocrinological illnesses, and informed consent. Exclusion criteria encompassed pregnant women who did not meet the inclusion criteria, those with multiple pregnancies (e.g., twins or triplets), and those who had received treatment with medications known to affect glucose levels, such as corticosteroids.

### Data collection procedure

After obtaining the ethical approval, the researcher scheduled a meeting with the directors of the target hospitals (Mafraq and Princess Badea hospitals) and explained the purpose of the study and data collection procedure. After that, the researcher approached the pregnant women at 24–28 weeks before their maternity clinic appointment through phone calls, provided verbal information about the nature of the study, and asked them to fast in order to collect a fast blood sample. After obtaining written consent, participants were asked to fill out a questionnaire (mentioned below). The instrument took around 20 minutes to complete.

### Study tools

(1) Data collection form

It contained twenty-eight questions on age, educational level, monthly income, husband relationship satisfaction and marital status, planned pregnancy, attitude towards baby’s sex, bad events during pregnancy, social support during pregnancy, and smoking during pregnancy. Moreover, pre- and current gestational weight, height to calculate body mass index (BMI), gestational age, total number of children, depression before pregnancy, obstetric problems before pregnancy, previous abortion, and fertility problems were also recorded. These data were obtained through a direct interview with the participant and via a review of the patient’s record.

(2) Collection of clinical samples for analysis

One whole blood sample was collected at the clinic to test fasting plasma glucose (FPG) as well as HbA1c levels of the patient. Although a 75-g oral glucose tolerance test (OGTT) at 24–28 weeks of pregnancy is the gold standard method in the diagnosis of GDM according to the American Diabetes Association (ADA), it is the HbA1c testing along with a fasting plasma glucose measurement that were currently used in the labs at targeted hospitals. The blood sample was collected from a vein in the arm using a small needle in a lavender tube. Sample withdrawal was performed by the researcher with help from the nursing gynecological teams in the target hospitals. All samples were analyzed directly at clinical laboratories in the target hospitals.

In 2010, ADA included the HbA1c test as a diagnosis criterion for diabetes, and this criterion was endorsed by World Health Organization (WHO) in 2011. The HbA1c may be measured any time of day, has lower biological variation, higher reproducibility, and better analytical stability compared to OGTT, and it is more comfortable for pregnant women than OGTT. The cutoff of HbA1c > 48 mmol/mol (5.8) at 26 weeks (about 6 months) is used for the diagnosis of gestational diabetes in the present study [14]. On the other hand, FPG is convenient to administer, well tolerated, affordable, reliable, reproducible, and has been reported to be little affected during gestation.

Sample collection was performed between 7:00 and 9:00 a.m., and pregnant women with fasting glucose readings exceeding 92 mg/dL were considered to have gestational diabetes mellitus [15]. Nevertheless, for the present research diagnostic judgment, both HbA1c and FPG diagnostic tools were employed to assess GDM. Participants who demonstrated abnormal values in both assessments were classified as having GDM.

(3) Self-report questionnaire

The Depression, Anxiety, and Stress Scale (DASS), a set of three self-report scales, is designed to measure depression, anxiety, and stress [16]. Participants were asked to rate the degree to which each symptom was experienced over the last week on a four-point Likert scale from 0 (did not apply) to 3 (applied to me very much, or much of the time). Each sub-scale in the DASS consists of fourteen items, and scores were calculated by summing the responses for the relevant items [17].

The depression sub-scale consists of items that measure symptoms typically associated with dysphonic mood (e.g., worthlessness) [18]. The anxiety sub-scale includes items that are primarily related to physical arousal, panic attacks, and fear (such as trembling). The stress sub-scale consists of items that measure symptoms such as tension, irritability, and a tendency to overreact to stressful events [18].

Symptom severity scores for depression, anxiety, and stress are provided by the DASS. For depression, a range of scores between 0 and 9 is considered normal, 10–13 mild depression, 14–20 moderate depression, 21–27 severe depression, and 28–42 is considered to have extremely severe depression. The range of scores for anxiety is as follows: normal (0–7), mild (8–9), moderate (10–14), severe anxiety (15–19), and extremely severe anxiety (20–42). For stress, a score between 0 and 14 is normal, 15–18 is mild, 19–25 is moderate, 26–33 is severe, and 34–42 is extremely severe stress [17]. For statistical differences and regression analysis, a depression score of > 9, anxiety score > 7, and stress score > 14 were considered abnormal.

The reliability of the scale was checked by calculating Cronbach’s alpha coefficient for each dimension separately, and the results are shown in Table 1. The values that are greater than 0.60 express sufficient internal consistency to conduct and follow up the analysis [19].

**Table 1:** Cronbach’s alpha coefficient results.

Dimension	Number of items	Cronbach’s alpha coefficient
**Stress scale**	10	0.74
**Depression scale**	21	0.76
**Anxiety scale**	21	0.85

### Statistical data analysis

The Statistical Package of Social Science (SPSS), v 26.0 (SPSS Inc., USA) for analysis was used where the statistical calculations were made. Descriptive statistics of frequencies and percentages were used for categorical variables. A Chi-square test was conducted to explore the difference between categorical groups in terms of normal and abnormal weight gain, gestational diabetes, and no gestational diabetes. A value < 0.05 was considered significant. In addition, the classification of gestational weight gain into two categories, namely “normal gestational weight gain” and “abnormal gestational weight gain”, was determined through the utilization of an online calculator (https://www.calculator.net/pregnancy-weight-gain-calculator.html). This calculator derives a recommended weight gain schedule for a healthy pregnancy, aligning with guidelines established by the Institute of Medicine in their publication titled “Weight Gain During Pregnancy: Reexamining the Guidelines.”

## Results

### Characteristics of the participants

A total of 385 pregnant women participated in the study. As shown in Table 2, there were 68 (17.66%) participants aged from 25 years old and less, and 196 (50.91%) aged 26–35 years. The number of unemployed women was 199 (51.69%), and the number of employed women was 186 (48.31%). Participants with less than a diploma were 162 (42.08%), with a diploma 90 (23.38%), with bachelor’s degree 113 (29.35%), while those with graduate studies were 20 (5.19%). There were 283 (73.51%) participants with a monthly income less than 500 JD and 102 (26.49%) with 500 JD and more. There were 102 (26.49%) participants with a total number of children from 1–3, 193 (50.13%) with 4–7, and 90 (23.38%) with 8 and more. The average gestational age for the sample was between 26 and 35 years, given that the age of marriage in the two regions of Mafraq and Irbid ranged between 23 and 27 years.

**Table 2:** Demographic characteristics of participants (n = 385).

Character	Category	Frequency	Percent
Age (Year)	25 and younger	68	17.66%
	26-35	196	50.91%
	Older than 35	121	31.43%
Employment	Unemployed	199	51.69%
	Employed	186	48.31%
Educational level	Less than Diploma	162	42.08%
	Diploma	90	23.38%
	BSC	113	29.35%
	Graduate Studies	20	5.19%
Monthly Income	Less than 500 JD	283	73.51%
	500 JD and more	102	26.49%
Total children	1-3	102	26.49%
	4-7	193	50.13%
	8 and more	90	23.38%

Overall, the level of education was low for the study sample, and the economic situation was mostly below the poverty level in Jordan, which for a standard family of five in Jordan is 500 dinars per month (based on the poverty level in Jordanian general statistics, 2019). The study sample also showed an increase in fertility and the number of pregnancies.

Table 3 shows the medical and gynecological characteristics of the participants. There were 292 (75.84%) participants satisfied with their marriage. The number of husbands who smoked was 241 (62.60%). Participants who were not satisfied with the sex of their child were 157 (40.78%). Those who have infertility problems were 8 (2.08%). However, 231 (60.00%) participants reported that the duration between their two last pregnancies was less than 2 years, and 154 (40.00%) participants two years and more. Women with planned pregnancies were 175 (45.45%). Previous miscarriages were 59 (15.32%). Smoking during pregnancy was 147 (38.18%) participants while passive smokers were 240 (62.34%). There were 238 (61.82%) with bad events during pregnancy. Those with depression before pregnancy were 137 (35.58%) while those who had anxiety before pregnancy were 81 (21.04%). Emergencies during pregnancy were reported in 53 (13.77%) participants. Women with community support during pregnancy were 146 (37.92%).

**Table 3:** Medical and gynecological characteristics of participants (n = 385).

Character	Category	Frequency	Percent
Marriage satisfaction	Satisfied	292	75.84%
	Not satisfied	93	24.16%
Husband smoking status	Yes	241	62.60%
	No	144	37.40%
Not satisfied with child’s sex	Yes	157	40.78%
	No	228	59.22%
Fertility problems	Yes	8	2.08%
	No	377	97.92%
Duration between two last pregnancies	Less than two years	231	60.00%
	Two years and more	154	40.00%
Emergency case during pregnancy	Yes	53	13.77%
	No	332	86.23%
Previous miscarriage	Yes	59	15.32%
	No	326	84.68%
Smoking during pregnancy	Yes	147	38.18%
	No	238	61.82%
Passive smoking during pregnancy	Yes	240	62.34%
	No	145	37.66%
Bad events during pregnancy (i.e., death of close person)	Yes	180	46.75%
	No	205	53.25%
Social support during pregnancy	Yes	146	37.92%
	No	233	60.52%
Planned pregnancy	Yes	175	45.45%
	No	210	54.55%
Depression before pregnancy	Yes	137	35.58%
	No	248	64.42%
Anxiety before pregnancy	Yes	81	21.04%
	No	304	78.96%

### Depression, anxiety, and stress degree among participants

**Fig 1:**
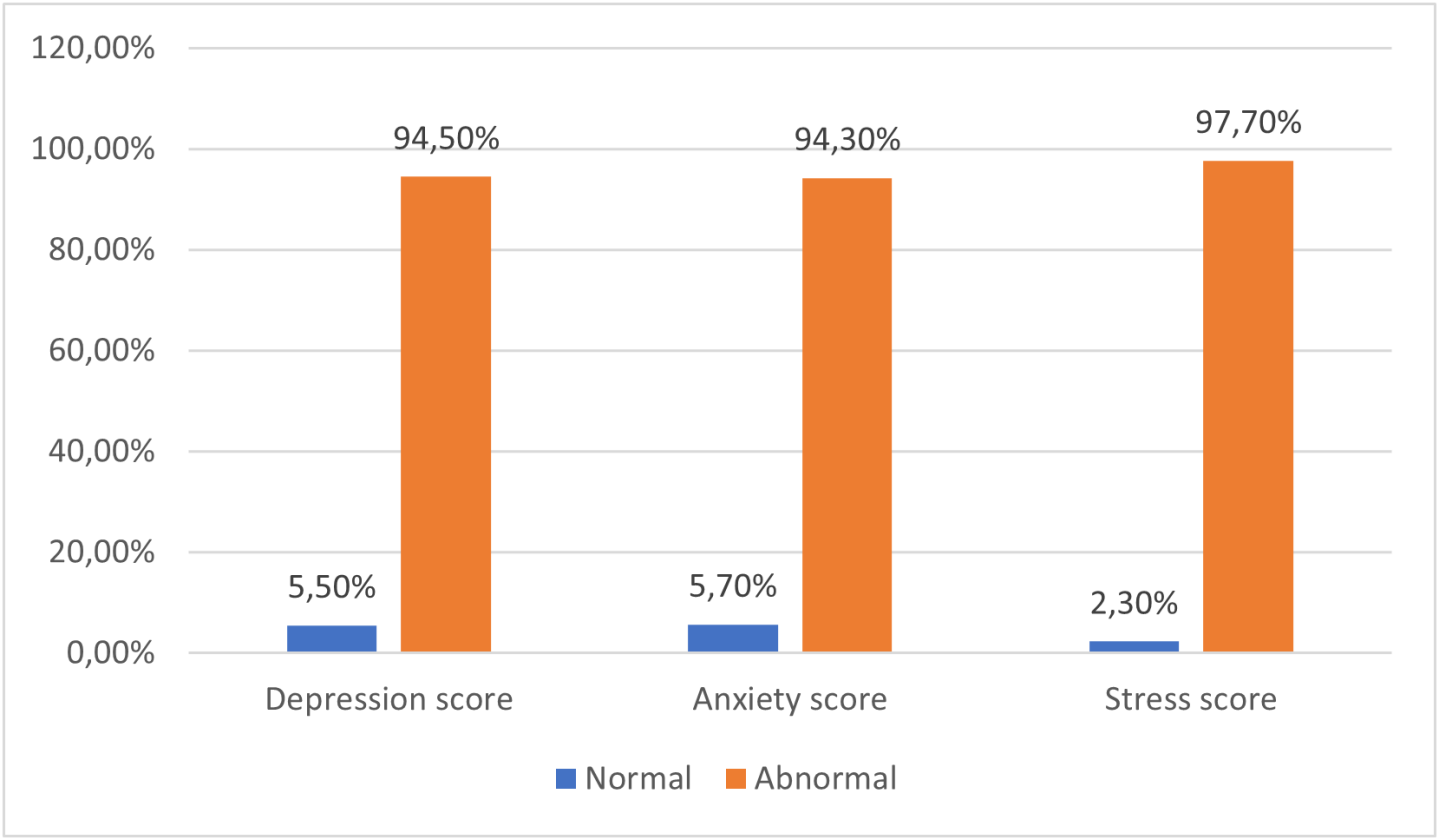
Depression, anxiety, and stress levels.

Table 4 shows that pregnant women’s stress degree varies as it was normal among 9 (2.30%), mild among 50 (13.00%), moderate among 173 (44.90%), severe among 136 (35.30%), and extremely severe among 17 (4.40%). However, depression was normal among 21 (5.50%), mild among 44 (11.40%), moderate among 299 (77.70%), severe among 3 (0.80%), and extremely severe among 18 (4.70%). In addition, anxiety was normal among 22 (5.70%), mild among 44 (11.40%), moderate among 287 (74.5%), severe among 11 (2.9%), and extremely severe among 21 (5.5%). More illustrations for stress, depression, and anxiety levels are portrayed in (Fig 1).

**Table 4:** Depression, anxiety, and stress degree among participants (n = 385).

	Normal	Mild	Moderate	Severe	Extremely Severe
**Stress**	9 (2.30%)	50 (13.00%)	173 (44.90%)	136 (35.30%)	17 (4.40%)
**Depression**	21 (5.50%)	44 (11.40%)	299 (77.70%)	3 (0.80%)	18 (4.70%)
**Anxiety**	22 (5.70%)	44 (11.40%)	287 (74.50%)	11 (2.90%)	21 (5.50%)

### Differences between participants’ characters and DASS dimensions

Cross tabulating the demographic variables versus DASS factors (stress, depression, and anxiety) showed a significant chi square of difference. Only significant results can be seen in Table 5.

**Table 5:** Differences between demographic and medical variables and DASS dimensions.

Variable		Abnormal Stress Score	Abnormal Anxiety Score	Abnormal Depression Score
Employment				
	Unemployed	194 (50.4)	181 (47.0)	182 (47.3)
	Employed	182 (47.3)	182 (47.3)	182 (47.3)
	P-value	0.814	0.004*	0.006*
Educational level				
	Less than diploma	160 (41.6)	158 (41.0)	158 (41.0)
	Diploma	83 (21.6)	83 (21.6)	83 (21.6)
	BSC	113 (29.4)	102 (26.5)	103 (28.3)
	Graduate studies	20 (5.2)	20 (5.2)	20 (5.2)
	P-value	0.001*	0.037*	0.060
Number of children				
	1-3	102 (26.5)	90 (23.4)	90 (23.4)
	4-7	191 (49.6)	187 (48.6)	188 (48.8)
	8 and more	83 (21.6)	86 (22.3)	86 (22.3)
	P-value	< 0.001*	0.008*	0.004*
Marriage satisfaction				
	Satisfied	283 (73.5)	270 (70.1)	271 (70.4)
	Not satisfied	93(24.2)	93 (24.2)	93 (24.2)
	P-value	0.087	0.006*	0.008*
Husband smoking status				
	Yes	232 (60.3)	229 (59.5)	229 (59.5)
	No	144 (37.4)	134 (34.8)	135 (35.1)
	P-value	0.019*	0.421	0.595
Not satisfied with child’s sex				
	Yes	149 (38.7)	157 (40.8)	157 (40.8)
	No	227 (59.0)	206 (53.5)	207 (53.8)
	P-value	0.003*	< 0.001*	< 0.001*
Duration between two last pregnancies				
	Less than two years	230 (59.7)	219 (56.9)	219 (56.9)
	Two years and more	146 (37.9)	144 (37.4)	145 (37.7)
	P-value	0.002*	0.591	0.783
Smoking during pregnancy				
	Yes	146 (37.9)	147 (38.2)	147 (38.2)
	No	230 (59.7)	216 (56.1)	217 (56.4)
	P-value	0.091	< 0.001*	< 0.001*
Bad events during pregnancy				
	Yes	171 (44.4)	167 (43.4)	168 (43.6)
	No	205 (53.2)	196 (50.9)	196 (50.9)
	P-value	0.001*	0.232	0.326
Planned pregnancy				
	Yes	175 (45.5)	160 (41.6)	161 (41.8)
	No	201 (52.2)	203 (52.7)	203 (52.7)
	P-value	0.006*	0.027*	0.045*

* Significance level, p < 0.05.

### Differences between DASS dimensions and gestational diabetes

**Fig 2:**
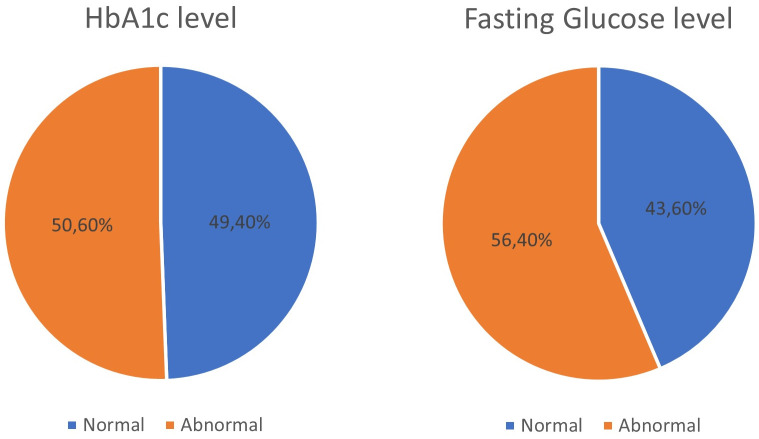
FPG and HbA1c levels.

FPG showed “normal” in 168 (43.6%) participants and “abnormal” in 217 (56.4%). However, HbA1c level showed “normal” in 190 (49.4%) participants and “abnormal” in 195 (50.6%) as shown in (Fig 2). FPG at a cut-off point 95 mg/dL and HbA1c level at a cut-off point of 5.8% showed a significant correlation of 0.885 at the 0.01 level (2-tailed).

Nevertheless, the study considered a case to be gestational if both HbA1c and FPG were abnormal. As such, Table 6 shows the frequency and percentages of gestational diabetes in the sample. Cross tabulation of DASS with GDM showed a significant difference with the depress factor with p = 0.015, and anxiety with p = 0.010 while an insignificant difference with the stress as the p value is 0.167.

**Table 6:** Gestational diabetes mellitus among participants.

	Frequency	Percent
Gestational Diabetes	168	43.6
No Gestational Diabetes	217	56.4
Total	385	100.0

### Differences between DASS dimensions and gestational weight gain

**Fig 3:**
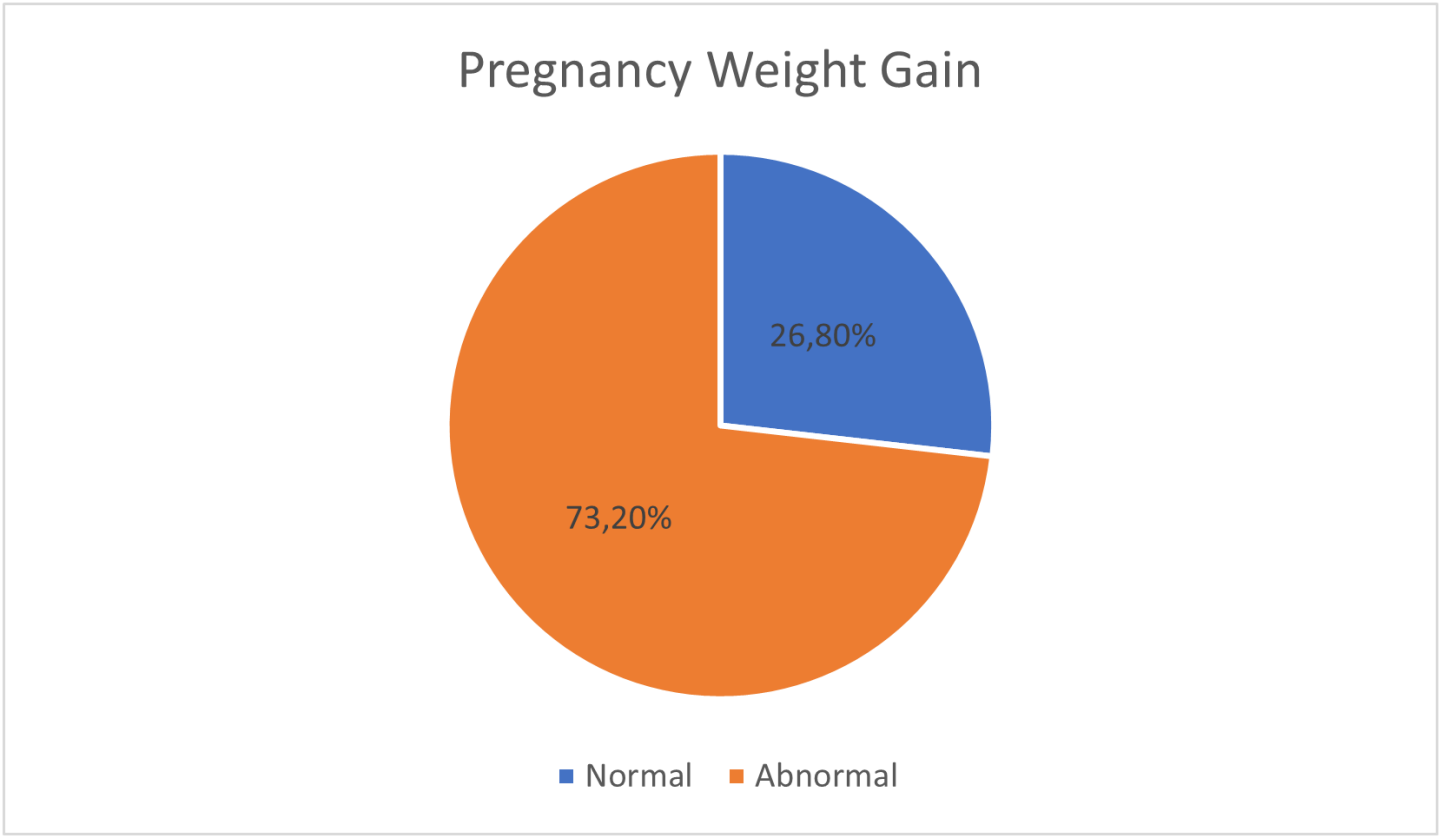
Normal versus abnormal gestational weight gain.

Pregnancy weight gain varies among participants in this study; the highest ratio was for the abnormal weight gain during pregnancy. Nevertheless, 103 (26.8%) of the participants showed normal weight gain, and 282 (73.2%) of the participants showed abnormal weight, as shown in Fig 3. The relationship of DASS and weight showed a Pearson Chi-Square value of 1.468 and a P value of 0.480, which is not significant.

## Discussion

The findings of this study are significant as they provide a comprehensive assessment of antenatal psychological factors in terms of depression, anxiety, and stress among pregnant women in Jordan. This study offers new insights into how psychological factors collectively impact maternal health. Furthermore, the study highlights the vital role of healthcare providers in identifying and addressing these factors during routine antenatal care, emphasizing the potential for targeted interventions to improve pregnant women’s health and pregnancy outcomes.

The results shed light on the complex relationship between maternal mental health and specific pregnancy-related outcomes. Analysis showed that most pregnant women had a moderate to severe degree of stress while most had moderate depression and anxiety. Indeed, these results are in line with most of the other studies which confirmed the existence of psychological factors, such as stress, anxiety, and depression, which have been reported as common among pregnant women [20]. In fact, this is quite in line with the findings of most of the previous studies, as the term “psychosocial stress” describes the stressful issues that happen which, according to Sosnowski et al. [21], include but are not limited to changes in family makeup, domestic violence, housing, employment status, and personal life that need adaptive coping behavior on the part of the affected individual [5]. Not only this, but Lautarescu et al. [6] stated that “pregnancy-specific” stress is directly associated with the pregnancy itself, for example, nervousness regarding the life changes that will come with motherhood, concerns about the health status of the infant, and the results of parental screening, which all support the findings of this study.

In our study, employed pregnant women had higher anxiety and depression scores; this result was in contrast to a previous cross-sectional study in a hospital of Bobal University, where higher anxiety was found among unemployed women [22]. Moreover, a previous Korean study found that being unemployed, pregnant, and having a low family income were risk factors for depression [23]. Our results can be justified by the double responsibilities of work and home, which can mean more pressure on working mothers.

Stress and anxiety were significant in our research among women with higher education, but Deklava et al. [24] concluded that a low level of education reinforces the psychological stress and anxiety during the pregnancy, which contradicts our results. We assume that women with higher education are knowledgeable and aware of the complications of gestational diabetes and abnormal weight gain during pregnancy on their and their children’s health.

Women with a higher number of children had an irritated emotional status and were related to higher anxiety levels in our study. Similarly, Biaggi et al. [25] found that multiparas women tended to be depressed and anxious more than primigravida. Again, this can be explained by the fact that mothers with many children are taking care of the basics, making sure children are fed, and getting children to/from activities while also getting housework done.

Smoking during pregnancy was related to anxiety and depression in our research. Since smoking during pregnancy is a complex phenomenon, it is found that such behavior is influenced by psychological context at multiple stages [26]. Additionally, passive smoke affects pregnancy outcomes for women and their infants, and women who smoke and are exposed to passive smoking (especially husband smoking) have significantly higher levels of depressive and stress symptoms [27]. This finding is compatible with our study, and their concerns about the harmful effects of smoking can explain their high levels of stress.

Bad events during pregnancy elevated the stress score significantly in our work; such a finding is compatible with Hedegaard, et al. [28] who showed that women who had one or more stressful events had a risk for stress related outcomes such as preterm delivery.

Unplanned pregnancy was related to higher DASS scores than planned ones; this comes in line with a previously published study in Malaysia in which a positive association was found between psychological stress and unplanned pregnancy [29]. Another study found that unplanned pregnancy caused a negative psychological effect which resulted in missing prenatal clinical visits and postpartum care [30].

Not only unplanned pregnancies but also low marital satisfaction caused psychological stress in terms of depression and anxiety in the present study. Similarly, Chen et al. found a fourfold risk for antenatal psychological depression and stress among women with low satisfaction with their marriage [31].

The sex of the child was related to stress, depression, and anxiety among participating women. Specifically, mothers of girls reported higher levels compared to mothers of boys. This may be explained by the cultural preference in some communities. In the Indian population, for example, a study showed the preference for male children over female children [32]. Although discrimination between sexes is prohibited in Islam (the popular religion in Jordan), it is the sociocultural context that controls this attitude.

In addition, stress is related to increased HbA1c and eventually FPG, thus representing important risk factors for GDM development [33]. More than half of the women in the present study showed abnormal FPG and HbA1c levels. A pilot study showed GDM women had twice the depression score of DASS [34], and this could be justified by the imbalanced levels of hormones that affect blood glucose levels. Also, Egan found that women with GDM had significantly elevated overall DASS scores [35].

A few pregnant women showed normal weight gain during pregnancy. In fact, excess or inadequate gestational weight gain has serious health consequences for pregnant women and fetal health. On the other hand, inadequate gestational gain can increase the risk of preterm birth. In a study conducted by Gilbert et al. among women with GDM, it was found that an elevated depression score was associated with weight gain [36]. In addition, Kominiarek and colleagues found a strong relationship between stress during pregnancy and gestational weight gain [37]. However, there was an insignificant difference between DASS scores and gestational weight in our study, conflicting with Hartly et al. who show psychosocial screening by a healthcare provider may help to identify women who are at risk of excessive gestational weight gain [38].

The present study has several limitations, including a relatively small sample size, potential bias due to recruitment from only two maternity clinics, and reliance on self-reported measures of depression, anxiety, and stress, which may be subject to reporting bias.

## Conclusion

In conclusion, the study’s findings indicate a noteworthy positive relation between DASS scores and the incidence of GDM in the target hospitals. It is therefore recommended that healthcare providers consider implementing routine mental health screenings for pregnant women to identify individuals at risk of elevated DASS scores. Additionally, healthcare practitioners should collaborate with mental health professionals to provide appropriate support and interventions for pregnant women experiencing symptoms of depression or anxiety.

Future research, including larger, multicenter cohorts and longitudinal follow-up to assess the long-term effects of antenatal depression, anxiety, and stress levels on both maternal and neonatal health is recommended.

## Supporting information

S1 File Support document. (PDF)
