# Supplementary material for: The effect of antenatal psychological well-being on maternal health status
Source: PLoS One. 2025 Nov 14;20(11):e0336684. doi: 10.1371/journal.pone.0336684 (PMC12617956; doi:10.1371/journal.pone.0336684)
Supplement: S1 File — (PDF) [file pone.0336684.s001.pdf]

Participant#	AGEC	Job	EduLevel	EmploStatus	MonthIncome	MarriSatisfaction
1	2.00	2	1	1	1	1
2	2.00	1	2	2	1	1
3	2.00	2	1	1	1	1
5	2.00	2	3	1	2	1
6	3.00	2	1	1	2	1
7	2.00	1	3	2	1	2
8	2.00	1	3	2	1	1
9	1.00	1	1	2	1	1
10	1.00	1	3	2	1	1
11	1.00	1	2	2	1	2
12	2.00	1	1	2	1	1
13	1.00	1	3	2	1	1
14	2.00	2	2	1	1	1
15	3.00	2	2	1	2	1
16	3.00	2	1	1	1	1
17	3.00	1	1	2	1	2
18	2.00	1	4	2	1	1
19	2.00	1	2	2	1	1
21	1.00	1	3	2	1	1
22	3.00	2	3	1	2	1
23	3.00	1	1	2	1	2
24	2.00	2	4	1	2	1
25	2.00	1	3	2	1	1
26	3.00	1	1	2	1	2
27	1.00	1	3	2	1	1
28	2.00	2	2	1	1	2
29	2.00	2	1	1	2	1
30	2.00	2	2	1	1	1
31	3.00	1	1	2	1	2
32	1.00	1	2	2	1	1
33	2.00	1	3	2	1	1
34	3.00	1	1	2	1	1
35	3.00	1	1	2	1	1
36	2.00	2	3	1	1	1
37	3.00	2	1	1	1	2
38	2.00	1	1	2	1	1
39	2.00	2	2	1	2	1
40	2.00	2	3	1	2	1
41	2.00	2	1	1	1	1
42	2.00	2	3	1	1	2
43	3.00	1	2	2	1	1
44	2.00	2	2	1	1	2
45	2.00	1	2	2	1	1
46	1.00	2	3	1	1	1
47	3.00	1	2	1	2	2
48	3.00	2	1	2	1	2
49	1.00	2	4	1	1	1
50	2.00	1	1	2	2	1
51	3.00	2	3	1	2	1
52	2.00	1	1	2	1	1
53	1.00	1	3	2	1	1

54	2.00	2	3	2	1	1
55	3.00	1	1	1	2	2
56	2.00	1	1	1	1	1
57	2.00	2	3	1	2	1
58	2.00	2	1	1	2	1
59	3.00	1	1	2	1	2
60	3.00	2	1	1	2	1
61	3.00	1	1	2	1	1
62	3.00	1	2	2	1	1
63	2.00	2	1	1	1	2
64	2.00	2	1	1	2	1
65	2.00	2	2	2	1	1
66	2.00	2	3	2	1	2
67	2.00	1	3	2	1	1
68	3.00	1	2	2	1	1
69	2.00	1	2	2	1	1
70	3.00	1	1	2	1	2
71	2.00	1	2	2	1	1
72	2.00	2	3	2	1	2
73	2.00	1	3	2	1	1
74	3.00	2	2	2	1	1
75	2.00	1	2	2	1	1
76	3.00	1	1	2	1	2
77	2.00	1	2	2	1	1
78	2.00	1	3	2	1	2
79	2.00	1	3	2	1	1
80	3.00	1	2	2	1	1
81	2.00	1	2	2	1	1
82	3.00	2	1	2	1	2
83	2.00	2	2	2	1	1
84	2.00	2	3	2	1	2
85	2.00	1	3	2	1	1
86	3.00	1	2	2	1	1
87	2.00	1	2	2	1	1
88	3.00	1	1	2	1	2
89	2.00	1	2	2	1	1
90	2.00	1	3	2	1	2
91	2.00	1	3	2	1	1
92	3.00	1	2	2	1	1
93	2.00	1	2	2	1	1
94	3.00	2	1	2	1	2
95	2.00	2	2	2	1	1
96	2.00	2	3	2	1	2
97	2.00	1	3	2	1	1
98	3.00	1	2	1	2	2
99	2.00	1	1	1	1	1
100	3.00	2	2	1	2	2
101	2.00	1	1	1	1	1
102	3.00	2	1	1	2	2
103	2.00	1	1	1	1	1
104	3.00	1	2	1	2	2
105	2.00	2	1	1	1	1

106	3.00	2	2	1	2	2
107	2.00	2	1	1	1	1
108	2.00	2	3	1	1	2
109	2.00	2	3	2	1	1
110	2.00	2	3	1	1	2
111	2.00	2	3	2	1	1
112	2.00	2	3	1	1	2
113	2.00	2	3	2	1	1
114	2.00	2	3	1	1	2
115	2.00	2	3	2	1	1
116	2.00	2	3	1	1	2
117	2.00	2	3	2	1	1
118	2.00	2	1	1	1	1
119	1.00	2	4	1	1	1
120	2.00	2	1	1	1	1
121	1.00	2	4	1	1	1
122	2.00	2	1	1	1	1
123	1.00	2	4	1	1	1
124	2.00	2	1	1	1	1
125	1.00	2	4	1	1	1
126	2.00	2	1	1	1	1
127	1.00	2	4	1	1	1
128	3.00	2	3	1	2	1
129	1.00	2	3	1	1	1
130	3.00	2	3	1	2	1
131	1.00	2	3	1	1	1
132	3.00	2	3	1	2	1
133	1.00	2	3	1	1	1
134	3.00	2	3	1	2	1
135	1.00	2	3	1	1	1
136	3.00	2	3	1	2	1
137	1.00	2	3	1	1	1
138	3.00	2	3	1	2	1
139	1.00	1	3	2	1	1
140	1.00	1	2	2	1	2
141	1.00	1	3	2	1	1
142	3.00	1	1	2	1	2
143	2.00	1	2	2	1	1
145	1.00	1	3	2	1	1
146	3.00	1	1	2	1	2
147	2.00	1	3	2	1	1
148	3.00	1	1	2	1	2
149	1.00	1	3	2	1	1
150	3.00	1	2	2	1	2
151	1.00	1	2	2	1	1
152	3.00	1	3	2	1	1
153	3.00	1	1	2	1	1
154	3.00	1	1	2	1	1
155	2.00	1	1	2	1	1
156	2.00	1	1	2	1	1
157	3.00	1	1	1	2	2
158	3.00	1	1	2	1	2

159	1.00	1	3	2	1	1
160	1.00	1	2	2	1	2
161	1.00	1	3	2	1	1
162	3.00	1	1	2	1	2
163	2.00	1	2	2	1	1
165	1.00	1	3	2	1	1
166	3.00	1	1	2	1	2
167	2.00	1	3	2	1	1
168	3.00	1	1	2	1	2
169	1.00	1	3	2	1	1
170	3.00	1	2	2	1	2
171	1.00	1	2	2	2	1
172	3.00	1	3	2	1	1
173	3.00	1	1	2	1	1
174	3.00	1	1	2	1	1
175	2.00	1	1	2	1	1
176	2.00	1	1	2	1	1
177	3.00	1	1	1	2	2
178	3.00	1	1	2	1	2
179	3.00	1	1	2	1	1
180	3.00	1	2	2	1	1
181	1.00	1	3	2	1	1
182	1.00	1	2	2	1	2
183	1.00	1	3	2	1	1
184	3.00	1	1	2	1	2
185	2.00	1	2	2	1	1
187	1.00	1	3	2	1	1
188	3.00	1	1	2	1	2
189	2.00	1	3	2	1	1
190	3.00	1	1	2	1	2
191	1.00	1	3	2	1	1
192	3.00	1	2	2	1	2
193	1.00	1	2	2	2	1
194	3.00	1	3	2	1	1
195	3.00	1	1	2	1	1
196	3.00	1	1	2	1	1
197	2.00	1	1	2	1	1
198	2.00	1	1	2	1	1
199	3.00	1	1	1	2	2
200	1.00	1	3	2	1	1
201	1.00	1	2	2	1	2
202	1.00	1	3	2	1	1
203	3.00	1	1	2	1	2
204	2.00	1	2	2	1	1
206	1.00	1	3	2	1	1
207	3.00	1	1	2	1	2
208	2.00	1	3	2	1	1
209	3.00	1	1	2	1	2
210	1.00	1	3	2	1	1
211	3.00	1	2	2	1	2
212	1.00	1	2	2	2	1
213	3.00	1	3	2	1	1

214	3.00	1	1	2	1	1
215	3.00	1	1	2	1	1
216	2.00	1	1	2	1	1
217	2.00	1	1	2	1	1
218	3.00	1	1	1	2	2
219	3.00	1	1	2	1	2
220	3.00	1	1	2	1	1
221	3.00	1	2	2	1	1
222	1.00	1	3	2	1	1
223	1.00	1	2	2	1	2
224	1.00	1	3	2	1	1
225	3.00	1	1	2	1	2
226	2.00	1	2	2	1	1
228	1.00	1	3	2	1	1
229	3.00	1	1	2	1	2
230	2.00	1	3	2	1	1
231	3.00	1	1	2	1	2
232	1.00	1	3	2	1	1
233	3.00	1	2	2	1	2
234	1.00	1	2	2	2	1
235	1.00	1	3	2	1	1
236	3.00	1	1	2	1	1
237	3.00	1	1	2	1	1
238	2.00	1	1	2	1	1
239	2.00	1	1	2	1	1
240	3.00	1	1	1	2	2
241	3.00	1	1	2	1	2
242	3.00	1	1	2	1	1
243	3.00	1	2	2	1	1
244	1.00	1	3	2	1	1
245	1.00	1	2	2	1	2
246	1.00	1	3	2	1	1
247	3.00	1	1	2	1	2
248	2.00	1	2	2	1	1
250	1.00	1	3	2	1	1
251	3.00	1	1	2	1	2
252	2.00	1	3	2	1	1
253	3.00	1	1	2	1	2
254	1.00	1	3	2	1	1
255	2.00	2	3	1	2	1
256	3.00	2	1	1	1	2
257	2.00	2	3	1	2	1
258	3.00	2	1	1	1	2
259	2.00	2	3	1	2	1
260	3.00	2	1	1	1	2
261	2.00	2	3	1	2	1
262	3.00	2	1	1	1	2
263	2.00	2	3	1	2	1
264	3.00	2	1	1	1	2
265	2.00	2	3	1	2	1
267	2.00	1	1	2	1	1
269	2.00	1	1	2	1	1

271	2.00	1	1	2	1	1
273	2.00	1	1	2	1	1
275	2.00	1	1	2	1	1
277	2.00	1	1	2	1	1
278	1.00	1	1	2	1	1
279	2.00	1	4	2	1	1
280	1.00	1	1	2	1	1
281	2.00	1	4	2	1	1
282	1.00	1	1	2	1	1
283	2.00	1	4	2	2	1
284	1.00	1	1	2	1	1
285	2.00	1	4	2	2	1
286	1.00	1	1	2	1	1
287	2.00	1	4	2	2	1
288	1.00	1	1	2	1	1
289	2.00	1	4	2	1	1
290	2.00	1	1	2	2	1
291	2.00	1	1	2	2	1
292	2.00	1	1	2	2	1
293	2.00	1	1	2	2	1
294	2.00	1	1	2	2	1
295	1.00	1	3	2	1	1
296	1.00	1	3	2	1	1
297	1.00	1	3	2	1	1
298	1.00	1	3	2	1	1
299	1.00	1	3	2	1	1
300	2.00	2	1	1	1	1
301	2.00	2	1	1	2	1
302	2.00	2	2	1	2	1
303	2.00	2	1	1	2	1
304	2.00	2	1	1	1	1
305	2.00	2	1	1	2	1
306	2.00	2	2	1	2	1
307	2.00	2	1	1	2	1
308	2.00	2	1	1	1	1
309	2.00	2	1	1	2	1
310	2.00	2	2	1	2	1
311	2.00	2	1	1	2	1
312	2.00	2	1	1	1	1
313	2.00	2	1	1	2	1
314	2.00	2	2	1	2	1
315	2.00	2	1	1	2	1
316	2.00	2	1	1	2	1
317	2.00	2	1	1	1	1
318	2.00	2	1	1	2	1
319	2.00	2	2	1	2	1
320	2.00	2	1	1	2	1
321	2.00	2	1	1	2	1
322	2.00	2	1	1	1	1
323	3.00	2	1	1	1	1
324	3.00	2	1	1	1	1
325	2.00	2	1	1	1	1

326	1.00	2	1	1	1	1
327	3.00	2	1	1	1	1
328	3.00	2	1	1	1	1
329	2.00	2	3	1	2	1
330	2.00	2	3	1	2	1
331	2.00	2	3	1	2	1
332	2.00	2	3	1	2	1
333	2.00	2	3	1	2	1
334	2.00	2	3	1	2	1
335	2.00	2	3	1	2	1
336	2.00	2	3	1	2	1
337	2.00	2	3	1	2	1
338	2.00	2	3	1	2	1
339	2.00	2	2	1	1	2
340	2.00	2	2	1	1	2
341	2.00	2	2	1	1	2
342	2.00	2	2	1	1	2
343	2.00	2	2	1	1	2
344	3.00	2	2	1	2	1
345	3.00	2	2	1	2	1
346	3.00	2	2	1	2	1
347	3.00	2	2	1	2	1
348	3.00	2	2	1	2	1
349	3.00	2	2	1	2	1
350	2.00	2	2	1	1	2
351	2.00	2	2	1	1	2
352	2.00	2	1	1	1	2
353	2.00	2	2	1	1	2
354	2.00	2	2	1	1	2
355	2.00	2	1	1	1	2
356	2.00	2	2	1	1	2
357	2.00	2	1	1	1	2
358	2.00	2	2	1	1	2
359	2.00	2	2	1	1	1
360	2.00	2	2	1	1	1
361	2.00	2	2	1	1	1
362	2.00	2	2	1	1	1
363	2.00	2	3	1	1	1
364	2.00	2	2	1	1	1
365	2.00	2	2	1	1	1
366	2.00	2	2	1	1	1
367	2.00	2	2	1	1	1
368	2.00	2	2	1	1	1
369	2.00	2	1	1	1	1
370	2.00	2	1	1	1	1
372	2.00	2	4	1	2	1
373	3.00	2	3	1	2	1
374	2.00	2	1	1	1	1
375	2.00	2	1	1	1	1
376	3.00	2	1	1	2	1
377	2.00	2	4	1	2	1
378	3.00	2	3	1	2	1

379	3.00	2	1	1	2	1
380	2.00	2	1	1	1	1
381	2.00	2	1	1	1	1
382	3.00	2	1	1	2	1
383	2.00	2	4	1	2	1
384	3.00	2	3	1	2	1
385	2.00	2	1	1	1	1
386	2.00	2	1	1	1	1
387	3.00	2	1	1	2	1
388	2.00	2	4	1	2	1
389	3.00	2	3	1	2	1
390	3.00	2	1	1	2	1
391	2.00	2	1	1	1	1
392	2.00	2	1	1	1	1
393	3.00	2	1	1	2	1
394	2.00	2	4	1	2	1
395	3.00	2	3	1	2	1
396	3.00	2	1	1	2	1
397	2.00	2	1	1	1	1
398	2.00	2	1	1	1	1
399	3.00	2	1	1	2	1
400	2.00	2	4	1	2	1

TOTAL_CHILDR	SmokSpouce	NotsatisChildSex	ProblemsInfertality	DepBefPregnancy
2.00	1	1	2	2
3.00	1	1	2	2
2.00	2	1	2	2
1.00	2	1	2	1
3.00	2	2	2	2
1.00	1	2	2	1
2.00	2	2	2	1
2.00	1	2	2	2
1.00	1	2	2	1
2.00	1	2	2	1
2.00	1	2	2	2
1.00	1	1	2	1
2.00	2	2	2	2
3.00	2	2	2	1
2.00	1	1	2	2
3.00	1	1	2	2
2.00	1	2	2	2
1.00	1	2	2	2
1.00	2	2	2	2
2.00	1	2	2	1
2.00	1	1	2	2
1.00	2	2	2	2
2.00	1	2	2	2
2.00	1	1	2	2
1.00	2	2	2	1
1.00	1	1	2	1
2.00	1	1	2	2
2.00	2	2	2	1
2.00	2	1	1	2
1.00	1	2	2	1
2.00	2	2	2	1
2.00	1	2	2	2
3.00	1	2	2	2
3.00	2	1	2	2
1.00	1	1	2	2
2.00	1	1	2	2
2.00	2	2	2	2
2.00	2	2	2	2
1.00	1	2	2	2
3.00	1	1	2	2
3.00	2	2	2	1
2.00	1	1	2	1
2.00	2	2	2	1
1.00	1	2	2	1
3.00	1	2	2	1
3.00	1	1	2	2
2.00	2	2	2	1
2.00	1	1	2	2
2.00	2	2	2	1
3.00	1	1	2	2
1.00	1	2	2	1

2.00	1	1	2	1
3.00	1	2	2	2
3.00	2	1	2	2
1.00	2	1	2	1
2.00	1	2	2	2
3.00	1	1	2	2
2.00	2	2	2	2
3.00	2	2	2	2
3.00	2	2	2	2
2.00	1	1	2	2
2.00	1	2	2	2
3.00	1	1	2	2
1.00	1	2	2	1
2.00	2	2	2	2
3.00	2	2	2	1
2.00	2	2	2	1
2.00	1	1	2	2
3.00	1	1	2	2
1.00	1	2	2	1
2.00	2	2	2	2
3.00	2	2	2	1
2.00	2	2	2	1
2.00	1	1	2	2
3.00	1	1	2	2
1.00	1	2	2	1
2.00	2	2	2	2
3.00	2	2	2	1
2.00	2	2	2	1
3.00	1	1	2	2
3.00	1	1	2	2
1.00	1	2	2	1
2.00	2	2	2	2
3.00	2	2	2	1
2.00	2	2	2	1
2.00	1	1	2	2
3.00	1	1	2	2
1.00	1	2	2	1
2.00	2	2	2	2
3.00	2	2	2	1
2.00	2	2	2	1
3.00	1	1	2	2
3.00	1	1	2	1
1.00	1	2	2	1
2.00	2	2	2	1
3.00	1	2	2	1
3.00	2	1	2	2
3.00	1	2	2	1
3.00	2	1	2	2
3.00	1	2	2	2
3.00	2	1	2	2
3.00	1	2	2	1
3.00	2	1	2	2

3.00	1	2	2	1
3.00	2	1	2	2
3.00	1	1	2	2
2.00	1	1	2	1
3.00	1	1	2	2
2.00	1	1	2	1
3.00	1	1	2	2
2.00	1	1	2	1
3.00	1	1	2	2
2.00	1	1	2	1
3.00	1	1	2	2
2.00	1	1	2	1
1.00	1	2	2	2
2.00	2	2	2	1
1.00	1	2	2	2
2.00	2	2	2	1
1.00	1	2	2	2
2.00	2	2	2	1
1.00	1	2	2	2
2.00	2	2	2	1
1.00	1	2	2	2
2.00	2	2	2	1
2.00	1	2	2	2
1.00	1	2	2	1
2.00	1	2	2	2
1.00	1	2	2	1
2.00	1	2	2	2
1.00	1	2	2	1
2.00	1	2	2	2
1.00	1	2	2	1
2.00	1	2	2	2
1.00	1	2	2	1
2.00	1	2	2	2
1.00	1	2	2	1
1.00	1	2	2	1
1.00	1	1	2	1
3.00	1	1	2	2
1.00	1	2	2	2
1.00	2	2	2	2
2.00	1	1	2	2
2.00	1	2	2	2
2.00	1	1	2	2
2.00	2	2	2	1
2.00	2	1	1	2
1.00	1	2	2	1
2.00	2	2	2	1
2.00	1	2	2	2
3.00	1	2	2	2
2.00	1	1	2	2
3.00	1	1	2	2
3.00	1	2	2	2
3.00	1	1	2	2

1.00	1	2	2	1
1.00	1	2	2	1
1.00	1	1	2	1
1.00	1	1	2	2
1.00	1	2	2	2
1.00	2	2	2	2
2.00	1	1	2	2
2.00	1	2	2	2
2.00	1	1	2	2
2.00	2	2	2	1
2.00	2	1	1	2
1.00	1	2	2	1
2.00	2	2	2	1
2.00	1	2	2	2
3.00	1	2	2	2
2.00	1	1	2	2
3.00	1	1	2	2
3.00	1	2	2	2
2.00	1	1	2	2
3.00	2	2	2	2
3.00	2	2	2	2
1.00	1	2	2	1
1.00	1	2	2	1
1.00	1	1	2	1
3.00	1	1	2	2
1.00	1	2	2	2
1.00	2	2	2	2
3.00	1	1	2	2
2.00	1	2	2	2
2.00	1	1	2	2
2.00	2	2	2	1
2.00	1	1	1	2
1.00	1	2	2	1
1.00	2	2	2	1
2.00	1	2	2	2
3.00	1	2	2	2
2.00	1	1	2	2
3.00	1	1	2	2
3.00	1	2	2	2
1.00	1	2	2	1
1.00	1	2	2	1
1.00	1	1	2	1
3.00	1	1	2	2
1.00	1	2	2	2
1.00	2	2	2	2
2.00	1	1	2	2
2.00	1	2	2	2
2.00	1	1	2	2
2.00	2	2	2	1
2.00	1	1	1	2
1.00	1	2	2	1
2.00	2	2	2	1

2.00	1	2	2	2
3.00	1	2	2	2
2.00	1	1	2	2
3.00	1	1	2	2
3.00	1	2	2	2
2.00	1	1	2	2
3.00	2	2	2	2
3.00	2	2	2	2
2.00	1	2	2	1
2.00	1	2	2	1
1.00	1	1	2	1
3.00	1	1	2	2
1.00	1	2	2	2
1.00	2	2	2	2
3.00	1	1	2	2
2.00	1	2	2	2
2.00	1	1	2	2
2.00	2	2	2	1
2.00	1	1	2	2
2.00	1	2	2	1
2.00	2	2	2	1
2.00	1	2	2	2
3.00	1	2	2	2
2.00	1	1	2	2
3.00	1	1	2	2
3.00	1	2	2	2
3.00	1	1	2	2
3.00	2	2	2	2
3.00	2	2	2	2
1.00	1	2	2	1
1.00	1	2	2	1
1.00	1	1	2	1
3.00	2	1	2	2
1.00	1	2	2	2
1.00	2	2	2	2
2.00	1	1	2	2
2.00	1	2	2	2
2.00	1	1	2	2
2.00	2	2	2	1
1.00	2	1	2	1
1.00	1	1	2	2
1.00	2	1	2	1
1.00	1	1	2	2
1.00	2	1	2	1
1.00	1	1	1	2
1.00	2	1	2	1
1.00	1	1	1	2
1.00	2	1	2	1
1.00	1	1	1	2
1.00	2	1	2	1
2.00	1	2	2	2
2.00	1	2	2	2

1.00	1	2	2	2
2.00	1	2	2	2
2.00	1	2	2	2
2.00	1	2	2	2
2.00	1	2	2	2
2.00	1	2	2	2
2.00	1	2	2	2
2.00	1	2	2	2
2.00	1	2	2	2
3.00	1	2	2	2
2.00	1	2	2	2
2.00	1	2	2	2
2.00	1	2	2	2
2.00	1	2	2	2
2.00	1	2	2	2
2.00	1	2	2	2
2.00	1	1	2	2
2.00	1	1	2	2
2.00	1	1	2	2
2.00	1	1	2	2
2.00	1	1	2	2
1.00	1	2	2	1
1.00	1	2	2	1
1.00	1	2	2	1
1.00	1	2	2	1
1.00	1	2	2	1
2.00	2	2	2	2
2.00	1	1	2	2
2.00	2	2	2	2
2.00	1	2	2	2
2.00	2	2	2	2
2.00	1	1	2	2
2.00	2	2	2	2
2.00	1	2	2	2
2.00	2	2	2	2
2.00	1	1	2	2
2.00	2	2	2	2
2.00	1	2	2	2
2.00	2	2	2	2
2.00	1	1	2	2
2.00	2	2	2	2
2.00	1	2	2	2
2.00	1	2	2	2
2.00	2	2	2	2
2.00	1	1	2	2
2.00	2	2	2	2
2.00	1	2	2	2
2.00	1	2	2	2
2.00	2	2	2	2
2.00	1	1	2	2
2.00	1	1	2	2
3.00	1	1	2	2

1.00	1	1	2	2
2.00	1	1	2	2
3.00	1	1	2	2
1.00	2	2	2	2
1.00	2	1	2	1
1.00	2	2	2	2
1.00	2	1	2	1
1.00	2	2	2	2
1.00	2	1	2	1
2.00	2	2	2	2
1.00	2	1	2	1
2.00	2	2	2	2
2.00	2	1	2	1
2.00	1	1	2	1
2.00	1	1	2	1
2.00	1	1	2	1
2.00	1	1	2	1
2.00	1	1	2	1
3.00	2	2	2	1
3.00	2	2	2	1
3.00	2	2	2	1
3.00	2	2	2	1
3.00	2	2	2	1
3.00	2	2	2	1
1.00	1	1	2	1
1.00	1	1	2	1
2.00	1	1	2	2
1.00	1	1	2	1
1.00	1	1	2	1
2.00	1	1	2	2
1.00	1	1	2	1
2.00	1	1	2	2
1.00	1	1	2	1
2.00	2	1	2	2
2.00	2	1	2	2
2.00	2	1	2	2
3.00	2	1	2	2
3.00	2	1	2	2
2.00	2	2	2	1
2.00	2	2	2	1
2.00	2	2	2	1
2.00	2	2	2	1
2.00	2	2	2	1
2.00	1	1	2	2
2.00	2	1	2	2
1.00	2	2	2	2
2.00	2	2	2	1
2.00	1	1	2	2
2.00	2	1	2	2
3.00	2	2	2	2
1.00	2	2	2	2
2.00	2	2	2	1

2.00	2	2	2	2
2.00	1	1	2	2
2.00	2	1	2	2
3.00	2	2	2	2
1.00	2	2	2	2
2.00	2	2	2	1
2.00	1	1	2	2
2.00	2	1	2	2
3.00	2	2	2	2
1.00	2	2	2	2
2.00	2	2	2	1
2.00	2	2	2	2
2.00	1	1	2	2
2.00	2	1	2	2
3.00	2	2	2	2
1.00	2	2	2	2
2.00	2	2	2	1
2.00	2	2	2	2
2.00	1	1	2	2
2.00	2	1	2	2
3.00	2	2	2	2
1.00	2	2	2	2

AniexBefPregnancy	DiabetBefPregnancy	EndocBefPregnancy	BirthProbBefPregnancy	DuratBetPregnancy
2	2	2	2	1
2	2	2	2	2
2	2	2	2	1
1	2	2	2	1
2	2	2	2	2
1	1	2	1	1
1	2	2	1	2
2	2	2	2	2
1	2	2	2	2
2	2	2	2	1
2	2	2	2	2
1	1	2	1	1
2	2	2	2	2
2	2	2	2	2
2	2	2	2	1
2	2	1	2	2
2	2	2	2	1
2	2	2	2	1
1	2	2	2	1
2	2	2	2	1
2	2	1	2	2
2	2	2	1	1
1	2	2	2	1
2	2	2	2	2
1	1	2	1	1
2	2	2	2	2
2	2	2	2	2
2	2	2	2	1
2	2	1	2	1
2	2	2	2	2
1	2	2	2	2
2	2	2	2	1
2	2	2	2	2
2	2	2	2	1
2	2	2	2	1
2	2	2	2	1
2	2	2	2	2
2	2	2	2	2
2	2	2	2	1
2	2	2	1	1
2	2	2	2	2
2	2	2	2	1
2	2	2	2	1
1	1	2	1	1
2	2	2	2	2
2	2	2	2	1
1	1	2	1	1
2	2	2	2	1
1	2	2	2	2
2	2	2	2	1
1	2	2	2	1

1	1	2	1	1
2	2	2	2	1
2	2	2	2	2
1	2	2	2	1
2	2	2	2	2
2	2	2	2	1
2	2	2	2	2
2	2	2	2	1
2	2	1	2	2
2	2	2	2	1
2	2	2	2	1
2	2	2	2	2
1	1	2	1	1
2	2	2	1	2
2	2	2	2	2
2	2	2	2	1
2	2	2	2	1
2	2	2	2	2
1	1	2	1	1
2	2	2	1	2
2	2	2	2	2
2	2	2	2	1
2	2	2	2	1
2	2	2	2	2
1	1	2	1	1
2	2	2	1	2
2	2	2	2	2
2	2	2	2	1
2	2	2	2	1
2	2	2	2	2
1	1	2	1	1
2	2	2	1	2
2	2	2	2	2
2	2	2	2	1
2	2	2	2	1
2	2	2	2	2
1	1	2	1	1
2	2	2	1	2
2	2	2	2	2
2	2	2	2	1
2	2	2	2	1
2	2	2	2	2
1	1	2	1	1
1	2	2	1	2
2	2	2	2	2
2	2	2	2	2
2	2	2	2	2
2	2	2	2	2
2	2	2	2	2
2	2	2	2	2
2	2	2	2	2
2	2	2	2	2

2	2	2	2	2
2	2	2	2	2
2	2	2	1	1
1	1	2	1	1
2	2	2	1	1
1	1	2	1	1
2	2	2	1	1
1	1	2	1	1
2	2	2	1	1
1	1	2	1	1
2	2	2	1	1
1	1	2	1	1
2	2	2	2	1
1	1	2	1	1
2	2	2	2	1
1	1	2	1	1
2	2	2	2	1
1	1	2	1	1
2	2	2	2	1
1	1	2	1	1
2	2	2	2	1
1	1	2	1	1
2	2	2	2	1
1	1	2	1	1
2	2	2	2	1
1	1	2	1	1
2	2	2	2	1
1	1	2	1	1
2	2	2	2	1
1	1	2	1	1
2	2	2	2	1
1	1	2	1	1
2	2	2	2	1
1	2	2	2	2
2	2	2	2	1
1	1	2	1	1
2	2	1	2	2
2	2	2	2	1
2	2	2	2	1
2	2	1	2	2
2	2	2	2	1
2	2	2	2	2
1	1	2	1	1
2	2	1	2	1
2	2	2	2	2
1	2	2	2	2
2	2	2	2	1
2	2	2	2	2
2	2	2	2	1
2	2	2	2	1
2	2	2	2	1
2	2	2	2	1

1	2	2	2	2
2	2	2	2	1
1	1	2	1	1
2	2	1	2	1
2	2	2	2	1
2	2	2	2	1
2	2	1	2	2
2	2	2	2	1
2	2	2	2	2
1	1	2	1	1
2	2	1	2	1
2	2	2	2	2
1	2	2	2	2
2	2	2	2	1
2	2	2	2	2
2	2	2	2	1
2	2	2	2	1
2	2	2	2	1
2	2	2	2	1
2	2	2	2	1
2	2	1	2	2
1	2	2	2	2
2	2	2	2	1
1	2	2	1	1
2	2	1	2	1
2	2	2	2	1
2	2	2	2	2
2	2	1	2	2
2	2	2	2	1
2	2	2	2	2
1	1	2	1	1
2	2	1	2	1
2	2	2	2	2
1	2	2	2	2
2	2	2	2	1
2	2	2	2	2
2	2	2	2	1
2	2	2	2	1
2	2	2	2	1
1	2	2	2	2
2	2	2	2	1
1	2	2	1	1
2	2	1	2	1
2	2	2	2	1
2	2	2	2	2
2	2	1	2	2
2	2	2	2	1
2	2	2	2	2
1	1	2	1	1
2	2	1	2	1
2	2	2	2	2
1	2	2	2	2

2	2	2	2	1
2	2	2	2	2
2	2	2	2	1
2	2	2	2	1
2	2	2	2	1
2	2	2	2	1
2	2	2	2	1
2	2	1	2	2
1	2	2	2	2
2	2	2	2	1
1	2	2	1	1
2	2	1	2	1
2	2	2	2	1
2	2	2	2	2
2	2	1	2	2
2	2	2	2	1
2	2	2	2	2
1	1	2	1	1
2	2	1	2	1
2	2	2	2	2
1	2	2	2	2
2	2	2	2	1
2	2	2	2	2
2	2	2	2	1
2	2	2	2	1
2	2	2	2	1
2	2	2	2	1
2	2	2	2	1
2	2	1	2	2
1	2	2	2	2
2	2	2	2	1
1	2	2	1	1
2	2	1	2	1
2	2	2	2	1
2	2	2	2	2
2	2	1	2	2
2	2	2	2	1
2	2	2	2	2
1	1	2	1	1
1	2	2	2	1
2	2	2	2	1
1	2	2	2	1
2	2	2	2	1
1	2	2	2	1
2	2	2	2	1
1	2	2	2	1
2	2	2	2	1
1	2	2	2	1
2	2	2	2	1
1	2	2	2	1
2	2	2	2	2
2	2	2	2	1

2	2	2	2	1
2	2	2	2	1
2	2	2	2	1
2	2	2	2	1
2	2	2	2	2
2	2	2	2	1
2	2	2	2	2
2	2	2	2	1
2	2	2	2	2
2	2	2	2	1
2	2	2	2	2
2	2	2	2	1
2	2	2	2	2
2	2	2	2	1
2	2	2	2	2
2	2	2	2	1
2	2	2	2	1
2	2	2	2	1
2	2	2	2	1
2	2	2	2	1
2	2	2	2	1
1	2	2	2	1
1	2	2	2	1
1	2	2	2	1
1	2	2	2	1
1	2	2	2	1
2	2	2	2	2
2	2	2	2	2
2	2	2	2	2
2	2	2	2	2
2	2	2	2	2
2	2	2	2	2
2	2	2	2	2
2	2	2	2	2
2	2	2	2	2
2	2	2	2	2
2	2	2	2	2
2	2	2	2	2
2	2	2	2	2
2	2	2	2	2
2	2	2	2	2
2	2	2	2	2
2	2	2	2	1
2	2	2	2	2
2	2	2	2	2
2	2	2	2	2
2	2	2	2	2
2	2	2	2	1
2	2	2	2	2
2	2	2	2	1
2	2	2	2	1
2	2	2	2	1

2	2	2	2	2
2	2	2	2	2
2	2	2	2	2
2	2	2	2	2
1	2	2	2	1
2	2	2	2	2
1	2	2	2	1
2	2	2	2	2
1	2	2	2	1
2	2	2	2	2
1	2	2	2	1
2	2	2	2	2
1	2	2	2	1
2	2	2	2	1
2	2	2	2	1
2	2	2	2	1
2	2	2	2	1
2	2	2	2	1
2	2	2	2	2
2	2	2	2	2
2	2	2	2	2
2	2	2	2	2
2	2	2	2	2
2	2	2	2	2
2	2	2	2	2
2	2	2	2	2
2	2	2	2	1
2	2	2	2	2
2	2	2	2	2
2	2	2	2	1
2	2	2	2	2
2	2	2	2	1
2	2	2	2	2
2	2	2	2	1
2	2	2	2	1
2	2	2	2	1
2	2	2	2	1
2	2	2	2	1
2	2	2	2	1
2	2	2	2	1
2	2	2	2	1
2	2	2	2	1
2	2	2	2	1
2	2	2	2	1
2	2	2	2	1
2	2	2	1	1
1	2	2	2	2
2	2	2	2	1
2	2	2	2	1
2	2	2	2	2
2	2	2	1	1
1	2	2	2	2

2	2	2	2	2
2	2	2	2	1
2	2	2	2	1
2	2	2	2	2
2	2	2	1	1
1	2	2	2	2
2	2	2	2	1
2	2	2	2	1
2	2	2	2	2
2	2	2	1	1
1	2	2	2	2
2	2	2	2	2
2	2	2	2	1
2	2	2	2	1
2	2	2	2	2
2	2	2	1	1
1	2	2	2	2
2	2	2	2	2
2	2	2	2	1
2	2	2	2	1
2	2	2	2	2
2	2	2	1	1

EmerPregnancy	PrevAbortion	SmokPregnancy	BadEventPregnancy	CommSupPregnanc	PassSmokPregnancy
2	2	2	1	2	1
2	2	2	1	2	1
2	2	2	2	2	1
2	2	2	2	1	2
2	2	2	2	1	2
2	2	2	1	2	1
2	1	2	1	2	1
2	2	2	2	2	2
2	2	2	1	1	1
2	2	1	1	1	1
2	2	2	2	1	2
2	2	1	2	2	1
1	1	2	1	2	2
2	2	1	2	1	2
2	2	1	1	2	1
1	2	1	1	2	1
1	1	1	2	1	1
2	2	2	1	2	1
2	2	2	2	1	1
2	2	1	2	1	1
1	2	1	1	2	1
2	2	2	2	1	1
2	2	2	1	2	1
1	1	1	1	2	1
2	2	2	1	1	2
2	1	1	1	2	1
2	2	2	1	2	2
2	2	2	2	1	2
1	2	1	2	1	2
2	2	2	2	1	2
2	2	2	2	2	1
1	1	1	1	1	1
2	2	2	2	2	1
2	2	2	2	2	2
2	2	1	1	2	1
1	1	2	2	2	1
2	1	2	2	1	2
2	2	2	2	1	2
2	2	1	2	1	1
2	2	1	1	1	2
2	2	2	2	2	2
2	2	1	1	3	1
2	2	2	1	2	2
2	2	1	2	2	1
2	2	2	2	2	1
2	2	2	2	2	1
2	2	1	2	2	2
2	2	1	2	2	1
2	2	2	2	1	2
2	1	1	1	2	1
2	2	2	2	1	1

2	2	2	2	2	1
2	2	2	1	1	2
2	2	2	1	2	2
2	2	1	2	2	2
2	2	2	1	2	1
2	2	2	1	2	1
2	2	1	1	2	1
2	2	2	2	2	2
2	2	2	2	1	2
2	2	1	1	2	2
2	2	1	1	2	1
2	2	2	1	2	1
2	2	2	1	2	1
2	1	2	1	2	1
2	2	2	2	2	2
2	2	2	1	2	2
2	2	2	2	2	1
2	2	2	1	2	1
2	2	2	1	2	1
2	1	2	1	2	1
2	2	2	2	2	2
2	2	2	1	2	2
2	2	2	2	2	1
2	2	2	1	2	1
2	2	2	1	2	1
2	1	2	1	2	1
2	2	2	2	2	2
2	2	2	1	2	2
2	2	2	2	2	1
2	2	2	1	2	1
2	2	2	1	2	1
2	1	2	1	2	1
2	2	2	2	2	2
2	2	2	1	2	2
2	2	2	2	2	1
2	2	2	1	2	1
2	2	2	1	2	1
2	1	2	1	2	1
2	2	2	2	2	2
2	2	2	1	2	2
2	2	2	2	2	1
2	2	2	1	2	1
2	2	2	1	2	1
2	1	2	1	2	1
2	2	2	2	2	1
2	2	2	1	2	2
2	2	2	2	2	1
2	2	2	1	2	2
2	2	2	2	2	1
2	2	2	1	2	2
2	2	2	2	2	1
2	2	2	1	2	2

2	2	2	2	2	1
2	2	2	1	2	2
2	2	1	1	1	2
2	2	2	2	2	1
2	2	1	1	1	2
2	2	2	2	2	1
2	2	1	1	1	2
2	2	2	2	2	1
2	2	1	1	1	2
2	2	2	2	2	1
2	2	1	1	1	2
2	2	2	2	2	1
2	2	1	2	1	1
2	2	1	2	2	2
2	2	1	2	1	1
2	2	1	2	2	2
2	2	1	2	1	1
2	2	1	2	2	2
2	2	1	2	1	1
2	2	1	2	2	2
2	2	1	2	1	1
2	2	1	2	2	2
2	2	1	2	1	1
2	2	1	2	2	1
2	2	1	2	1	1
2	2	1	2	2	1
2	2	1	2	1	1
2	2	1	2	2	1
2	2	1	2	1	1
2	2	1	2	2	1
2	2	1	2	1	1
2	2	1	2	2	1
2	2	1	2	1	1
2	2	2	1	1	1
2	2	1	1	1	1
2	2	1	2	2	1
1	2	1	1	2	1
2	2	2	1	2	1
2	2	2	2	1	1
1	2	1	1	2	1
2	2	2	1	2	1
1	1	1	1	2	1
2	2	2	1	1	2
1	2	1	2	1	1
2	2	2	2	1	2
2	2	2	2	2	1
1	1	1	1	1	1
2	2	2	2	2	1
1	1	2	2	2	1
2	1	1	1	2	1
2	2	2	1	1	2
2	2	2	1	2	1

2	2	2	1	1	1
2	2	1	1	1	1
2	2	1	2	2	1
1	2	1	1	2	1
2	2	2	1	2	1
2	2	2	2	1	1
1	2	1	1	2	1
2	2	2	1	2	1
1	1	1	1	2	1
2	2	2	1	1	2
1	2	1	2	1	1
2	2	2	2	1	2
2	2	2	2	2	1
1	1	1	1	1	1
2	2	2	2	2	1
1	1	2	2	2	1
2	1	1	1	2	1
2	2	2	1	1	2
2	2	2	1	2	1
2	2	2	2	2	2
2	2	2	2	1	2
2	2	2	1	1	1
2	2	1	1	1	1
2	2	1	2	2	1
1	2	1	1	2	1
2	2	2	1	2	1
2	2	2	2	1	1
1	2	1	1	2	1
2	2	2	1	2	1
1	1	1	1	2	1
2	2	2	1	1	2
1	2	1	2	1	1
2	2	2	2	1	2
2	2	2	2	2	1
1	1	1	1	1	1
2	2	2	2	2	1
1	1	2	2	2	1
2	1	1	1	2	1
2	2	2	1	1	2
2	2	2	1	1	1
2	2	1	1	1	1
2	2	1	2	2	1
1	2	1	1	2	1
2	2	2	1	2	1
2	2	2	2	1	1
1	2	1	1	2	1
2	2	2	1	2	1
1	1	1	1	2	1
2	2	2	1	1	2
1	2	1	2	1	1
2	2	2	2	1	2
2	2	2	2	2	1

1	1	1	1	1	1
2	2	2	2	2	1
1	1	2	2	2	1
2	1	1	1	2	1
2	2	2	1	1	2
2	2	2	1	2	1
2	2	2	2	2	2
2	2	2	2	1	2
2	2	2	1	1	1
2	2	1	1	1	1
2	2	1	2	2	1
1	2	1	1	2	1
2	2	2	1	2	1
2	2	2	2	1	1
1	2	1	1	2	1
2	2	2	1	2	1
1	1	1	1	2	1
2	2	2	1	1	2
1	2	1	2	1	1
2	2	2	2	1	2
2	2	2	2	2	1
1	1	1	1	1	1
2	2	2	2	2	1
1	1	2	2	2	1
2	1	1	1	2	1
2	2	2	1	1	2
2	2	2	1	2	1
2	2	2	2	2	2
2	2	2	2	1	2
2	2	2	2	1	1
2	2	1	1	1	1
2	2	1	2	2	1
1	2	1	1	2	1
2	2	2	1	2	1
2	2	2	2	1	1
1	2	1	1	2	1
2	2	2	1	2	1
1	1	1	1	2	1
2	2	2	1	1	2
2	2	2	2	1	2
2	2	1	1	2	1
2	2	2	2	1	2
2	2	1	1	2	1
2	2	2	2	1	2
2	2	1	1	2	1
2	2	2	2	1	2
2	2	1	1	2	1
2	2	2	2	1	2
2	2	1	1	2	1
2	2	2	2	1	2
2	2	2	2	1	2
2	2	2	2	1	2

2	2	2	2	1	2
2	2	2	2	1	2
2	2	2	2	1	2
2	2	2	2	1	2
2	2	2	2	2	2
1	1	1	2	1	1
2	2	2	2	2	2
1	1	1	2	1	1
2	2	2	2	2	2
1	1	1	2	1	1
2	2	2	2	2	2
1	1	1	2	1	1
2	2	2	2	2	2
1	1	1	2	1	1
2	2	2	2	2	2
1	1	1	2	1	1
2	2	1	2	2	1
2	2	1	2	2	1
2	2	1	2	2	1
2	2	1	2	2	1
2	2	1	2	2	1
2	2	2	2	1	1
2	2	2	2	1	1
2	2	2	2	1	1
2	2	2	2	1	1
2	2	2	2	1	1
1	1	2	1	2	2
2	2	2	1	2	2
2	1	2	2	1	2
2	2	2	1	2	1
1	1	2	1	2	2
2	2	2	1	2	2
2	1	2	2	1	2
2	2	2	1	2	1
1	1	2	1	2	2
2	2	2	1	2	2
2	1	2	2	1	2
2	2	2	1	2	1
1	1	2	1	2	2
2	2	2	1	2	2
2	1	2	2	1	2
2	2	2	1	2	1
2	2	1	1	2	1
1	1	2	1	2	2
2	2	2	1	2	2
2	1	2	2	1	2
2	2	2	1	2	1
2	2	1	1	2	1
1	1	2	1	2	2
2	2	1	1	2	1
2	2	1	1	2	1
2	2	1	1	2	1

2	2	1	1	2	1
2	2	1	1	2	1
2	2	1	1	2	1
2	2	2	2	1	2
2	2	1	2	2	2
2	2	2	2	1	2
2	2	1	2	2	2
2	2	2	2	1	2
2	2	1	2	2	2
2	2	2	2	1	2
2	2	1	2	2	2
2	2	2	2	1	2
2	2	1	2	2	2
2	2	1	1	3	1
2	2	1	1	3	1
2	2	1	1	3	1
2	2	1	1	3	1
2	2	1	1	3	1
2	2	1	2	1	2
2	2	1	2	1	2
2	2	1	2	1	2
2	2	1	2	1	2
2	2	1	2	1	2
2	2	1	2	1	2
2	1	1	1	2	1
2	1	1	1	2	1
2	2	1	1	2	2
2	1	1	1	2	1
2	1	1	1	2	1
2	2	1	1	2	2
2	1	1	1	2	1
2	2	1	1	2	2
2	1	1	1	2	1
2	2	2	2	2	2
2	2	2	2	2	2
2	2	2	2	2	2
2	2	2	2	2	2
2	2	2	2	2	2
2	2	2	2	1	2
2	2	2	2	1	2
2	2	2	2	1	2
2	2	2	2	1	2
2	2	2	2	1	2
2	2	2	1	2	1
2	2	2	2	2	1
2	2	2	2	1	1
2	2	2	2	1	2
2	2	2	1	2	1
2	2	2	2	2	1
2	2	2	2	1	2
2	2	2	2	1	1
2	2	2	2	1	2

2	2	1	1	2	1
2	2	2	1	2	1
2	2	2	2	2	1
2	2	2	2	1	2
2	2	2	2	1	1
2	2	2	2	1	2
2	2	2	1	2	1
2	2	2	2	2	1
2	2	2	2	1	2
2	2	2	2	1	1
2	2	2	2	1	2
2	2	1	1	2	1
2	2	2	1	2	1
2	2	2	2	2	1
2	2	2	2	1	2
2	2	2	2	1	1
2	2	2	2	1	2
2	2	1	1	2	1
2	2	2	1	2	1
2	2	2	2	2	1
2	2	2	2	1	2
2	2	2	2	1	1

PlanPregnancy	HEIGHT_C	WEIGHT_BEF(	WEIGHNORMALABN(	HBA1Cx	HBA1Clevel
1	1.00	1.00	2.00	5.0	Normal
2	2.00	2.00	2.00	4.8	Normal
2	2.00	1.00	2.00	5.2	Normal
1	2.00	2.00	2.00	6.9	abnorma;
1	2.00	1.00	2.00	5.3	Normal
2	2.00	2.00	2.00	8.7	abnorma;
1	2.00	2.00	2.00	7.7	abnorma;
1	2.00	1.00	2.00	5.3	Normal
1	2.00	2.00	2.00	7.4	abnorma;
2	2.00	2.00	2.00	5.1	Normal
1	2.00	1.00	2.00	5.3	Normal
1	2.00	2.00	2.00	8.1	abnorma;
1	2.00	1.00	2.00	5.3	Normal
1	2.00	2.00	2.00	5.3	Normal
1	1.00	1.00	2.00	5.1	Normal
2	1.00	1.00	1.00	4.5	Normal
2	2.00	2.00	2.00	9.0	abnorma;
2	2.00	2.00	2.00	5.2	Normal
2	2.00	2.00	2.00	6.9	abnorma;
2	2.00	2.00	2.00	6.9	abnorma;
2	1.00	1.00	1.00	4.7	Normal
1	2.00	3.00	2.00	9.0	abnorma;
2	2.00	2.00	2.00	6.9	abnorma;
2	1.00	1.00	1.00	4.3	Normal
1	2.00	2.00	2.00	8.4	abnorma;
2	2.00	2.00	2.00	5.1	Normal
2	2.00	1.00	2.00	5.3	Normal
1	2.00	2.00	2.00	5.1	Normal
2	2.00	1.00	2.00	5.2	Normal
1	2.00	2.00	2.00	5.1	Normal
2	2.00	2.00	2.00	6.9	abnorma;
2	1.00	1.00	2.00	4.8	Normal
2	2.00	1.00	2.00	5.4	Normal
2	2.00	2.00	2.00	6.1	abnorma;
2	2.00	1.00	2.00	5.2	Normal
2	2.00	1.00	1.00	4.7	Normal
1	2.00	1.00	2.00	5.3	Normal
1	2.00	2.00	2.00	6.4	abnorma;
1	1.00	1.00	2.00	4.9	Normal
2	2.00	2.00	2.00	7.7	abnorma;
1	2.00	2.00	2.00	4.9	Normal
2	2.00	2.00	2.00	5.4	Normal
1	2.00	2.00	2.00	4.5	Normal
1	2.00	2.00	2.00	8.5	abnorma;
2	2.00	1.00	2.00	5.5	Normal
2	1.00	1.00	2.00	5.2	Normal
1	2.00	2.00	2.00	8.4	abnorma;
1	1.00	1.00	1.00	4.7	Normal
2	2.00	2.00	2.00	7.1	abnorma;
2	1.00	1.00	2.00	5.2	Normal
1	2.00	2.00	2.00	7.2	abnorma;

2	2.00	2.00	2.00	8.5	abnorma;
2	2.00	1.00	2.00	5.3	Normal
2	2.00	1.00	2.00	5.5	Normal
1	2.00	2.00	2.00	7.5	abnorma;
1	1.00	1.00	2.00	5.0	Normal
2	1.00	1.00	2.00	4.9	Normal
2	2.00	1.00	2.00	5.2	Normal
2	2.00	1.00	2.00	5.5	Normal
1	2.00	2.00	2.00	5.2	Normal
2	2.00	1.00	2.00	5.3	Normal
1	1.00	1.00	2.00	5.0	Normal
2	2.00	2.00	2.00	6.3	Normal
2	2.00	2.00	2.00	9.0	abnorma;
1	2.00	2.00	2.00	7.4	abnorma;
1	2.00	2.00	2.00	5.2	Normal
1	2.00	2.00	2.00	4.9	Normal
2	1.00	1.00	2.00	5.1	Normal
2	2.00	2.00	2.00	5.5	Normal
2	2.00	2.00	2.00	8.7	abnorma;
1	2.00	2.00	2.00	7.5	abnorma;
1	2.00	2.00	2.00	5.3	Normal
1	2.00	2.00	2.00	4.9	Normal
2	1.00	1.00	2.00	5.1	Normal
2	2.00	2.00	2.00	5.5	Normal
2	2.00	2.00	2.00	9.0	abnorma;
1	2.00	2.00	2.00	7.5	abnorma;
1	2.00	2.00	2.00	5.2	Normal
1	2.00	2.00	2.00	5.2	Normal
2	1.00	1.00	2.00	5.2	Normal
2	2.00	2.00	2.00	4.9	Normal
2	2.00	2.00	2.00	9.0	abnorma;
1	2.00	2.00	2.00	7.5	abnorma;
1	2.00	2.00	2.00	5.2	Normal
1	2.00	2.00	2.00	5.2	Normal
2	1.00	1.00	2.00	5.1	Normal
2	2.00	2.00	2.00	4.8	Normal
2	2.00	2.00	2.00	9.0	abnorma;
1	2.00	2.00	2.00	7.5	abnorma;
1	2.00	2.00	2.00	5.2	Normal
1	2.00	2.00	2.00	5.2	Normal
2	1.00	1.00	2.00	5.2	Normal
2	2.00	2.00	2.00	5.1	Normal
2	2.00	2.00	2.00	9.0	abnorma;
1	2.00	2.00	2.00	7.4	abnorma;
2	2.00	1.00	2.00	5.5	Normal
2	2.00	1.00	2.00	5.5	Normal
2	2.00	1.00	2.00	5.5	Normal
2	2.00	1.00	2.00	5.5	Normal
2	2.00	1.00	2.00	5.3	Normal
2	2.00	1.00	2.00	5.5	Normal
2	2.00	1.00	2.00	5.5	Normal
2	2.00	1.00	2.00	5.5	Normal

2	2.00	1.00	2.00	5.5	Normal
2	2.00	1.00	2.00	5.5	Normal
2	2.00	2.00	2.00	8.1	abnorma;
2	2.00	2.00	2.00	8.5	abnorma;
2	2.00	2.00	2.00	8.1	abnorma;
2	2.00	2.00	2.00	8.5	abnorma;
2	2.00	2.00	2.00	8.1	abnorma;
2	2.00	2.00	2.00	8.5	abnorma;
2	2.00	2.00	2.00	8.1	abnorma;
2	2.00	2.00	2.00	8.7	abnorma;
2	2.00	2.00	2.00	8.1	abnorma;
2	2.00	2.00	2.00	8.4	abnorma;
1	1.00	1.00	2.00	4.9	Normal
1	2.00	2.00	2.00	8.4	abnorma;
1	1.00	1.00	2.00	4.9	Normal
1	2.00	2.00	2.00	8.4	abnorma;
1	1.00	1.00	2.00	4.9	Normal
1	2.00	2.00	2.00	8.4	abnorma;
1	1.00	1.00	2.00	5.0	Normal
1	2.00	2.00	2.00	8.4	abnorma;
1	1.00	1.00	2.00	5.0	Normal
1	2.00	2.00	2.00	8.5	abnorma;
2	2.00	2.00	2.00	6.9	abnorma;
1	2.00	2.00	2.00	8.5	abnorma;
2	2.00	2.00	2.00	6.9	abnorma;
1	2.00	2.00	2.00	8.5	abnorma;
2	2.00	2.00	2.00	6.9	abnorma;
1	2.00	2.00	2.00	8.5	abnorma;
2	2.00	2.00	2.00	6.9	abnorma;
1	2.00	2.00	2.00	8.5	abnorma;
2	2.00	2.00	2.00	6.9	abnorma;
1	2.00	2.00	2.00	8.5	abnorma;
2	2.00	2.00	2.00	6.9	abnorma;
1	2.00	2.00	2.00	7.3	abnorma;
2	2.00	2.00	2.00	5.2	Normal
1	2.00	2.00	2.00	8.2	abnorma;
2	1.00	1.00	1.00	4.5	Normal
2	2.00	2.00	2.00	4.8	Normal
2	2.00	2.00	2.00	6.9	abnorma;
2	1.00	1.00	1.00	4.7	Normal
2	2.00	2.00	2.00	6.9	abnorma;
2	1.00	1.00	1.00	4.3	Normal
1	2.00	2.00	2.00	8.4	abnorma;
2	2.00	2.00	2.00	5.2	Normal
1	2.00	2.00	2.00	5.5	Normal
2	2.00	2.00	2.00	7.1	abnorma;
2	1.00	1.00	2.00	4.8	Normal
2	2.00	1.00	2.00	5.5	Normal
2	2.00	1.00	1.00	4.7	Normal
2	1.00	1.00	2.00	5.2	Normal
2	2.00	1.00	2.00	5.2	Normal
2	1.00	1.00	2.00	4.9	Normal

1	2.00	2.00	2.00	7.3	abnorma;
2	2.00	2.00	2.00	5.3	Normal
1	2.00	2.00	2.00	8.2	abnorma;
2	1.00	1.00	1.00	4.5	Normal
2	2.00	2.00	2.00	4.5	Normal
2	2.00	2.00	2.00	6.9	abnorma;
2	1.00	1.00	1.00	4.7	Normal
2	2.00	2.00	2.00	6.9	abnorma;
2	1.00	1.00	1.00	4.3	Normal
1	2.00	2.00	2.00	8.4	abnorma;
2	2.00	1.00	2.00	5.1	Normal
1	2.00	2.00	2.00	5.2	Normal
2	2.00	2.00	2.00	6.9	abnorma;
2	1.00	1.00	2.00	4.8	Normal
2	2.00	1.00	2.00	5.5	Normal
2	2.00	1.00	1.00	4.7	Normal
2	1.00	1.00	2.00	5.2	Normal
2	2.00	1.00	2.00	5.2	Normal
2	1.00	1.00	2.00	4.9	Normal
2	2.00	1.00	2.00	5.5	Normal
1	2.00	2.00	2.00	6.2	abnorma;
1	2.00	2.00	2.00	7.3	abnorma;
2	2.00	2.00	2.00	5.3	Normal
1	2.00	2.00	2.00	8.2	abnorma;
2	1.00	1.00	1.00	4.5	Normal
2	2.00	2.00	2.00	4.8	Normal
2	2.00	2.00	2.00	6.9	abnorma;
2	1.00	1.00	1.00	4.7	Normal
2	2.00	2.00	2.00	6.9	abnorma;
2	1.00	1.00	1.00	4.3	Normal
1	2.00	2.00	2.00	8.4	abnorma;
2	2.00	2.00	2.00	5.2	Normal
1	2.00	2.00	2.00	5.1	Normal
2	2.00	2.00	2.00	7.1	abnorma;
2	1.00	1.00	1.00	4.8	Normal
2	2.00	1.00	2.00	5.5	Normal
2	2.00	1.00	1.00	4.7	Normal
2	1.00	1.00	2.00	5.2	Normal
2	2.00	1.00	2.00	5.2	Normal
1	2.00	2.00	2.00	7.3	abnorma;
2	2.00	2.00	2.00	5.3	Normal
1	2.00	2.00	2.00	8.2	abnorma;
2	1.00	1.00	1.00	4.5	Normal
2	2.00	2.00	2.00	4.8	Normal
2	2.00	2.00	2.00	6.9	abnorma;
2	1.00	1.00	1.00	4.7	Normal
2	2.00	2.00	2.00	6.9	abnorma;
2	1.00	1.00	1.00	4.3	Normal
1	2.00	2.00	2.00	8.4	abnorma;
2	2.00	2.00	2.00	5.3	Normal
1	2.00	2.00	2.00	5.1	Normal
2	2.00	2.00	2.00	7.1	abnorma;

2	1.00	1.00	2.00	4.9	Normal
2	2.00	1.00	2.00	5.5	Normal
2	2.00	1.00	1.00	4.7	Normal
2	1.00	1.00	2.00	5.2	Normal
2	2.00	1.00	2.00	5.2	Normal
2	1.00	1.00	2.00	4.9	Normal
2	2.00	1.00	2.00	5.5	Normal
1	2.00	2.00	2.00	6.0	abnorma;
1	2.00	2.00	2.00	7.4	abnorma;
2	2.00	2.00	2.00	5.2	Normal
1	2.00	2.00	2.00	8.2	abnorma;
2	1.00	1.00	1.00	4.7	Normal
2	2.00	2.00	2.00	6.0	Normal
2	2.00	2.00	2.00	6.9	abnorma;
2	1.00	1.00	1.00	4.7	Normal
2	2.00	2.00	2.00	6.9	abnorma;
2	1.00	1.00	1.00	4.3	Normal
1	2.00	2.00	2.00	8.4	abnorma;
2	2.00	2.00	2.00	5.3	Normal
1	2.00	2.00	2.00	5.1	Normal
2	2.00	2.00	2.00	7.1	abnorma;
2	1.00	1.00	2.00	4.9	Normal
2	2.00	1.00	2.00	5.5	Normal
2	2.00	1.00	1.00	4.7	Normal
2	1.00	1.00	2.00	5.2	Normal
2	2.00	1.00	2.00	5.2	Normal
2	1.00	1.00	2.00	4.9	Normal
2	2.00	1.00	2.00	5.5	Normal
1	2.00	2.00	2.00	6.1	abnorma;
1	2.00	2.00	2.00	7.3	abnorma;
2	2.00	2.00	2.00	5.3	Normal
1	2.00	2.00	2.00	8.2	abnorma;
2	1.00	1.00	1.00	4.7	Normal
2	2.00	2.00	2.00	4.6	Normal
2	2.00	2.00	2.00	6.9	abnorma;
2	1.00	1.00	1.00	4.7	Normal
2	2.00	2.00	2.00	6.1	abnorma;
2	1.00	1.00	1.00	4.5	Normal
1	2.00	2.00	2.00	8.4	abnorma;
1	2.00	2.00	2.00	6.9	abnorma;
2	2.00	1.00	2.00	5.2	Normal
1	2.00	2.00	2.00	6.9	abnorma;
2	2.00	1.00	2.00	5.2	Normal
1	2.00	2.00	2.00	6.9	abnorma;
2	2.00	1.00	2.00	5.2	Normal
1	2.00	2.00	2.00	6.9	abnorma;
2	2.00	1.00	2.00	5.2	Normal
1	2.00	2.00	2.00	6.9	abnorma;
2	2.00	1.00	2.00	5.2	Normal
1	2.00	2.00	2.00	6.9	abnorma;
1	2.00	1.00	2.00	5.3	Normal
1	2.00	1.00	2.00	5.3	Normal

1	2.00	1.00	2.00	5.3	Normal
1	2.00	1.00	2.00	5.3	Normal
1	2.00	1.00	2.00	5.3	Normal
1	2.00	1.00	2.00	5.3	Normal
1	2.00	1.00	2.00	5.3	Normal
2	2.00	2.00	2.00	9.0	abnorma;
1	2.00	1.00	2.00	5.3	Normal
2	2.00	2.00	2.00	9.0	abnorma;
1	2.00	1.00	2.00	5.3	Normal
2	2.00	2.00	2.00	9.0	abnorma;
1	2.00	1.00	2.00	5.3	Normal
2	2.00	2.00	2.00	9.0	abnorma;
1	2.00	1.00	2.00	5.3	Normal
2	2.00	2.00	2.00	9.0	abnorma;
1	2.00	1.00	2.00	5.3	Normal
2	2.00	2.00	2.00	9.0	abnorma;
1	1.00	1.00	1.00	4.7	Normal
1	1.00	1.00	1.00	4.7	Normal
1	1.00	1.00	1.00	4.7	Normal
1	1.00	1.00	1.00	4.8	Normal
1	1.00	1.00	1.00	4.8	Normal
1	2.00	2.00	2.00	7.2	abnorma;
1	2.00	2.00	2.00	7.2	abnorma;
1	2.00	2.00	2.00	7.2	abnorma;
1	2.00	2.00	2.00	7.3	abnorma;
1	2.00	2.00	2.00	7.3	abnorma;
1	2.00	1.00	2.00	5.4	Normal
2	2.00	1.00	2.00	5.3	Normal
1	2.00	1.00	2.00	5.3	Normal
1	1.00	1.00	2.00	5.1	Normal
1	2.00	1.00	2.00	5.4	Normal
2	2.00	1.00	2.00	5.3	Normal
1	2.00	1.00	2.00	5.3	Normal
1	1.00	1.00	2.00	5.0	Normal
1	2.00	1.00	2.00	5.4	Normal
2	2.00	1.00	2.00	5.3	Normal
1	2.00	1.00	2.00	5.3	Normal
1	1.00	1.00	2.00	5.1	Normal
1	2.00	1.00	2.00	5.4	Normal
2	2.00	1.00	2.00	5.3	Normal
1	2.00	1.00	2.00	5.3	Normal
1	1.00	1.00	2.00	5.0	Normal
1	1.00	1.00	1.00	4.9	Normal
1	2.00	1.00	2.00	5.4	Normal
2	2.00	1.00	2.00	5.3	Normal
1	2.00	1.00	2.00	4.9	Normal
1	1.00	1.00	2.00	5.1	Normal
1	1.00	1.00	1.00	4.9	Normal
1	2.00	1.00	2.00	5.4	Normal
1	1.00	1.00	2.00	5.1	Normal
1	1.00	1.00	2.00	5.1	Normal
1	1.00	1.00	2.00	5.1	Normal

1	1.00	1.00	2.00	5.1	Normal
1	1.00	1.00	2.00	5.1	Normal
1	1.00	1.00	2.00	5.1	Normal
1	2.00	2.00	2.00	6.4	abnorma;
1	2.00	2.00	2.00	7.5	abnorma;
1	2.00	2.00	2.00	6.3	abnorma;
1	2.00	2.00	2.00	7.7	abnorma;
1	2.00	2.00	2.00	6.9	abnorma;
1	2.00	2.00	2.00	7.7	abnorma;
1	2.00	2.00	2.00	6.9	abnorma;
1	2.00	2.00	2.00	7.7	abnorma;
1	2.00	2.00	2.00	6.9	abnorma;
1	2.00	2.00	2.00	7.7	abnorma;
2	2.00	2.00	2.00	5.7	Normal
2	2.00	2.00	2.00	5.1	Normal
2	2.00	2.00	2.00	5.1	Normal
2	2.00	2.00	2.00	5.1	Normal
2	2.00	2.00	2.00	6.1	Normal
1	2.00	2.00	2.00	5.2	Normal
1	2.00	2.00	2.00	5.1	Normal
1	2.00	2.00	2.00	5.1	Normal
1	2.00	2.00	2.00	5.1	Normal
1	2.00	2.00	2.00	5.4	Normal
1	2.00	2.00	2.00	5.4	Normal
2	2.00	2.00	2.00	5.2	Normal
2	2.00	2.00	2.00	5.1	Normal
2	2.00	1.00	2.00	5.3	Normal
2	2.00	2.00	2.00	5.7	Normal
2	2.00	2.00	2.00	5.5	Normal
2	2.00	1.00	2.00	5.2	Normal
2	2.00	2.00	2.00	5.5	Normal
2	2.00	1.00	2.00	5.2	Normal
2	1.00	1.00	2.00	5.6	Normal
2	2.00	2.00	2.00	6.0	abnorma;
2	2.00	2.00	2.00	6.1	abnorma;
2	2.00	2.00	2.00	5.1	abnorma;
2	2.00	2.00	2.00	6.4	abnorma;
2	2.00	2.00	2.00	6.7	abnorma;
1	2.00	2.00	2.00	5.1	Normal
1	2.00	2.00	2.00	5.2	Normal
1	2.00	2.00	2.00	5.2	Normal
1	2.00	2.00	2.00	5.3	Normal
1	2.00	2.00	2.00	5.3	Normal
1	1.00	1.00	2.00	5.0	Normal
2	2.00	1.00	2.00	5.2	Normal
1	2.00	3.00	2.00	9.0	abnorma;
2	2.00	2.00	2.00	7.1	abnorma;
1	1.00	1.00	2.00	5.0	Normal
2	2.00	1.00	2.00	5.2	Normal
1	2.00	1.00	2.00	5.3	Normal
1	2.00	3.00	2.00	9.0	abnorma;
2	2.00	2.00	2.00	7.2	abnorma;

2	2.00	1.00	2.00	5.2	Normal
1	1.00	1.00	2.00	5.0	Normal
2	2.00	1.00	2.00	5.3	Normal
1	2.00	1.00	2.00	5.3	Normal
1	2.00	2.00	2.00	9.0	abnorma;
2	2.00	2.00	2.00	7.2	abnorma;
1	1.00	1.00	2.00	5.0	Normal
2	2.00	1.00	2.00	5.3	Normal
1	2.00	1.00	2.00	5.3	Normal
1	2.00	2.00	2.00	9.0	abnorma;
2	2.00	2.00	2.00	6.9	abnorma;
2	2.00	1.00	2.00	5.2	Normal
1	1.00	1.00	2.00	5.0	Normal
2	2.00	1.00	2.00	5.3	Normal
1	2.00	1.00	2.00	5.3	Normal
1	2.00	2.00	2.00	9.0	abnorma;
2	2.00	2.00	2.00	6.9	abnorma;
2	2.00	1.00	2.00	5.2	Normal
1	1.00	1.00	2.00	5.0	Normal
2	2.00	1.00	2.00	5.3	Normal
1	2.00	1.00	2.00	5.3	Normal
1	2.00	2.00	2.00	9.0	abnorma;

Fasting_GLUCOSE_LEVE	Fasting_GLUCOSE_LEVEL	WEIGHT_CURREN	STRESS_M2	DEPRESS_M
80	Normal	2.00	2.00	2.00
125	Normal	2.00	1.00	2.00
82	Normal	2.00	2.00	2.00
130	abnorma;	2.00	2.00	2.00
85	Normal	2.00	2.00	2.00
240	abnorma;	3.00	2.00	2.00
220	abnorma;	2.00	2.00	2.00
87	Normal	2.00	2.00	2.00
200	abnorma;	2.00	2.00	2.00
115	Normal	2.00	2.00	2.00
90	Normal	2.00	2.00	2.00
220	abnorma;	2.00	2.00	2.00
90	Normal	2.00	2.00	2.00
115	Normal	2.00	2.00	2.00
77	Normal	2.00	2.00	2.00
65	Normal	1.00	2.00	2.00
270	abnorma;	3.00	2.00	2.00
102	Normal	2.00	2.00	1.00
135	abnorma;	2.00	2.00	2.00
175	abnorma;	2.00	2.00	2.00
70	Normal	1.00	2.00	2.00
270	abnorma;	3.00	2.00	2.00
121	Normal	2.00	2.00	2.00
60	Normal	1.00	2.00	2.00
230	abnorma;	3.00	2.00	2.00
90	Normal	2.00	2.00	2.00
85	Normal	2.00	2.00	2.00
115	Normal	2.00	2.00	2.00
90	Normal	2.00	2.00	2.00
115	Normal	2.00	2.00	2.00
180	abnorma;	2.00	2.00	2.00
70	Normal	1.00	1.00	2.00
85	Normal	2.00	2.00	2.00
121	Normal	2.00	2.00	2.00
80	Normal	2.00	2.00	2.00
70	Normal	1.00	2.00	2.00
90	Normal	2.00	2.00	2.00
121	Normal	2.00	2.00	2.00
75	Normal	1.00	2.00	2.00
220	abnorma;	2.00	2.00	2.00
107	Normal	2.00	2.00	2.00
100	Normal	2.00	2.00	2.00
103	Normal	2.00	2.00	2.00
230	abnorma;	3.00	2.00	2.00
90	Normal	2.00	2.00	2.00
77	Normal	2.00	2.00	2.00
240	abnorma;	3.00	2.00	2.00
75	Normal	1.00	2.00	2.00
176	abnorma;	2.00	2.00	2.00
75	Normal	2.00	2.00	2.00
195	abnorma;	2.00	2.00	1.00

230	abnorma;	3.00	2.00	2.00
85	Normal	2.00	2.00	2.00
85	Normal	2.00	2.00	2.00
210	abnorma;	2.00	2.00	2.00
80	Normal	2.00	2.00	2.00
70	Normal	1.00	2.00	2.00
80	Normal	2.00	2.00	2.00
85	Normal	2.00	2.00	2.00
125	Normal	2.00	2.00	2.00
83	Normal	2.00	2.00	2.00
75	Normal	1.00	2.00	2.00
120	Normal	2.00	1.00	2.00
240	abnorma;	3.00	2.00	2.00
210	abnorma;	2.00	2.00	1.00
115	Normal	2.00	2.00	2.00
107	Normal	2.00	2.00	2.00
77	Normal	2.00	2.00	2.00
120	Normal	2.00	1.00	2.00
240	abnorma;	3.00	2.00	2.00
220	abnorma;	2.00	2.00	1.00
115	Normal	2.00	2.00	2.00
107	Normal	2.00	2.00	2.00
77	Normal	2.00	2.00	2.00
120	Normal	2.00	1.00	2.00
240	abnorma;	3.00	2.00	2.00
210	abnorma;	2.00	2.00	2.00
115	Normal	2.00	2.00	2.00
107	Normal	2.00	2.00	2.00
77	Normal	2.00	2.00	2.00
120	Normal	2.00	1.00	2.00
240	abnorma;	3.00	2.00	2.00
210	abnorma;	2.00	2.00	1.00
115	Normal	2.00	2.00	2.00
107	Normal	2.00	2.00	2.00
77	Normal	2.00	2.00	2.00
120	Normal	2.00	1.00	2.00
240	abnorma;	3.00	2.00	2.00
220	abnorma;	2.00	2.00	1.00
115	Normal	2.00	2.00	2.00
107	Normal	2.00	2.00	2.00
77	Normal	2.00	2.00	2.00
120	Normal	2.00	1.00	2.00
240	abnorma;	3.00	2.00	2.00
210	abnorma;	2.00	2.00	1.00
90	Normal	2.00	2.00	2.00
85	Normal	2.00	2.00	2.00
90	Normal	2.00	2.00	2.00
85	Normal	2.00	2.00	2.00
85	Normal	2.00	2.00	2.00
85	Normal	2.00	2.00	2.00
90	Normal	2.00	2.00	2.00
85	Normal	2.00	2.00	2.00

90	Normal	2.00	2.00	2.00
85	Normal	2.00	2.00	2.00
220	abnorma;	2.00	2.00	2.00
230	abnorma;	3.00	2.00	2.00
220	abnorma;	2.00	2.00	2.00
230	abnorma;	3.00	2.00	2.00
220	abnorma;	2.00	2.00	2.00
240	abnorma;	3.00	2.00	2.00
220	abnorma;	2.00	2.00	2.00
240	abnorma;	3.00	2.00	2.00
220	abnorma;	2.00	2.00	2.00
230	abnorma;	3.00	2.00	2.00
75	Normal	1.00	2.00	2.00
240	abnorma;	3.00	2.00	2.00
75	Normal	1.00	2.00	2.00
240	abnorma;	3.00	2.00	2.00
75	Normal	1.00	2.00	2.00
240	abnorma;	3.00	2.00	2.00
75	Normal	1.00	2.00	2.00
250	abnorma;	3.00	2.00	2.00
75	Normal	1.00	2.00	2.00
250	abnorma;	3.00	2.00	2.00
176	abnorma;	2.00	2.00	2.00
230	abnorma;	3.00	2.00	2.00
176	abnorma;	2.00	2.00	2.00
230	abnorma;	3.00	2.00	2.00
250	abnorma;	2.00	2.00	2.00
230	abnorma;	3.00	2.00	2.00
175	abnorma;	2.00	2.00	2.00
230	abnorma;	3.00	2.00	2.00
175	abnorma;	2.00	2.00	2.00
230	abnorma;	3.00	2.00	2.00
175	abnorma;	2.00	2.00	2.00
200	abnorma;	2.00	2.00	2.00
120	Normal	2.00	2.00	2.00
220	abnorma;	2.00	2.00	2.00
65	Normal	1.00	2.00	2.00
103	Normal	2.00	2.00	1.00
170	abnorma;	2.00	2.00	2.00
70	Normal	1.00	2.00	2.00
123	Normal	2.00	2.00	2.00
60	Normal	1.00	2.00	2.00
230	abnorma;	3.00	2.00	2.00
90	Normal	2.00	2.00	2.00
115	Normal	2.00	2.00	2.00
180	abnorma;	2.00	2.00	2.00
70	Normal	1.00	2.00	2.00
85	Normal	2.00	2.00	2.00
70	Normal	1.00	2.00	2.00
75	Normal	2.00	2.00	2.00
83	Normal	2.00	2.00	2.00
70	Normal	1.00	2.00	2.00

200	abnorma;	2.00	2.00	2.00
120	Normal	2.00	2.00	2.00
230	abnorma;	2.00	2.00	2.00
65	Normal	1.00	2.00	2.00
103	Normal	2.00	2.00	1.00
170	abnorma;	2.00	2.00	2.00
70	Normal	1.00	2.00	2.00
120	Normal	2.00	2.00	2.00
60	Normal	1.00	2.00	2.00
230	abnorma;	3.00	2.00	2.00
90	Normal	2.00	2.00	2.00
115	Normal	2.00	2.00	2.00
180	abnorma;	2.00	2.00	2.00
70	Normal	1.00	2.00	2.00
85	Normal	2.00	2.00	2.00
70	Normal	1.00	2.00	2.00
75	Normal	2.00	2.00	2.00
83	Normal	2.00	2.00	2.00
70	Normal	1.00	2.00	2.00
85	Normal	2.00	2.00	2.00
125	Normal	2.00	2.00	2.00
200	abnorma;	2.00	2.00	2.00
120	Normal	2.00	2.00	2.00
230	abnorma;	2.00	2.00	2.00
65	Normal	1.00	2.00	2.00
103	Normal	2.00	2.00	1.00
170	abnorma;	2.00	2.00	2.00
70	Normal	1.00	2.00	2.00
140	Normal	2.00	2.00	2.00
60	Normal	1.00	2.00	2.00
230	abnorma;	3.00	2.00	2.00
90	Normal	2.00	2.00	2.00
115	Normal	2.00	2.00	2.00
180	abnorma;	2.00	2.00	2.00
70	Normal	1.00	2.00	2.00
85	Normal	2.00	2.00	2.00
70	Normal	1.00	2.00	2.00
75	Normal	2.00	2.00	2.00
83	Normal	2.00	2.00	2.00
200	abnorma;	2.00	2.00	2.00
120	Normal	2.00	2.00	2.00
230	abnorma;	2.00	2.00	2.00
65	Normal	1.00	2.00	2.00
103	Normal	2.00	2.00	1.00
170	abnorma;	2.00	2.00	2.00
70	Normal	1.00	2.00	2.00
110	Normal	2.00	2.00	2.00
60	Normal	1.00	2.00	2.00
230	abnorma;	3.00	2.00	2.00
90	Normal	2.00	2.00	2.00
115	Normal	2.00	2.00	2.00
180	abnorma;	2.00	2.00	2.00

70	Normal	1.00	2.00	2.00
85	Normal	2.00	2.00	2.00
70	Normal	1.00	2.00	2.00
75	Normal	2.00	2.00	2.00
83	Normal	2.00	2.00	2.00
70	Normal	1.00	2.00	2.00
85	Normal	2.00	2.00	2.00
125	Normal	2.00	2.00	2.00
210	abnorma;	2.00	2.00	2.00
120	Normal	2.00	2.00	2.00
230	abnorma;	2.00	2.00	2.00
70	Normal	1.00	2.00	2.00
103	Normal	2.00	2.00	1.00
170	abnorma;	2.00	2.00	2.00
70	Normal	1.00	2.00	2.00
130	abnorma;	2.00	2.00	2.00
60	Normal	1.00	2.00	2.00
220	abnorma;	2.00	2.00	2.00
90	Normal	2.00	2.00	2.00
115	Normal	2.00	2.00	2.00
180	abnorma;	2.00	2.00	2.00
70	Normal	1.00	2.00	2.00
85	Normal	2.00	2.00	2.00
70	Normal	1.00	2.00	2.00
75	Normal	2.00	2.00	2.00
83	Normal	2.00	2.00	2.00
75	Normal	1.00	2.00	2.00
85	Normal	2.00	2.00	2.00
125	Normal	2.00	2.00	2.00
200	abnorma;	2.00	2.00	2.00
115	Normal	2.00	2.00	2.00
230	abnorma;	2.00	2.00	2.00
70	Normal	1.00	2.00	2.00
103	Normal	2.00	2.00	1.00
170	abnorma;	2.00	2.00	2.00
70	Normal	1.00	2.00	2.00
120	Normal	2.00	2.00	2.00
65	Normal	1.00	2.00	2.00
220	abnorma;	2.00	2.00	2.00
130	abnorma;	2.00	2.00	2.00
80	Normal	2.00	2.00	2.00
135	abnorma;	2.00	2.00	2.00
80	Normal	2.00	2.00	2.00
160	abnorma;	2.00	2.00	2.00
80	Normal	2.00	2.00	2.00
135	abnorma;	2.00	2.00	2.00
80	Normal	2.00	2.00	2.00
170	abnorma;	2.00	2.00	2.00
80	Normal	2.00	2.00	2.00
170	abnorma;	2.00	2.00	2.00
90	Normal	2.00	2.00	2.00
90	Normal	2.00	2.00	2.00

85	Normal	2.00	2.00	2.00
90	Normal	2.00	2.00	2.00
90	Normal	2.00	2.00	2.00
90	Normal	2.00	2.00	2.00
87	Normal	2.00	2.00	2.00
270	abnorma;	3.00	2.00	2.00
87	Normal	2.00	2.00	2.00
250	abnorma;	3.00	2.00	2.00
87	Normal	2.00	2.00	2.00
250	abnorma;	3.00	2.00	2.00
90	Normal	2.00	2.00	2.00
250	abnorma;	3.00	2.00	2.00
87	Normal	2.00	2.00	2.00
270	abnorma;	3.00	2.00	2.00
87	Normal	2.00	2.00	2.00
250	abnorma;	3.00	2.00	2.00
75	Normal	1.00	2.00	2.00
75	Normal	1.00	2.00	2.00
75	Normal	1.00	2.00	2.00
75	Normal	1.00	2.00	2.00
75	Normal	1.00	2.00	2.00
195	abnorma;	2.00	2.00	1.00
195	abnorma;	2.00	2.00	1.00
195	abnorma;	2.00	2.00	1.00
195	abnorma;	2.00	2.00	2.00
195	abnorma;	2.00	2.00	1.00
85	Normal	2.00	2.00	2.00
85	Normal	2.00	2.00	2.00
90	Normal	2.00	2.00	2.00
80	Normal	2.00	2.00	2.00
85	Normal	2.00	2.00	2.00
85	Normal	2.00	2.00	2.00
90	Normal	2.00	2.00	2.00
80	Normal	2.00	2.00	2.00
85	Normal	2.00	2.00	2.00
85	Normal	2.00	2.00	2.00
90	Normal	2.00	2.00	2.00
80	Normal	2.00	2.00	2.00
85	Normal	2.00	2.00	2.00
85	Normal	2.00	2.00	2.00
90	Normal	2.00	2.00	2.00
80	Normal	2.00	2.00	2.00
70	Normal	1.00	2.00	2.00
85	Normal	2.00	2.00	2.00
85	Normal	2.00	1.00	2.00
90	Normal	2.00	2.00	2.00
80	Normal	2.00	2.00	2.00
70	Normal	1.00	2.00	2.00
87	Normal	2.00	2.00	2.00
77	Normal	2.00	2.00	2.00
77	Normal	2.00	2.00	2.00
77	Normal	2.00	2.00	2.00

77	Normal	2.00	2.00	2.00
77	Normal	2.00	2.00	2.00
77	Normal	2.00	2.00	2.00
120	Normal	2.00	2.00	2.00
210	abnorma;	2.00	2.00	2.00
110	Normal	2.00	2.00	2.00
210	abnorma;	2.00	2.00	2.00
110	Normal	2.00	2.00	2.00
210	abnorma;	2.00	2.00	2.00
121	Normal	2.00	2.00	2.00
210	abnorma;	2.00	2.00	2.00
123	Normal	2.00	2.00	2.00
210	abnorma;	2.00	2.00	2.00
102	Normal	2.00	2.00	2.00
102	Normal	2.00	2.00	2.00
102	Normal	2.00	2.00	2.00
102	Normal	2.00	2.00	2.00
102	Normal	2.00	2.00	2.00
100	Normal	2.00	2.00	2.00
100	Normal	2.00	2.00	2.00
100	Normal	2.00	2.00	2.00
100	Normal	2.00	2.00	2.00
100	Normal	2.00	2.00	2.00
100	Normal	2.00	2.00	2.00
90	Normal	2.00	2.00	2.00
90	Normal	2.00	2.00	2.00
85	Normal	2.00	2.00	2.00
90	Normal	2.00	2.00	2.00
90	Normal	2.00	2.00	2.00
80	Normal	2.00	2.00	2.00
90	Normal	2.00	2.00	2.00
80	Normal	2.00	2.00	2.00
90	Normal	2.00	2.00	2.00
125	Normal	2.00	2.00	2.00
120	Normal	2.00	2.00	2.00
121	Normal	2.00	2.00	2.00
119	Normal	2.00	2.00	2.00
119	Normal	2.00	2.00	2.00
115	Normal	2.00	2.00	2.00
115	Normal	2.00	2.00	2.00
115	Normal	2.00	2.00	2.00
115	Normal	2.00	2.00	2.00
115	Normal	2.00	2.00	2.00
75	Normal	1.00	2.00	2.00
82	Normal	2.00	2.00	2.00
270	abnorma;	3.00	2.00	2.00
176	abnorma;	2.00	2.00	2.00
75	Normal	1.00	2.00	2.00
82	Normal	2.00	2.00	2.00
85	Normal	2.00	2.00	2.00
270	abnorma;	3.00	2.00	2.00
176	abnorma;	2.00	2.00	2.00

80	Normal	2.00	2.00	2.00
75	Normal	1.00	2.00	2.00
82	Normal	2.00	2.00	2.00
85	Normal	2.00	2.00	1.00
270	abnorma;	3.00	2.00	2.00
176	abnorma;	2.00	2.00	2.00
75	Normal	1.00	2.00	2.00
82	Normal	2.00	2.00	2.00
85	Normal	2.00	2.00	1.00
270	abnorma;	3.00	2.00	2.00
175	abnorma;	2.00	2.00	2.00
80	Normal	2.00	2.00	2.00
75	Normal	1.00	2.00	2.00
82	Normal	2.00	2.00	2.00
85	Normal	2.00	2.00	1.00
270	abnorma;	3.00	2.00	2.00
175	abnorma;	2.00	2.00	2.00
80	Normal	2.00	2.00	2.00
75	Normal	1.00	2.00	2.00
83	Normal	2.00	2.00	2.00
85	Normal	2.00	2.00	1.00
270	abnorma;	3.00	2.00	2.00

ANXIETY_M2	MSPSS_M2	STRESS_M	DEPRESS_M	ANXIETY_M	MSPSS_M	Drugs
2.00	2.00	2.00	2.86	2.10	5.08	Iron, Folic acid, Calcium
2.00	2.00	0.80	1.67	1.10	5.83	Iron, Folic acid, Calcium
2.00	1.00	2.90	1.81	2.43	3.67	Iron, Folic acid, Calcium
2.00	2.00	2.30	2.29	2.48	5.33	Iron, Folic acid, Calcium
2.00	2.00	1.80	1.62	1.24	6.42	Iron, Folic acid, Calcium
2.00	2.00	2.20	1.71	1.05	6.25	Iron, Folic acid, Calcium
2.00	2.00	2.50	1.48	0.48	5.17	Iron, Folic acid, Calcium
2.00	1.00	2.40	1.90	2.00	4.00	Iron, Folic acid, Calcium
2.00	2.00	1.90	2.67	2.43	6.42	Iron, Folic acid, Calcium
2.00	2.00	1.60	2.38	2.05	5.33	Iron, Folic acid, Calcium
2.00	1.00	3.50	2.33	1.81	3.08	Iron, Folic acid, Calcium
2.00	2.00	2.90	1.43	1.29	4.92	Iron, Folic acid, Calcium
2.00	1.00	2.20	2.76	1.62	3.67	Iron, Folic acid, Calcium
2.00	2.00	3.30	1.90	1.43	4.58	Iron, Folic acid, Calcium
2.00	1.00	2.90	2.48	2.19	3.00	Iron, Folic acid, Calcium
2.00	1.00	3.00	2.19	2.19	3.58	Iron, Folic acid, Calcium
2.00	2.00	3.30	2.24	1.00	5.33	Iron, Folic acid, Calcium
1.00	3.00	1.80	1.00	1.05	7.00	Iron, Folic acid, Calcium
2.00	3.00	2.70	2.48	0.95	7.00	Iron, Folic acid, Calcium
2.00	2.00	3.20	2.24	1.67	5.58	Iron, Folic acid, Calcium
2.00	1.00	2.40	1.90	2.33	2.75	Iron, Folic acid, Calcium
2.00	2.00	2.70	2.00	1.33	5.50	Iron, Folic acid, Calcium
2.00	2.00	3.20	2.19	1.52	4.50	Iron, Folic acid, Calcium
2.00	1.00	2.20	2.95	2.57	2.83	Iron, Folic acid, Calcium
2.00	2.00	3.40	2.57	1.71	5.00	Iron, Folic acid, Calcium
2.00	1.00	2.60	2.38	2.10	3.00	Iron, Folic acid, Calcium
2.00	1.00	1.50	2.33	1.90	4.33	Iron, Folic acid, Calcium
2.00	2.00	2.30	2.90	2.10	5.42	Iron, Folic acid, Calcium
2.00	1.00	2.80	2.90	1.76	4.17	Iron, Folic acid, Calcium
2.00	2.00	2.90	2.29	2.24	4.75	Iron, Folic acid, Calcium
2.00	1.00	2.80	2.19	1.95	3.67	Iron, Folic acid, Calcium
2.00	2.00	0.90	2.29	2.76	5.25	Iron, Folic acid, Calcium
2.00	2.00	2.50	2.48	2.43	4.83	Iron, Folic acid, Calcium
2.00	1.00	1.70	2.19	2.10	3.58	Iron, Folic acid, Calcium
2.00	1.00	2.90	2.71	2.24	4.00	Iron, Folic acid, Calcium
2.00	1.00	2.50	2.48	2.43	1.58	Iron, Folic acid, Calcium
2.00	2.00	2.50	2.38	1.48	6.42	Iron, Folic acid, Calcium
2.00	2.00	2.90	2.05	1.71	5.67	Iron, Folic acid, Calcium
2.00	2.00	2.70	2.57	2.10	5.00	Iron, Folic acid, Calcium
2.00	2.00	1.90	2.76	2.48	4.67	Iron, Folic acid, Calcium
2.00	2.00	3.20	2.86	1.81	5.58	Iron, Folic acid, Calcium
2.00	1.00	3.00	1.52	2.29	3.67	Iron, Folic acid, Calcium
2.00	2.00	1.40	2.24	1.81	5.08	Iron, Folic acid, Calcium
2.00	2.00	2.60	2.33	2.00	5.17	Iron, Folic acid, Calcium
2.00	2.00	1.90	2.52	1.57	5.33	Iron, Folic acid, Calcium
2.00	1.00	2.30	2.95	1.90	2.75	Iron, Folic acid, Calcium
2.00	2.00	2.60	2.38	1.52	5.08	Iron, Folic acid, Calcium
2.00	1.00	2.40	2.76	2.24	4.08	Iron, Folic acid, Calcium
2.00	2.00	2.80	2.10	1.19	6.25	Iron, Folic acid, Calcium
2.00	1.00	2.30	1.90	2.33	3.58	Iron, Folic acid, Calcium
1.00	2.00	2.20	1.33	1.33	6.08	Iron, Folic acid, Calcium

2.00	2.00	2.80	1.71	2.00	5.00	Iron, Folic acid, Calcium
2.00	1.00	2.50	2.67	2.24	3.17	Iron, Folic acid, Calcium
2.00	2.00	2.20	2.52	2.29	4.50	Iron, Folic acid, Calcium
2.00	2.00	2.40	2.19	1.38	5.42	Iron, Folic acid, Calcium
2.00	1.00	2.00	2.29	1.71	2.50	Iron, Folic acid, Calcium
2.00	1.00	2.50	2.33	2.38	3.00	Iron, Folic acid, Calcium
2.00	2.00	3.00	1.90	1.71	5.25	Iron, Folic acid, Calcium
2.00	1.00	2.10	2.38	1.38	3.42	Iron, Folic acid, Calcium
2.00	2.00	2.40	2.57	1.52	5.67	Iron, Folic acid, Calcium
2.00	1.00	2.10	2.76	2.33	3.33	Iron, Folic acid, Calcium
2.00	1.00	2.50	2.52	2.52	3.42	Iron, Folic acid, Calcium
2.00	2.00	0.80	1.81	1.05	5.83	Iron, Folic acid, Calcium
2.00	2.00	2.40	1.81	1.24	6.25	Iron, Folic acid, Calcium
1.00	2.00	1.90	1.19	0.81	5.17	Iron, Folic acid, Calcium
2.00	2.00	3.20	2.76	1.81	5.58	Iron, Folic acid, Calcium
2.00	2.00	1.40	2.19	1.86	5.08	Iron, Folic acid, Calcium
2.00	1.00	2.20	2.86	2.19	2.75	Iron, Folic acid, Calcium
2.00	2.00	0.80	1.43	1.05	5.83	Iron, Folic acid, Calcium
2.00	2.00	2.40	1.62	1.05	6.25	Iron, Folic acid, Calcium
1.00	2.00	2.80	1.24	0.48	5.17	Iron, Folic acid, Calcium
2.00	2.00	3.20	2.76	1.71	5.58	Iron, Folic acid, Calcium
2.00	2.00	1.40	2.14	1.86	5.08	Iron, Folic acid, Calcium
2.00	1.00	2.60	2.90	2.10	2.75	Iron, Folic acid, Calcium
2.00	2.00	0.80	1.52	1.05	5.83	Iron, Folic acid, Calcium
2.00	2.00	2.20	1.62	1.05	6.25	Iron, Folic acid, Calcium
1.00	2.00	2.20	1.43	0.90	5.17	Iron, Folic acid, Calcium
2.00	2.00	2.80	2.95	1.71	5.58	Iron, Folic acid, Calcium
2.00	2.00	1.40	2.14	1.86	5.08	Iron, Folic acid, Calcium
2.00	1.00	2.30	2.86	1.95	2.75	Iron, Folic acid, Calcium
2.00	2.00	0.80	1.62	1.10	5.83	Iron, Folic acid, Calcium
2.00	2.00	2.40	1.81	1.19	6.25	Iron, Folic acid, Calcium
1.00	2.00	2.50	1.00	0.48	5.17	Iron, Folic acid, Calcium
2.00	2.00	3.20	2.76	1.81	5.58	Iron, Folic acid, Calcium
2.00	2.00	1.80	2.19	1.81	5.08	Iron, Folic acid, Calcium
2.00	1.00	2.60	2.76	2.19	2.75	Iron, Folic acid, Calcium
2.00	2.00	0.80	1.62	1.05	5.83	Iron, Folic acid, Calcium
2.00	2.00	2.60	1.71	1.05	6.25	Iron, Folic acid, Calcium
1.00	2.00	3.00	1.38	0.76	5.17	Iron, Folic acid, Calcium
2.00	2.00	2.80	2.86	1.71	5.58	Iron, Folic acid, Calcium
2.00	2.00	1.80	2.19	1.81	5.08	Iron, Folic acid, Calcium
2.00	1.00	2.20	3.00	1.95	2.75	Iron, Folic acid, Calcium
2.00	2.00	0.80	1.67	1.00	5.83	Iron, Folic acid, Calcium
2.00	2.00	2.40	1.81	1.14	6.25	Iron, Folic acid, Calcium
1.00	2.00	2.50	1.38	0.67	5.17	Iron, Folic acid, Calcium
2.00	2.00	1.90	2.52	1.52	5.50	Iron, Folic acid, Calcium
2.00	2.00	2.20	2.57	2.29	4.50	Iron, Folic acid, Calcium
2.00	2.00	1.80	2.62	1.52	5.50	Iron, Folic acid, Calcium
2.00	2.00	2.20	2.57	2.38	4.50	Iron, Folic acid, Calcium
2.00	2.00	1.90	2.48	1.62	5.33	Iron, Folic acid, Calcium
2.00	2.00	2.20	2.48	2.29	4.50	Iron, Folic acid, Calcium
2.00	2.00	1.80	2.57	1.52	5.50	Iron, Folic acid, Calcium
2.00	2.00	2.20	2.43	2.38	4.50	Iron, Folic acid, Calcium

2.00	2.00	1.80	2.62	1.52	5.42	Iron, Folic acid, Calcium
2.00	2.00	2.20	2.48	2.38	4.50	Iron, Folic acid, Calcium
2.00	2.00	1.90	2.76	2.48	4.67	Iron, Folic acid, Calcium
2.00	2.00	2.80	1.62	2.00	5.00	Iron, Folic acid, Calcium
2.00	2.00	1.90	2.62	2.48	4.67	Iron, Folic acid, Calcium
2.00	2.00	3.00	1.71	2.00	5.00	Iron, Folic acid, Calcium
2.00	2.00	1.80	2.67	2.48	4.67	Iron, Folic acid, Calcium
2.00	2.00	3.00	1.71	2.00	5.17	Iron, Folic acid, Calcium
2.00	2.00	1.80	2.67	2.48	4.67	Iron, Folic acid, Calcium
2.00	2.00	2.80	1.81	1.90	5.17	Iron, Folic acid, Calcium
2.00	2.00	1.80	2.71	2.38	4.50	Iron, Folic acid, Calcium
2.00	2.00	2.80	1.76	1.90	5.17	Iron, Folic acid, Calcium
2.00	2.00	2.70	2.48	2.00	5.00	Iron, Folic acid, Calcium
2.00	2.00	2.60	2.29	1.52	5.08	Iron, Folic acid, Calcium
2.00	2.00	2.70	2.48	2.00	5.00	Iron, Folic acid, Calcium
2.00	2.00	2.60	2.38	1.62	5.08	Iron, Folic acid, Calcium
2.00	2.00	2.90	2.57	2.10	5.00	Iron, Folic acid, Calcium
2.00	2.00	2.80	2.48	1.62	5.00	Iron, Folic acid, Calcium
2.00	2.00	2.50	2.48	2.10	5.00	Iron, Folic acid, Calcium
2.00	2.00	2.60	2.38	1.52	5.00	Iron, Folic acid, Calcium
2.00	2.00	2.50	2.48	2.10	5.00	Iron, Folic acid, Calcium
2.00	2.00	2.60	2.38	1.62	5.00	Iron, Folic acid, Calcium
2.00	2.00	3.40	2.10	1.52	5.58	Iron, Folic acid, Calcium
2.00	2.00	2.60	2.24	2.00	5.17	Iron, Folic acid, Calcium
2.00	2.00	3.40	2.10	1.52	5.58	Iron, Folic acid, Calcium
2.00	2.00	2.60	2.33	2.00	5.17	Iron, Folic acid, Calcium
2.00	2.00	3.40	2.10	1.52	5.58	Iron, Folic acid, Calcium
2.00	2.00	2.80	2.43	2.00	5.17	Iron, Folic acid, Calcium
2.00	2.00	3.50	2.14	1.48	5.58	Iron, Folic acid, Calcium
2.00	2.00	2.80	2.43	1.90	5.17	Iron, Folic acid, Calcium
2.00	2.00	3.50	2.14	1.67	5.58	Iron, Folic acid, Calcium
2.00	2.00	2.80	2.52	2.00	5.17	Iron, Folic acid, Calcium
2.00	2.00	3.10	2.14	1.67	5.58	Iron, Folic acid, Calcium
2.00	2.00	1.90	2.71	2.43	6.42	Iron, Folic acid, Calcium
2.00	2.00	1.60	2.38	1.95	5.33	Iron, Folic acid, Calcium
2.00	2.00	2.80	1.67	1.29	4.92	Iron, Folic acid, Calcium
2.00	1.00	3.00	2.48	2.19	3.58	Iron, Folic acid, Calcium
1.00	3.00	1.80	1.10	0.81	7.00	Iron, Folic acid, Calcium
2.00	3.00	2.70	2.48	0.95	7.00	Iron, Folic acid, Calcium
2.00	1.00	2.70	1.95	2.24	2.75	Iron, Folic acid, Calcium
2.00	2.00	3.20	2.29	1.52	4.50	Iron, Folic acid, Calcium
2.00	1.00	2.20	2.95	2.52	2.83	Iron, Folic acid, Calcium
2.00	2.00	3.40	2.76	1.71	5.00	Iron, Folic acid, Calcium
2.00	1.00	2.60	2.62	2.05	4.17	Iron, Folic acid, Calcium
2.00	2.00	2.90	2.19	2.33	4.75	Iron, Folic acid, Calcium
2.00	1.00	3.00	2.24	2.05	3.67	Iron, Folic acid, Calcium
2.00	2.00	1.70	2.19	2.76	5.25	Iron, Folic acid, Calcium
2.00	2.00	2.50	2.48	2.43	4.83	Iron, Folic acid, Calcium
2.00	1.00	2.50	2.48	2.43	1.58	Iron, Folic acid, Calcium
2.00	1.00	2.40	2.00	2.24	3.58	Iron, Folic acid, Calcium
2.00	1.00	2.50	2.71	2.24	3.42	Iron, Folic acid, Calcium
2.00	1.00	2.50	2.33	2.38	3.00	Iron, Folic acid, Calcium

2.00	2.00	1.90	2.71	2.29	6.42	Iron, Folic acid, Calcium
2.00	2.00	1.60	2.38	1.95	5.33	Iron, Folic acid, Calcium
2.00	2.00	2.90	1.52	1.29	4.92	Iron, Folic acid, Calcium
2.00	1.00	3.00	2.14	2.19	3.58	Iron, Folic acid, Calcium
1.00	2.00	1.80	1.05	0.81	7.00	Iron, Folic acid, Calcium
2.00	3.00	2.70	2.48	0.95	7.00	Iron, Folic acid, Calcium
2.00	1.00	2.40	1.81	2.33	2.75	Iron, Folic acid, Calcium
2.00	2.00	3.20	2.19	1.52	4.50	Iron, Folic acid, Calcium
2.00	1.00	2.20	2.90	2.52	2.83	Iron, Folic acid, Calcium
2.00	2.00	3.40	2.67	1.71	5.00	Iron, Folic acid, Calcium
2.00	1.00	2.80	2.86	1.95	4.17	Iron, Folic acid, Calcium
2.00	2.00	2.90	2.29	2.33	4.75	Iron, Folic acid, Calcium
2.00	1.00	2.80	2.14	1.95	3.67	Iron, Folic acid, Calcium
2.00	2.00	1.70	2.19	2.76	5.25	Iron, Folic acid, Calcium
2.00	2.00	2.50	2.48	2.43	4.83	Iron, Folic acid, Calcium
2.00	1.00	2.30	2.67	2.43	0.75	Iron, Folic acid, Calcium
2.00	1.00	2.40	2.00	2.24	3.58	Iron, Folic acid, Calcium
2.00	1.00	2.50	2.71	2.24	3.42	Iron, Folic acid, Calcium
2.00	1.00	2.50	2.38	2.33	3.00	Iron, Folic acid, Calcium
2.00	1.00	2.10	2.33	1.38	3.00	Iron, Folic acid, Calcium
2.00	2.00	2.40	2.57	1.62	5.67	Iron, Folic acid, Calcium
2.00	2.00	1.90	2.71	2.43	6.42	Iron, Folic acid, Calcium
2.00	2.00	1.60	2.29	1.95	5.33	Iron, Folic acid, Calcium
2.00	2.00	3.10	1.62	1.29	4.92	Iron, Folic acid, Calcium
2.00	1.00	2.90	2.48	2.33	3.58	Iron, Folic acid, Calcium
1.00	2.00	1.80	1.19	0.57	7.00	Iron, Folic acid, Calcium
2.00	3.00	2.50	2.48	0.95	7.00	Iron, Folic acid, Calcium
2.00	1.00	2.70	1.95	2.19	2.75	Iron, Folic acid, Calcium
2.00	2.00	3.30	2.24	1.52	4.50	Iron, Folic acid, Calcium
2.00	1.00	2.30	2.95	2.52	2.83	Iron, Folic acid, Calcium
2.00	2.00	3.40	2.67	1.62	5.00	Iron, Folic acid, Calcium
2.00	1.00	2.60	2.57	2.00	4.17	Iron, Folic acid, Calcium
2.00	2.00	3.10	2.29	2.33	4.75	Iron, Folic acid, Calcium
2.00	1.00	3.00	2.29	2.05	3.67	Iron, Folic acid, Calcium
2.00	2.00	2.50	2.19	2.76	5.25	Iron, Folic acid, Calcium
2.00	2.00	2.30	2.38	2.52	4.83	Iron, Folic acid, Calcium
2.00	1.00	2.30	2.62	2.43	0.75	Iron, Folic acid, Calcium
2.00	1.00	2.30	2.00	2.24	3.58	Iron, Folic acid, Calcium
2.00	1.00	2.30	2.62	2.24	3.42	Iron, Folic acid, Calcium
2.00	2.00	1.90	2.76	2.43	6.42	Iron, Folic acid, Calcium
2.00	2.00	1.60	2.29	1.95	5.33	Iron, Folic acid, Calcium
2.00	2.00	2.90	1.48	1.43	4.92	Iron, Folic acid, Calcium
2.00	1.00	2.90	2.38	2.19	3.58	Iron, Folic acid, Calcium
1.00	2.00	2.00	0.86	0.76	7.00	Iron, Folic acid, Calcium
2.00	3.00	2.50	2.48	0.95	7.00	Iron, Folic acid, Calcium
2.00	1.00	2.40	1.81	2.33	2.75	Iron, Folic acid, Calcium
2.00	2.00	3.30	2.19	1.57	4.50	Iron, Folic acid, Calcium
2.00	1.00	2.30	3.00	2.52	2.83	Iron, Folic acid, Calcium
2.00	2.00	3.40	2.67	1.71	5.00	Iron, Folic acid, Calcium
2.00	1.00	2.80	2.95	1.71	4.17	Iron, Folic acid, Calcium
2.00	2.00	2.70	2.29	2.24	4.75	Iron, Folic acid, Calcium
2.00	1.00	2.80	2.10	1.95	3.67	Iron, Folic acid, Calcium

2.00	2.00	2.30	2.00	2.76	5.25	Iron, Folic acid, Calcium
2.00	2.00	2.70	2.48	2.52	4.83	Iron, Folic acid, Calcium
2.00	1.00	2.70	2.48	2.52	2.92	Iron, Folic acid, Calcium
2.00	1.00	2.70	2.00	2.24	3.58	Iron, Folic acid, Calcium
2.00	1.00	2.50	2.67	2.43	3.42	Iron, Folic acid, Calcium
2.00	1.00	2.40	2.38	2.33	3.00	Iron, Folic acid, Calcium
2.00	1.00	2.10	2.33	1.29	3.00	Iron, Folic acid, Calcium
2.00	2.00	2.40	2.57	1.52	5.67	Iron, Folic acid, Calcium
2.00	2.00	1.90	2.67	2.38	6.42	Iron, Folic acid, Calcium
2.00	2.00	2.00	2.29	1.95	5.33	Iron, Folic acid, Calcium
2.00	2.00	2.90	1.76	1.43	4.92	Iron, Folic acid, Calcium
2.00	1.00	2.70	2.52	2.10	3.58	Iron, Folic acid, Calcium
1.00	2.00	2.40	1.10	0.71	7.00	Iron, Folic acid, Calcium
2.00	3.00	2.90	2.57	0.95	7.00	Iron, Folic acid, Calcium
2.00	1.00	2.60	1.90	2.19	2.75	Iron, Folic acid, Calcium
2.00	2.00	2.90	2.14	1.57	4.50	Iron, Folic acid, Calcium
2.00	1.00	2.50	2.95	2.52	2.83	Iron, Folic acid, Calcium
2.00	2.00	3.40	2.57	1.71	5.00	Iron, Folic acid, Calcium
2.00	1.00	2.60	2.71	1.81	4.17	Iron, Folic acid, Calcium
2.00	2.00	2.70	2.29	2.24	4.75	Iron, Folic acid, Calcium
2.00	1.00	3.00	2.29	2.05	3.67	Iron, Folic acid, Calcium
2.00	2.00	2.30	2.10	2.76	5.25	Iron, Folic acid, Calcium
2.00	2.00	2.70	2.57	2.43	4.83	Iron, Folic acid, Calcium
2.00	1.00	2.70	2.48	2.43	2.92	Iron, Folic acid, Calcium
2.00	1.00	2.70	2.00	2.24	3.58	Iron, Folic acid, Calcium
2.00	1.00	2.50	2.67	2.43	3.42	Iron, Folic acid, Calcium
2.00	1.00	2.40	2.43	2.33	3.00	Iron, Folic acid, Calcium
2.00	1.00	2.00	2.48	1.38	3.00	Iron, Folic acid, Calcium
2.00	2.00	2.20	2.48	1.52	5.67	Iron, Folic acid, Calcium
2.00	2.00	1.90	2.71	2.24	6.42	Iron, Folic acid, Calcium
2.00	2.00	2.00	2.29	1.95	5.33	Iron, Folic acid, Calcium
2.00	2.00	2.90	1.81	1.33	4.92	Iron, Folic acid, Calcium
2.00	1.00	2.60	2.48	2.10	3.58	Iron, Folic acid, Calcium
1.00	2.00	2.00	0.90	0.67	7.00	Iron, Folic acid, Calcium
2.00	3.00	2.70	2.48	0.95	7.00	Iron, Folic acid, Calcium
2.00	1.00	2.70	1.95	2.33	2.75	Iron, Folic acid, Calcium
2.00	2.00	3.00	2.24	1.57	4.50	Iron, Folic acid, Calcium
2.00	1.00	2.60	3.00	2.52	2.83	Iron, Folic acid, Calcium
2.00	2.00	3.40	2.67	1.71	5.00	Iron, Folic acid, Calcium
2.00	2.00	2.30	2.29	2.48	5.33	Iron, Folic acid, Calcium
2.00	1.00	2.90	2.71	2.24	4.00	Iron, Folic acid, Calcium
2.00	2.00	2.30	2.29	2.43	5.33	Iron, Folic acid, Calcium
2.00	1.00	2.90	2.71	2.24	4.00	Iron, Folic acid, Calcium
2.00	2.00	2.30	2.29	2.48	5.33	Iron, Folic acid, Calcium
2.00	1.00	2.70	2.67	2.29	4.00	Iron, Folic acid, Calcium
2.00	2.00	2.30	2.29	2.48	5.42	Iron, Folic acid, Calcium
2.00	1.00	2.70	2.76	2.19	4.00	Iron, Folic acid, Calcium
2.00	2.00	2.40	2.24	2.48	5.42	Iron, Folic acid, Calcium
2.00	1.00	2.70	2.67	2.19	4.00	Iron, Folic acid, Calcium
2.00	2.00	2.40	2.19	2.43	5.42	Iron, Folic acid, Calcium
2.00	1.00	3.10	2.62	1.81	3.08	Iron, Folic acid, Calcium
2.00	1.00	3.50	2.33	2.00	3.08	Iron, Folic acid, Calcium

2.00	1.00	3.10	2.62	2.00	3.08	Iron, Folic acid, Calcium
2.00	1.00	3.50	2.33	1.76	3.08	Iron, Folic acid, Calcium
2.00	1.00	3.50	2.62	2.05	3.08	Iron, Folic acid, Calcium
2.00	1.00	3.50	2.52	1.76	3.08	Iron, Folic acid, Calcium
2.00	1.00	2.40	1.81	2.05	4.00	Iron, Folic acid, Calcium
2.00	2.00	3.30	2.24	1.00	5.25	Iron, Folic acid, Calcium
2.00	1.00	1.80	1.76	2.00	4.00	Iron, Folic acid, Calcium
2.00	2.00	3.30	2.24	0.95	5.25	Iron, Folic acid, Calcium
2.00	1.00	2.40	1.95	2.00	4.00	Iron, Folic acid, Calcium
2.00	2.00	3.30	2.00	1.05	5.33	Iron, Folic acid, Calcium
2.00	1.00	1.20	1.81	2.10	4.00	Iron, Folic acid, Calcium
2.00	2.00	3.30	2.00	0.95	5.33	Iron, Folic acid, Calcium
2.00	1.00	2.60	1.90	2.19	4.00	Iron, Folic acid, Calcium
2.00	2.00	3.30	2.05	1.00	5.33	Iron, Folic acid, Calcium
2.00	1.00	1.80	1.71	2.14	4.00	Iron, Folic acid, Calcium
2.00	2.00	3.30	2.10	1.00	5.33	Iron, Folic acid, Calcium
2.00	1.00	2.40	2.76	2.24	4.08	Iron, Folic acid, Calcium
2.00	1.00	2.40	2.86	2.14	4.08	Iron, Folic acid, Calcium
2.00	1.00	2.30	2.81	2.24	4.08	Iron, Folic acid, Calcium
2.00	1.00	2.10	2.71	2.24	4.08	Iron, Folic acid, Calcium
2.00	1.00	2.10	2.71	2.33	4.08	Iron, Folic acid, Calcium
1.00	2.00	2.20	1.19	1.33	6.08	Iron, Folic acid, Calcium
1.00	2.00	1.60	1.33	1.33	6.08	Iron, Folic acid, Calcium
1.00	2.00	1.60	1.24	1.33	6.08	Iron, Folic acid, Calcium
2.00	2.00	1.60	1.43	1.33	6.08	Iron, Folic acid, Calcium
1.00	2.00	1.30	1.19	1.33	6.08	Iron, Folic acid, Calcium
2.00	1.00	2.20	2.76	1.62	3.67	Iron, Folic acid, Calcium
2.00	1.00	1.50	2.33	1.81	4.33	Iron, Folic acid, Calcium
2.00	2.00	2.50	2.38	1.48	6.42	Iron, Folic acid, Calcium
2.00	1.00	2.40	2.19	1.81	2.50	Iron, Folic acid, Calcium
2.00	1.00	2.20	2.76	1.71	3.67	Iron, Folic acid, Calcium
2.00	1.00	1.10	2.14	1.90	4.33	Iron, Folic acid, Calcium
2.00	2.00	2.50	2.43	1.52	6.42	Iron, Folic acid, Calcium
2.00	1.00	2.00	2.29	1.71	2.50	Iron, Folic acid, Calcium
2.00	1.00	2.00	2.67	1.71	3.67	Iron, Folic acid, Calcium
2.00	1.00	1.10	2.05	2.00	4.33	Iron, Folic acid, Calcium
2.00	2.00	2.30	2.24	1.43	6.42	Iron, Folic acid, Calcium
2.00	1.00	2.00	2.19	1.81	2.50	Iron, Folic acid, Calcium
2.00	1.00	2.00	2.67	1.71	3.67	Iron, Folic acid, Calcium
2.00	1.00	1.10	2.10	1.90	4.33	Iron, Folic acid, Calcium
2.00	2.00	2.30	2.24	1.43	6.42	Iron, Folic acid, Calcium
2.00	1.00	2.00	2.19	1.71	2.50	Iron, Folic acid, Calcium
2.00	1.00	2.30	2.43	2.52	3.00	Iron, Folic acid, Calcium
2.00	1.00	2.40	2.76	1.71	3.67	Iron, Folic acid, Calcium
2.00	1.00	0.70	1.90	1.95	4.33	Iron, Folic acid, Calcium
2.00	2.00	2.30	2.33	1.48	6.42	Iron, Folic acid, Calcium
2.00	1.00	2.40	2.19	1.81	2.50	Iron, Folic acid, Calcium
2.00	1.00	2.30	2.43	2.52	3.33	Iron, Folic acid, Calcium
2.00	1.00	2.20	2.67	1.71	3.67	Iron, Folic acid, Calcium
2.00	1.00	2.90	2.52	2.19	3.00	Iron, Folic acid, Calcium
2.00	1.00	2.90	2.52	2.14	3.00	Iron, Folic acid, Calcium
2.00	1.00	2.80	2.57	2.24	3.00	Iron, Folic acid, Calcium

2.00	1.00	2.80	2.57	2.19	3.00	Iron, Folic acid, Calcium
2.00	1.00	2.60	2.67	2.19	3.00	Iron, Folic acid, Calcium
2.00	1.00	2.50	2.62	2.19	2.75	Iron, Folic acid, Calcium
2.00	2.00	2.90	2.05	1.71	5.67	Iron, Folic acid, Calcium
2.00	2.00	2.40	2.00	1.38	5.42	Iron, Folic acid, Calcium
2.00	2.00	2.90	2.10	1.67	5.67	Iron, Folic acid, Calcium
2.00	2.00	2.40	2.10	1.38	5.42	Iron, Folic acid, Calcium
2.00	2.00	3.10	2.10	1.67	5.67	Iron, Folic acid, Calcium
2.00	2.00	2.60	2.19	1.38	5.42	Iron, Folic acid, Calcium
2.00	2.00	2.70	2.10	1.71	5.67	Iron, Folic acid, Calcium
2.00	2.00	2.60	2.19	1.33	5.42	Iron, Folic acid, Calcium
2.00	2.00	2.70	2.00	1.76	5.67	Iron, Folic acid, Calcium
2.00	2.00	2.60	2.10	1.33	5.42	Iron, Folic acid, Calcium
2.00	1.00	3.00	1.67	2.38	3.67	Iron, Folic acid, Calcium
2.00	1.00	3.00	1.67	2.38	3.67	Iron, Folic acid, Calcium
2.00	1.00	3.00	1.86	2.19	3.67	Iron, Folic acid, Calcium
2.00	1.00	3.00	1.81	2.19	3.67	Iron, Folic acid, Calcium
2.00	1.00	3.00	2.00	2.19	3.67	Iron, Folic acid, Calcium
2.00	2.00	3.30	2.00	1.43	4.58	Iron, Folic acid, Calcium
2.00	2.00	3.30	2.00	1.43	4.58	Iron, Folic acid, Calcium
2.00	2.00	3.30	2.00	1.43	4.58	Iron, Folic acid, Calcium
2.00	2.00	3.30	2.00	1.43	4.58	Iron, Folic acid, Calcium
2.00	2.00	2.90	2.10	1.33	4.58	Iron, Folic acid, Calcium
2.00	2.00	2.90	2.10	1.33	4.58	Iron, Folic acid, Calcium
2.00	1.00	2.60	2.33	2.10	3.00	Iron, Folic acid, Calcium
2.00	1.00	2.60	2.52	2.10	3.00	Iron, Folic acid, Calcium
2.00	1.00	2.10	2.76	2.33	3.17	Iron, Folic acid, Calcium
2.00	1.00	2.60	2.52	2.10	3.00	Iron, Folic acid, Calcium
2.00	1.00	2.60	2.71	2.05	3.00	Iron, Folic acid, Calcium
2.00	1.00	2.10	2.67	2.43	3.17	Iron, Folic acid, Calcium
2.00	1.00	2.80	2.67	2.05	3.00	Iron, Folic acid, Calcium
2.00	1.00	2.10	2.62	2.43	3.17	Iron, Folic acid, Calcium
2.00	1.00	2.80	2.67	2.05	3.00	Iron, Folic acid, Calcium
2.00	1.00	1.60	2.62	1.90	3.75	Iron, Folic acid, Calcium
2.00	1.00	1.60	2.62	2.00	3.75	Iron, Folic acid, Calcium
2.00	1.00	1.60	2.62	1.95	3.75	Iron, Folic acid, Calcium
2.00	1.00	1.60	2.62	1.95	3.75	Iron, Folic acid, Calcium
2.00	1.00	1.60	2.67	1.95	3.75	Iron, Folic acid, Calcium
2.00	2.00	2.30	2.90	2.10	5.42	Iron, Folic acid, Calcium
2.00	2.00	2.10	2.81	2.10	5.42	Iron, Folic acid, Calcium
2.00	2.00	2.10	2.81	2.10	5.42	Iron, Folic acid, Calcium
2.00	2.00	2.10	2.62	2.19	5.42	Iron, Folic acid, Calcium
2.00	2.00	2.10	2.71	2.19	5.42	Iron, Folic acid, Calcium
2.00	2.00	1.80	2.95	2.10	5.08	Iron, Folic acid, Calcium
2.00	1.00	2.90	2.00	2.24	3.67	Iron, Folic acid, Calcium
2.00	2.00	2.70	2.10	1.33	5.50	Iron, Folic acid, Calcium
2.00	2.00	2.80	2.19	1.19	6.25	Iron, Folic acid, Calcium
2.00	2.00	1.80	2.86	2.05	5.08	Iron, Folic acid, Calcium
2.00	1.00	2.90	2.14	2.33	3.67	Iron, Folic acid, Calcium
2.00	2.00	1.80	1.48	1.33	6.42	Iron, Folic acid, Calcium
2.00	2.00	2.70	2.10	1.33	5.50	Iron, Folic acid, Calcium
2.00	2.00	2.80	2.29	1.19	6.25	Iron, Folic acid, Calcium

2.00	2.00	3.00	1.86	1.71	5.25	Iron, Folic acid, Calcium
2.00	2.00	1.80	2.76	2.05	5.08	Iron, Folic acid, Calcium
2.00	1.00	2.90	2.14	2.33	3.67	Iron, Folic acid, Calcium
1.00	2.00	2.00	0.95	1.05	6.42	Iron, Folic acid, Calcium
2.00	2.00	2.90	2.19	1.24	5.50	Iron, Folic acid, Calcium
2.00	2.00	2.80	2.29	1.19	6.25	Iron, Folic acid, Calcium
2.00	2.00	1.80	4.19	2.19	5.08	Iron, Folic acid, Calcium
2.00	1.00	2.90	2.29	2.33	3.67	Iron, Folic acid, Calcium
1.00	2.00	2.00	1.24	1.33	6.42	Iron, Folic acid, Calcium
2.00	2.00	2.90	2.19	1.33	5.50	Iron, Folic acid, Calcium
2.00	2.00	2.40	2.19	1.19	6.25	Iron, Folic acid, Calcium
2.00	2.00	3.00	1.81	1.76	5.25	Iron, Folic acid, Calcium
2.00	2.00	1.80	2.71	2.19	5.08	Iron, Folic acid, Calcium
2.00	1.00	2.90	2.43	2.33	3.67	Iron, Folic acid, Calcium
1.00	2.00	1.80	1.24	1.33	6.42	Iron, Folic acid, Calcium
2.00	2.00	2.90	2.10	1.43	5.50	Iron, Folic acid, Calcium
2.00	2.00	2.40	2.19	1.19	6.25	Iron, Folic acid, Calcium
2.00	2.00	3.20	1.86	1.76	5.25	Iron, Folic acid, Calcium
2.00	2.00	1.80	2.71	2.19	5.08	Iron, Folic acid, Calcium
2.00	1.00	2.90	2.43	2.33	3.67	Iron, Folic acid, Calcium
1.00	2.00	2.00	1.19	1.14	6.42	Iron, Folic acid, Calcium
2.00	2.00	3.10	2.29	1.43	5.50	Iron, Folic acid, Calcium

STRESS	Stress1	Stress2	Stress3	Stress4	Stress5	Stress6	Stress7
#NULL!	2	2	2	2	2	2	2
#NULL!	0	2	0	0	2	0	2
#NULL!	3	4	3	3	4	3	0
#NULL!	2	2	2	2	2	2	2
#NULL!	2	2	2	2	2	2	2
#NULL!	2	2	2	2	2	2	2
#NULL!	4	2	3	4	2	3	2
#NULL!	4	0	4	4	2	4	0
#NULL!	2	2	2	2	2	2	3
#NULL!	2	0	2	2	0	2	0
#NULL!	4	2	4	4	3	4	4
#NULL!	4	2	4	4	2	4	3
#NULL!	2	2	2	2	0	2	2
#NULL!	4	0	4	4	3	4	3
#NULL!	3	3	3	3	2	3	0
#NULL!	3	3	3	3	0	3	3
#NULL!	4	2	4	4	2	4	2
#NULL!	1	2	1	1	2	1	3
#NULL!	2	2	2	2	2	2	4
#NULL!	4	2	2	4	4	2	3
#NULL!	3	4	3	3	0	3	0
#NULL!	4	2	4	4	2	4	3
#NULL!	4	2	4	4	3	4	3
#NULL!	3	4	3	3	0	3	0
#NULL!	4	0	4	4	4	4	4
#NULL!	3	4	3	3	0	3	2
#NULL!	0	2	0	0	3	0	0
#NULL!	2	2	2	2	3	2	0
#NULL!	2	2	2	2	4	2	4
#NULL!	4	3	4	4	2	4	2
#NULL!	4	0	4	4	4	4	2
#NULL!	0	2	0	0	2	0	2
#NULL!	2	2	2	2	2	2	2
#NULL!	2	2	2	2	0	2	0
#NULL!	2	4	2	2	3	2	2
#NULL!	2	4	3	2	2	3	3
#NULL!	2	0	2	2	3	2	4
#NULL!	4	2	4	4	3	4	2
#NULL!	4	2	4	4	2	4	0
#NULL!	2	3	2	2	0	2	0
#NULL!	4	0	4	4	2	4	3
#NULL!	3	4	3	3	2	3	2
#NULL!	2	0	2	2	2	2	0
#NULL!	4	0	4	4	2	4	4
#NULL!	2	0	2	2	0	2	2
#NULL!	2	4	2	2	2	2	0
#NULL!	4	0	4	4	3	4	3
#NULL!	3	2	3	3	0	3	0
#NULL!	4	0	4	4	2	4	0
#NULL!	2	4	2	2	2	2	0
#NULL!	4	0	1	4	2	1	4

#NULL!	4	2	4	4	2	4	2
#NULL!	2	3	2	2	0	2	2
#NULL!	2	2	2	2	2	2	4
#NULL!	4	2	4	4	0	4	2
#NULL!	2	3	2	2	0	2	2
#NULL!	3	3	3	3	0	3	2
#NULL!	4	2	4	4	2	4	4
#NULL!	2	3	2	2	0	2	2
#NULL!	2	0	2	2	3	2	3
#NULL!	2	3	2	2	0	2	2
#NULL!	3	3	3	3	0	3	2
#NULL!	0	2	0	0	2	0	2
#NULL!	2	2	2	2	2	2	2
#NULL!	2	2	2	2	2	2	2
#NULL!	4	0	4	4	2	4	3
#NULL!	2	0	2	2	2	2	0
#NULL!	2	4	2	2	2	2	0
#NULL!	0	2	0	0	2	0	2
#NULL!	2	2	4	2	2	4	2
#NULL!	4	2	4	4	2	4	2
#NULL!	4	0	4	4	2	4	3
#NULL!	2	0	2	2	2	2	0
#NULL!	2	4	4	2	2	4	0
#NULL!	0	2	0	0	2	0	2
#NULL!	2	2	2	2	2	2	2
#NULL!	2	2	2	2	2	2	2
#NULL!	4	0	2	4	2	2	3
#NULL!	2	0	2	2	2	2	0
#NULL!	2	4	2	2	2	2	0
#NULL!	0	2	0	0	2	0	2
#NULL!	2	2	2	2	2	2	2
#NULL!	3	2	4	3	2	4	2
#NULL!	4	0	4	4	2	4	3
#NULL!	2	0	4	2	2	4	0
#NULL!	2	4	4	2	2	4	0
#NULL!	0	2	0	0	2	0	2
#NULL!	2	2	4	2	2	4	2
#NULL!	4	2	4	4	2	4	2
#NULL!	4	0	2	4	2	2	3
#NULL!	2	0	4	2	2	4	0
#NULL!	2	4	2	2	2	2	0
#NULL!	0	2	0	0	2	0	2
#NULL!	2	2	2	2	2	2	2
#NULL!	2	2	4	2	2	4	2
#NULL!	2	0	2	2	0	2	2
#NULL!	2	2	2	2	2	2	4
#NULL!	2	0	2	2	0	2	2
#NULL!	2	2	2	2	2	2	4
#NULL!	2	0	2	2	0	2	2
#NULL!	2	2	2	2	2	2	4
#NULL!	2	0	2	2	0	2	2
#NULL!	2	2	2	2	2	2	4

#NULL!	2	0	2	2	0	2	2
#NULL!	2	2	2	2	2	2	4
#NULL!	2	3	2	2	0	2	0
#NULL!	4	2	4	4	2	4	2
#NULL!	2	3	2	2	0	2	0
#NULL!	4	2	4	4	2	4	2
#NULL!	2	3	2	2	0	2	0
#NULL!	4	2	4	4	2	4	2
#NULL!	2	3	2	2	0	2	0
#NULL!	4	2	3	4	2	3	2
#NULL!	2	3	2	2	0	2	0
#NULL!	4	2	3	4	2	3	2
#NULL!	4	2	4	4	2	4	0
#NULL!	4	0	4	4	3	4	3
#NULL!	4	2	4	4	2	4	0
#NULL!	4	0	4	4	3	4	3
#NULL!	4	2	4	4	2	4	0
#NULL!	4	0	4	4	3	4	3
#NULL!	4	2	2	4	2	2	0
#NULL!	4	0	3	4	3	3	3
#NULL!	4	2	2	4	2	2	0
#NULL!	4	0	3	4	3	3	3
#NULL!	4	2	4	4	4	4	3
#NULL!	4	0	4	4	2	4	4
#NULL!	4	2	4	4	4	4	3
#NULL!	4	0	4	4	2	4	4
#NULL!	4	2	4	4	4	4	3
#NULL!	4	0	4	4	2	4	4
#NULL!	4	2	4	4	4	4	3
#NULL!	4	0	4	4	2	4	4
#NULL!	4	2	4	4	4	4	3
#NULL!	4	0	4	4	2	4	4
#NULL!	4	2	2	4	4	2	3
#NULL!	2	2	2	2	2	2	3
#NULL!	2	0	2	2	0	2	0
#NULL!	4	2	4	4	2	4	3
#NULL!	3	3	3	3	0	3	3
#NULL!	1	2	1	1	2	1	3
#NULL!	2	2	2	2	2	2	4
#NULL!	3	4	3	3	0	3	0
#NULL!	4	2	4	4	3	4	3
#NULL!	3	4	3	3	0	3	0
#NULL!	4	0	4	4	4	4	4
#NULL!	2	2	2	2	4	2	4
#NULL!	4	3	4	4	2	4	2
#NULL!	4	0	4	4	4	4	2
#NULL!	0	2	4	0	2	4	2
#NULL!	2	2	2	2	2	2	2
#NULL!	2	4	3	2	2	3	3
#NULL!	2	4	2	2	2	2	0
#NULL!	2	3	2	2	0	2	2
#NULL!	3	3	3	3	0	3	2

#NULL!	2	2	2	2	2	2	3
#NULL!	2	0	2	2	0	2	0
#NULL!	4	2	4	4	2	4	3
#NULL!	3	3	3	3	0	3	3
#NULL!	1	2	2	1	2	2	3
#NULL!	2	2	2	2	2	2	4
#NULL!	3	4	3	3	0	3	0
#NULL!	4	2	4	4	3	4	3
#NULL!	3	4	3	3	0	3	0
#NULL!	4	0	4	4	4	4	4
#NULL!	2	2	2	2	4	2	4
#NULL!	4	3	4	4	2	4	2
#NULL!	4	0	4	4	4	4	2
#NULL!	0	2	4	0	2	4	2
#NULL!	2	2	2	2	2	2	2
#NULL!	2	4	2	2	2	2	3
#NULL!	2	4	2	2	2	2	0
#NULL!	2	3	2	2	0	2	2
#NULL!	3	3	3	3	0	3	2
#NULL!	2	3	2	2	0	2	2
#NULL!	2	0	2	2	3	2	3
#NULL!	2	2	2	2	2	2	3
#NULL!	2	0	2	2	0	2	0
#NULL!	4	2	4	4	2	4	3
#NULL!	3	3	3	3	0	3	3
#NULL!	1	2	2	1	2	2	3
#NULL!	2	2	2	2	2	2	4
#NULL!	3	4	3	3	0	3	0
#NULL!	4	2	4	4	3	4	3
#NULL!	3	4	3	3	0	3	0
#NULL!	4	0	4	4	4	4	4
#NULL!	2	2	2	2	4	2	4
#NULL!	4	3	4	4	2	4	2
#NULL!	4	0	4	4	4	4	2
#NULL!	4	2	4	4	2	4	2
#NULL!	2	2	2	2	2	2	2
#NULL!	2	4	2	2	2	2	3
#NULL!	2	4	2	2	2	2	0
#NULL!	2	3	2	2	0	2	2
#NULL!	2	2	2	2	2	2	3
#NULL!	2	0	2	2	0	2	0
#NULL!	4	2	4	4	2	4	3
#NULL!	3	3	3	3	0	3	3
#NULL!	2	2	2	2	2	2	3
#NULL!	2	2	2	2	2	2	4
#NULL!	3	4	3	3	0	3	0
#NULL!	4	2	4	4	3	4	3
#NULL!	3	4	3	3	0	3	0
#NULL!	4	0	4	4	4	4	4
#NULL!	2	2	2	2	4	2	4
#NULL!	4	3	2	4	2	2	2
#NULL!	4	0	4	4	4	4	2

#NULL!	4	2	3	4	2	3	2
#NULL!	2	2	4	2	2	4	2
#NULL!	2	4	4	2	2	4	3
#NULL!	2	4	4	2	2	4	0
#NULL!	2	3	3	2	0	3	2
#NULL!	3	3	2	3	0	2	2
#NULL!	2	3	2	2	0	2	2
#NULL!	2	0	2	2	3	2	3
#NULL!	2	2	2	2	2	2	3
#NULL!	2	0	4	2	0	4	0
#NULL!	4	2	3	4	2	3	3
#NULL!	3	3	2	3	0	2	3
#NULL!	2	2	4	2	2	4	3
#NULL!	2	2	4	2	2	4	4
#NULL!	3	4	3	3	0	3	0
#NULL!	4	2	2	4	3	2	3
#NULL!	3	4	4	3	0	4	0
#NULL!	4	0	4	4	4	4	4
#NULL!	2	2	2	2	4	2	4
#NULL!	4	3	2	4	2	2	2
#NULL!	4	0	4	4	4	4	2
#NULL!	4	2	3	4	2	3	2
#NULL!	2	2	4	2	2	4	2
#NULL!	2	4	4	2	2	4	3
#NULL!	2	4	4	2	2	4	0
#NULL!	2	3	3	2	0	3	2
#NULL!	3	3	2	3	0	2	2
#NULL!	2	3	2	2	0	2	2
#NULL!	2	0	2	2	3	2	3
#NULL!	2	2	2	2	2	2	3
#NULL!	2	0	4	2	0	4	0
#NULL!	4	2	3	4	2	3	3
#NULL!	3	3	2	3	0	2	3
#NULL!	2	2	2	2	2	2	3
#NULL!	2	2	4	2	2	4	4
#NULL!	3	4	3	3	0	3	0
#NULL!	4	2	2	4	3	2	3
#NULL!	3	4	4	3	0	4	0
#NULL!	4	0	4	4	4	4	4
#NULL!	2	2	2	2	2	2	2
#NULL!	2	4	2	2	3	2	2
#NULL!	2	2	2	2	2	2	2
#NULL!	2	4	2	2	3	2	2
#NULL!	2	2	2	2	2	2	2
#NULL!	2	4	2	2	3	2	2
#NULL!	2	2	2	2	2	2	2
#NULL!	2	4	2	2	3	2	2
#NULL!	2	2	3	2	2	3	2
#NULL!	2	4	2	2	3	2	2
#NULL!	2	2	3	2	2	3	2
#NULL!	4	2	2	4	3	2	4
#NULL!	4	2	4	4	3	4	4

#NULL!	4	2	2	4	3	2	4
#NULL!	4	2	4	4	3	4	4
#NULL!	4	2	4	4	3	4	4
#NULL!	4	2	4	4	3	4	4
#NULL!	4	0	4	4	2	4	0
#NULL!	4	2	4	4	2	4	2
#NULL!	4	0	0	4	2	0	0
#NULL!	4	2	4	4	2	4	2
#NULL!	4	0	4	4	2	4	0
#NULL!	4	2	4	4	2	4	2
#NULL!	0	0	0	0	2	0	0
#NULL!	4	2	4	4	2	4	2
#NULL!	4	0	4	4	2	4	0
#NULL!	4	2	4	4	2	4	2
#NULL!	4	0	0	4	2	0	0
#NULL!	4	2	4	4	2	4	2
#NULL!	3	2	3	3	0	3	0
#NULL!	3	2	3	3	0	3	0
#NULL!	3	2	3	3	0	3	0
#NULL!	3	2	2	3	0	2	0
#NULL!	3	2	2	3	0	2	0
#NULL!	4	0	1	4	2	1	4
#NULL!	1	0	1	1	2	1	4
#NULL!	1	0	1	1	2	1	4
#NULL!	1	0	1	1	2	1	4
#NULL!	1	0	1	1	2	1	4
#NULL!	2	2	2	2	0	2	2
#NULL!	0	2	0	0	3	0	0
#NULL!	2	0	2	2	3	2	4
#NULL!	2	3	4	2	0	4	2
#NULL!	2	2	2	2	0	2	2
#NULL!	0	2	0	0	3	0	0
#NULL!	2	0	2	2	3	2	4
#NULL!	2	3	2	2	0	2	2
#NULL!	2	2	2	2	0	2	2
#NULL!	0	2	0	0	3	0	0
#NULL!	2	0	2	2	3	2	4
#NULL!	2	3	2	2	0	2	2
#NULL!	2	2	2	2	0	2	2
#NULL!	0	2	0	0	3	0	0
#NULL!	2	0	2	2	3	2	4
#NULL!	2	3	2	2	0	2	2
#NULL!	3	3	2	3	0	2	2
#NULL!	2	2	4	2	0	4	2
#NULL!	0	2	0	0	3	0	0
#NULL!	2	0	2	2	3	2	4
#NULL!	2	3	4	2	0	4	2
#NULL!	3	3	2	3	0	2	2
#NULL!	2	2	4	2	0	4	2
#NULL!	3	3	3	3	2	3	0
#NULL!	3	3	3	3	2	3	0
#NULL!	3	3	3	3	2	3	0

#NULL!	3	3	3	3	2	3	0
#NULL!	3	3	2	3	2	2	0
#NULL!	3	3	2	3	2	2	0
#NULL!	4	2	4	4	3	4	2
#NULL!	4	2	4	4	0	4	2
#NULL!	4	2	4	4	3	4	2
#NULL!	4	2	4	4	0	4	2
#NULL!	4	2	4	4	3	4	2
#NULL!	4	2	4	4	0	4	2
#NULL!	4	2	2	4	3	2	2
#NULL!	4	2	4	4	0	4	2
#NULL!	4	2	2	4	3	2	2
#NULL!	4	2	4	4	0	4	2
#NULL!	3	4	3	3	2	3	2
#NULL!	3	4	3	3	2	3	2
#NULL!	3	4	3	3	2	3	2
#NULL!	3	4	3	3	2	3	2
#NULL!	3	4	3	3	2	3	2
#NULL!	4	0	4	4	3	4	3
#NULL!	4	0	4	4	3	4	3
#NULL!	4	0	4	4	3	4	3
#NULL!	4	0	4	4	3	4	3
#NULL!	4	0	2	4	3	2	3
#NULL!	4	0	2	4	3	2	3
#NULL!	3	4	3	3	0	3	2
#NULL!	3	4	3	3	0	3	2
#NULL!	2	3	2	2	0	2	2
#NULL!	3	4	3	3	0	3	2
#NULL!	3	4	3	3	0	3	2
#NULL!	2	3	2	2	0	2	2
#NULL!	3	4	4	3	0	4	2
#NULL!	2	3	2	2	0	2	2
#NULL!	3	4	4	3	0	4	2
#NULL!	2	2	2	2	0	2	0
#NULL!	2	2	2	2	0	2	0
#NULL!	2	2	2	2	0	2	0
#NULL!	2	2	2	2	0	2	0
#NULL!	2	2	2	2	0	2	0
#NULL!	2	2	2	2	3	2	0
#NULL!	2	2	2	2	3	2	0
#NULL!	2	2	2	2	3	2	0
#NULL!	2	2	2	2	3	2	0
#NULL!	2	2	2	2	3	2	0
#NULL!	2	2	2	2	2	2	2
#NULL!	3	4	3	3	4	3	0
#NULL!	4	2	4	4	2	4	3
#NULL!	4	0	4	4	2	4	0
#NULL!	2	2	2	2	2	2	2
#NULL!	3	4	3	3	4	3	0
#NULL!	2	2	2	2	2	2	2
#NULL!	4	2	4	4	2	4	3
#NULL!	4	0	4	4	2	4	0

#NULL!	4	2	4	4	2	4	4
#NULL!	2	2	2	2	2	2	2
#NULL!	3	4	3	3	4	3	0
#NULL!	2	2	2	2	2	2	2
#NULL!	4	2	4	4	2	4	3
#NULL!	4	0	4	4	2	4	0
#NULL!	2	2	2	2	2	2	2
#NULL!	3	4	3	3	4	3	0
#NULL!	2	2	2	2	2	2	2
#NULL!	4	2	4	4	2	4	3
#NULL!	4	0	2	4	2	2	0
#NULL!	4	2	4	4	2	4	4
#NULL!	2	2	2	2	2	2	2
#NULL!	3	4	3	3	4	3	0
#NULL!	2	2	2	2	2	2	2
#NULL!	4	2	4	4	2	4	3
#NULL!	4	0	2	4	2	2	0
#NULL!	4	2	4	4	2	4	4
#NULL!	2	2	2	2	2	2	2
#NULL!	3	4	3	3	4	3	0
#NULL!	2	2	2	2	2	2	2
#NULL!	4	2	4	4	2	4	3

Stress8	Stress9	Stress10	DEPRESSIO	DAAS1	DAAS2	DAAS3	DAAS4
2	2	2	#NULL!	0	0	2	2
0	2	0	#NULL!	2	2	0	2
3	3	3	#NULL!	2	2	3	2
3	4	2	#NULL!	2	0	3	2
2	2	0	#NULL!	0	0	2	0
2	2	4	#NULL!	2	2	2	0
2	2	1	#NULL!	2	2	2	2
2	2	2	#NULL!	0	2	2	0
2	2	0	#NULL!	2	2	2	3
2	4	2	#NULL!	2	2	2	2
4	2	4	#NULL!	0	2	4	0
4	0	2	#NULL!	2	2	4	0
4	2	4	#NULL!	2	2	4	2
4	3	4	#NULL!	0	2	4	0
4	4	4	#NULL!	3	2	4	2
4	4	4	#NULL!	3	3	4	2
4	3	4	#NULL!	2	2	4	0
1	3	3	#NULL!	2	0	1	0
4	3	4	#NULL!	2	2	4	2
4	3	4	#NULL!	0	2	4	2
4	4	0	#NULL!	3	3	4	2
2	0	2	#NULL!	0	2	2	2
3	2	3	#NULL!	2	0	3	0
2	2	2	#NULL!	3	3	2	3
4	2	4	#NULL!	2	0	4	0
3	2	3	#NULL!	2	2	3	3
4	2	4	#NULL!	2	2	4	2
2	4	4	#NULL!	3	3	2	3
4	2	4	#NULL!	0	2	4	2
2	2	2	#NULL!	2	2	2	2
2	2	2	#NULL!	2	2	2	2
0	3	0	#NULL!	3	2	0	3
4	3	4	#NULL!	2	0	4	0
3	2	2	#NULL!	2	3	3	2
4	4	4	#NULL!	3	2	4	2
2	2	2	#NULL!	2	2	2	2
4	2	4	#NULL!	2	2	4	3
2	2	2	#NULL!	0	0	2	0
2	3	2	#NULL!	2	2	2	2
3	2	3	#NULL!	2	2	3	2
4	3	4	#NULL!	2	2	4	2
3	4	3	#NULL!	3	2	3	3
2	0	2	#NULL!	3	2	2	2
2	0	2	#NULL!	2	2	2	2
3	3	3	#NULL!	3	2	3	0
2	4	3	#NULL!	3	3	2	2
2	0	2	#NULL!	0	2	2	2
4	2	4	#NULL!	3	2	4	0
4	2	4	#NULL!	0	0	4	2
3	4	2	#NULL!	0	0	3	3
4	2	0	#NULL!	2	2	0	0

4	0	2	#NULL!	0	0	4	2
4	4	4	#NULL!	3	3	4	2
2	2	2	#NULL!	2	2	2	3
2	0	2	#NULL!	0	2	2	2
2	3	2	#NULL!	2	2	2	3
2	4	2	#NULL!	3	3	2	2
2	2	2	#NULL!	0	0	2	2
3	2	3	#NULL!	2	2	3	2
4	2	4	#NULL!	0	2	4	2
2	4	2	#NULL!	2	3	2	0
2	4	2	#NULL!	3	3	2	2
0	2	0	#NULL!	2	2	0	2
4	2	4	#NULL!	2	2	4	0
2	2	1	#NULL!	2	2	2	2
4	3	4	#NULL!	2	2	4	2
2	0	2	#NULL!	3	2	2	2
2	4	2	#NULL!	3	3	2	2
0	2	0	#NULL!	2	2	0	2
2	2	2	#NULL!	2	2	2	0
2	2	2	#NULL!	2	2	2	2
4	3	4	#NULL!	2	2	4	2
2	0	2	#NULL!	3	2	2	2
2	4	2	#NULL!	3	3	2	2
0	2	0	#NULL!	2	2	0	2
2	2	4	#NULL!	2	2	2	0
4	2	2	#NULL!	2	2	4	2
4	3	4	#NULL!	2	2	4	2
2	0	2	#NULL!	3	2	2	2
2	4	3	#NULL!	3	3	2	2
0	2	0	#NULL!	2	2	0	2
4	2	4	#NULL!	2	2	4	0
2	2	1	#NULL!	2	2	2	2
4	3	4	#NULL!	2	2	4	2
2	0	2	#NULL!	3	2	2	2
2	4	2	#NULL!	3	3	2	2
0	2	0	#NULL!	2	2	0	2
2	2	4	#NULL!	2	2	2	0
2	2	4	#NULL!	2	2	2	2
4	3	4	#NULL!	2	2	4	2
2	0	2	#NULL!	3	2	2	2
2	4	2	#NULL!	3	3	2	2
0	2	0	#NULL!	2	2	0	2
4	2	4	#NULL!	2	2	4	0
3	2	2	#NULL!	2	2	3	2
3	3	3	#NULL!	3	2	3	0
2	2	2	#NULL!	2	2	2	3
2	3	3	#NULL!	3	2	2	0
2	2	2	#NULL!	2	2	2	3
3	3	3	#NULL!	3	2	3	0
2	2	2	#NULL!	2	2	2	3
2	3	3	#NULL!	3	2	2	0
2	2	2	#NULL!	2	2	2	3

2	3	3	#NULL!	3	2	2	0
2	2	2	#NULL!	2	2	2	3
3	2	3	#NULL!	2	2	3	2
4	0	2	#NULL!	0	0	4	2
3	2	3	#NULL!	2	2	3	2
4	0	4	#NULL!	0	0	4	2
2	2	3	#NULL!	2	2	2	2
4	0	4	#NULL!	0	0	4	2
2	2	3	#NULL!	2	2	2	2
4	0	4	#NULL!	0	0	4	2
2	2	3	#NULL!	2	2	2	2
4	0	4	#NULL!	0	0	4	2
2	3	2	#NULL!	2	2	2	2
2	0	2	#NULL!	0	2	2	2
2	3	2	#NULL!	2	2	2	2
2	0	2	#NULL!	0	2	2	2
4	3	2	#NULL!	2	2	4	2
4	0	2	#NULL!	0	2	4	2
4	3	2	#NULL!	2	2	4	2
4	0	2	#NULL!	0	2	4	2
4	3	2	#NULL!	2	2	4	2
4	0	2	#NULL!	0	2	4	2
3	3	3	#NULL!	0	2	3	2
2	0	2	#NULL!	2	2	2	2
3	3	3	#NULL!	0	2	3	2
2	0	2	#NULL!	2	2	2	2
3	3	3	#NULL!	0	2	3	2
4	0	2	#NULL!	2	2	4	2
4	3	3	#NULL!	0	2	4	2
4	0	2	#NULL!	2	2	4	2
4	3	3	#NULL!	0	2	4	2
4	0	2	#NULL!	2	2	4	2
4	3	3	#NULL!	0	2	4	2
2	2	0	#NULL!	2	2	2	3
2	4	2	#NULL!	2	2	2	2
2	0	3	#NULL!	2	2	2	0
4	4	4	#NULL!	3	3	4	2
1	3	3	#NULL!	2	0	1	0
4	3	4	#NULL!	2	2	4	2
3	4	4	#NULL!	3	3	3	2
3	2	3	#NULL!	2	0	3	0
2	2	2	#NULL!	3	3	2	3
4	2	4	#NULL!	2	0	4	0
2	2	4	#NULL!	0	0	2	2
2	2	2	#NULL!	2	2	2	2
4	2	2	#NULL!	2	2	4	2
0	3	0	#NULL!	3	2	0	3
4	3	4	#NULL!	2	0	4	0
2	2	2	#NULL!	2	2	2	2
3	4	3	#NULL!	0	0	3	3
4	4	4	#NULL!	3	3	4	2
2	4	2	#NULL!	3	3	2	2

2	2	0	#NULL!	2	2	2	3
2	4	2	#NULL!	2	2	2	2
4	0	2	#NULL!	2	2	4	0
4	4	4	#NULL!	3	3	4	2
1	3	1	#NULL!	2	0	1	0
4	3	4	#NULL!	2	2	4	2
4	4	0	#NULL!	3	3	4	2
3	2	3	#NULL!	2	0	3	0
2	2	2	#NULL!	3	3	2	3
4	2	4	#NULL!	2	0	4	0
4	2	4	#NULL!	0	2	4	2
2	2	2	#NULL!	2	2	2	2
2	2	2	#NULL!	2	2	2	2
0	3	0	#NULL!	3	2	0	3
4	3	4	#NULL!	2	0	4	0
2	2	2	#NULL!	2	2	2	2
3	4	3	#NULL!	0	0	3	3
4	4	4	#NULL!	3	3	4	2
2	4	2	#NULL!	3	3	2	2
3	2	3	#NULL!	2	2	3	2
4	2	4	#NULL!	0	2	4	2
2	2	0	#NULL!	2	2	2	3
2	4	2	#NULL!	2	2	2	2
4	0	4	#NULL!	2	2	4	0
3	4	4	#NULL!	3	3	3	2
1	3	1	#NULL!	2	0	1	0
2	3	4	#NULL!	2	2	2	2
3	4	4	#NULL!	3	3	3	2
4	2	3	#NULL!	2	0	4	0
3	2	2	#NULL!	3	3	3	3
4	2	4	#NULL!	2	0	4	0
2	2	4	#NULL!	0	0	0	2
4	2	2	#NULL!	2	2	4	2
4	2	2	#NULL!	2	2	4	2
0	3	0	#NULL!	3	2	0	3
2	3	4	#NULL!	2	0	2	0
2	2	2	#NULL!	2	2	2	2
2	4	3	#NULL!	0	0	2	3
2	4	4	#NULL!	3	3	2	2
2	2	0	#NULL!	2	2	2	3
2	4	2	#NULL!	2	2	2	2
4	0	2	#NULL!	2	2	4	0
3	4	4	#NULL!	3	3	3	2
1	3	1	#NULL!	2	0	1	0
2	3	4	#NULL!	2	2	2	2
4	4	0	#NULL!	3	3	4	2
4	2	3	#NULL!	2	0	4	0
3	2	2	#NULL!	3	3	3	3
4	2	4	#NULL!	2	0	4	0
4	2	4	#NULL!	0	3	4	2
4	2	2	#NULL!	2	2	4	2
2	2	2	#NULL!	2	2	2	2

0	3	0	#NULL!	3	2	0	3
2	3	4	#NULL!	2	0	2	0
2	2	2	#NULL!	2	2	2	2
2	4	3	#NULL!	0	0	2	3
2	4	4	#NULL!	3	3	2	2
3	4	2	#NULL!	3	3	3	2
3	2	3	#NULL!	2	2	3	2
4	2	4	#NULL!	0	2	4	2
2	2	0	#NULL!	2	2	2	3
2	4	2	#NULL!	2	2	2	2
4	0	4	#NULL!	2	2	4	0
3	4	4	#NULL!	3	3	3	2
1	3	1	#NULL!	2	0	1	0
2	3	4	#NULL!	2	2	2	2
3	4	3	#NULL!	3	3	3	2
4	2	3	#NULL!	2	0	4	0
3	2	2	#NULL!	3	3	3	3
4	2	4	#NULL!	2	0	4	0
2	2	4	#NULL!	0	4	0	2
4	2	2	#NULL!	2	2	4	2
4	2	2	#NULL!	2	2	4	2
0	3	0	#NULL!	3	2	0	3
2	3	4	#NULL!	2	0	2	0
2	2	2	#NULL!	2	2	2	2
2	4	3	#NULL!	0	0	2	3
2	4	4	#NULL!	3	3	2	2
3	4	2	#NULL!	3	3	3	2
2	2	3	#NULL!	2	2	2	2
2	2	4	#NULL!	0	2	2	2
2	2	0	#NULL!	2	2	2	3
2	4	2	#NULL!	2	2	2	2
4	0	4	#NULL!	2	2	4	0
3	4	3	#NULL!	3	3	3	2
1	3	1	#NULL!	2	0	1	0
2	3	2	#NULL!	2	2	2	2
3	4	4	#NULL!	3	3	3	2
4	2	4	#NULL!	2	0	4	0
3	2	3	#NULL!	3	3	3	3
4	2	4	#NULL!	2	0	4	0
3	4	2	#NULL!	2	0	3	2
4	4	4	#NULL!	3	2	4	2
2	4	3	#NULL!	2	0	2	2
4	4	4	#NULL!	3	2	4	2
2	4	3	#NULL!	2	0	2	2
2	4	4	#NULL!	3	2	2	2
2	4	3	#NULL!	2	0	2	2
2	4	4	#NULL!	3	2	2	2
2	4	2	#NULL!	2	0	2	2
2	4	4	#NULL!	3	2	2	2
2	4	2	#NULL!	2	0	2	2
4	2	4	#NULL!	0	2	4	0
4	2	4	#NULL!	0	2	4	0

4	2	4	#NULL!	0	2	4	0
4	2	4	#NULL!	0	2	4	0
4	2	4	#NULL!	0	2	4	0
4	2	4	#NULL!	0	2	4	0
2	2	2	#NULL!	0	2	2	0
4	3	4	#NULL!	2	2	4	0
4	2	2	#NULL!	0	2	4	0
4	3	4	#NULL!	2	2	4	0
2	2	2	#NULL!	0	2	2	0
4	3	4	#NULL!	2	2	4	0
4	2	4	#NULL!	0	2	4	0
4	3	4	#NULL!	2	2	4	0
4	2	2	#NULL!	0	2	4	0
4	3	4	#NULL!	2	2	4	0
4	2	2	#NULL!	0	2	4	0
4	3	4	#NULL!	2	2	4	0
4	2	4	#NULL!	3	2	4	0
4	2	4	#NULL!	3	2	4	0
3	2	4	#NULL!	3	2	3	0
3	2	4	#NULL!	3	2	3	0
3	2	4	#NULL!	3	2	3	0
4	2	0	#NULL!	2	2	0	0
4	2	0	#NULL!	2	2	0	0
4	2	0	#NULL!	2	2	3	0
4	2	0	#NULL!	2	2	3	0
1	2	0	#NULL!	2	2	1	0
4	2	4	#NULL!	2	2	4	2
4	2	4	#NULL!	2	2	4	2
4	2	4	#NULL!	2	2	4	3
2	3	2	#NULL!	2	2	2	3
4	2	4	#NULL!	2	2	4	2
0	2	4	#NULL!	2	2	0	2
4	2	4	#NULL!	2	2	4	3
2	3	2	#NULL!	2	2	2	3
2	2	4	#NULL!	2	2	2	2
0	2	4	#NULL!	2	2	0	2
2	2	4	#NULL!	2	2	2	3
2	3	2	#NULL!	2	2	2	3
2	2	4	#NULL!	2	2	2	2
0	2	4	#NULL!	2	2	0	2
2	2	4	#NULL!	2	2	2	3
2	3	2	#NULL!	2	2	2	3
2	4	2	#NULL!	3	3	2	2
2	2	4	#NULL!	2	2	2	2
0	2	0	#NULL!	2	2	0	2
2	2	4	#NULL!	2	2	2	3
2	3	2	#NULL!	2	2	2	3
2	4	2	#NULL!	3	3	2	2
2	2	2	#NULL!	2	2	2	2
4	4	4	#NULL!	3	2	4	2
4	4	4	#NULL!	3	2	4	2
3	4	4	#NULL!	3	2	3	2

3	4	4	#NULL!	3	2	3	2
3	4	4	#NULL!	3	2	3	2
3	4	3	#NULL!	3	2	3	2
2	2	2	#NULL!	0	0	2	0
2	0	2	#NULL!	0	0	2	2
2	2	2	#NULL!	0	0	2	0
2	0	2	#NULL!	0	0	2	2
4	2	2	#NULL!	0	0	4	0
4	0	2	#NULL!	0	0	4	2
4	2	2	#NULL!	0	0	4	0
4	0	2	#NULL!	0	0	4	2
4	2	2	#NULL!	0	0	4	0
4	0	2	#NULL!	0	0	4	2
3	4	3	#NULL!	3	2	3	3
3	4	3	#NULL!	3	2	3	3
3	4	3	#NULL!	3	2	3	3
3	4	3	#NULL!	3	2	3	3
3	4	3	#NULL!	3	2	3	3
4	3	4	#NULL!	0	2	4	0
4	3	4	#NULL!	0	2	4	0
4	3	4	#NULL!	0	2	4	0
4	3	4	#NULL!	0	2	4	0
4	3	4	#NULL!	0	2	4	0
4	3	4	#NULL!	0	2	4	0
3	2	3	#NULL!	2	2	3	3
3	2	3	#NULL!	2	2	3	3
2	4	2	#NULL!	2	3	2	0
3	2	3	#NULL!	2	2	3	3
3	2	3	#NULL!	2	2	3	3
2	4	2	#NULL!	2	3	2	0
3	2	3	#NULL!	2	2	3	3
2	4	2	#NULL!	2	3	2	0
3	2	3	#NULL!	2	2	3	3
2	2	2	#NULL!	2	3	2	2
2	2	2	#NULL!	2	3	2	2
2	2	2	#NULL!	2	3	2	2
2	2	2	#NULL!	2	3	2	2
2	2	2	#NULL!	2	3	2	2
2	4	4	#NULL!	3	3	2	3
2	4	2	#NULL!	3	3	2	3
2	4	2	#NULL!	3	3	2	3
2	4	2	#NULL!	3	3	2	3
2	4	2	#NULL!	3	3	2	3
2	2	0	#NULL!	0	0	2	2
3	3	3	#NULL!	2	2	3	2
2	0	2	#NULL!	0	2	2	2
4	2	4	#NULL!	0	0	4	2
2	2	0	#NULL!	0	0	2	2
3	3	3	#NULL!	2	2	3	2
2	2	0	#NULL!	0	0	2	0
2	0	2	#NULL!	0	2	2	2
4	2	4	#NULL!	0	0	4	2

2	2	2	#NULL!	0	0	2	2
2	2	0	#NULL!	0	0	2	2
3	3	3	#NULL!	2	2	3	2
2	2	2	#NULL!	0	0	0	0
4	0	2	#NULL!	0	2	4	2
4	2	4	#NULL!	0	0	4	2
2	2	0	#NULL!	0	0	2	2
3	3	3	#NULL!	2	2	3	2
2	2	2	#NULL!	0	0	0	0
4	0	2	#NULL!	0	2	4	2
4	2	4	#NULL!	0	0	4	2
2	2	2	#NULL!	0	0	2	2
2	2	0	#NULL!	0	0	2	2
3	3	3	#NULL!	2	2	3	2
2	2	0	#NULL!	0	0	2	0
4	0	2	#NULL!	0	2	4	2
4	2	4	#NULL!	0	0	4	2
4	2	2	#NULL!	0	0	4	2
2	2	0	#NULL!	0	0	2	2
3	3	3	#NULL!	2	2	3	2
2	2	2	#NULL!	0	0	0	0
4	0	4	#NULL!	0	2	4	2

DAAS5	DAAS6	DAAS7	DAAS8	DAAS9	DAAS10	DAAS11	DAAS12
2	4	4	3	4	2	4	4
0	0	4	4	4	2	2	2
3	3	3	3	4	2	0	0
2	2	2	2	2	3	2	2
2	0	2	3	4	0	4	3
4	4	2	2	2	0	2	2
1	1	1	3	0	2	2	0
2	2	3	3	3	0	3	4
2	0	2	3	4	2	4	4
2	2	4	4	4	2	2	2
4	4	4	4	4	2	2	2
2	2	1	1	1	2	1	1
4	4	4	4	4	2	4	4
4	2	2	2	2	2	2	2
4	4	2	2	2	2	2	2
4	4	2	2	2	2	2	1
4	4	2	2	2	2	3	3
3	0	0	2	2	0	2	2
4	4	4	2	2	2	2	2
4	3	3	3	3	3	3	3
0	0	0	0	0	2	4	0
2	4	4	4	4	0	4	4
3	2	2	2	4	2	4	4
2	4	4	4	3	3	3	3
4	2	2	2	4	2	4	4
3	3	3	3	3	2	3	3
4	2	2	2	2	2	2	3
4	4	2	2	2	2	2	4
4	4	4	4	4	4	4	4
2	2	2	2	2	2	2	4
2	3	3	2	2	2	2	2
0	0	0	0	4	3	4	4
4	4	4	4	2	2	2	2
2	2	2	2	2	2	2	2
4	3	3	3	2	3	2	2
2	2	2	2	2	2	3	3
4	2	2	2	2	0	2	2
2	2	2	4	4	2	4	4
2	4	4	4	4	0	4	4
3	4	4	2	2	2	2	2
4	4	4	4	4	2	4	2
3	4	0	0	0	2	4	0
2	4	4	4	3	0	3	3
2	4	4	4	2	2	2	2
3	3	4	4	4	0	4	4
3	3	3	4	4	3	4	4
2	4	4	4	4	2	4	4
4	2	2	2	4	3	4	4
4	2	2	2	4	2	4	4
2	2	2	2	3	2	2	2
2	2	0	3	0	2	0	2

2	2	2	2	2	2	2	2
4	3	3	3	3	0	3	3
2	2	3	3	4	2	4	4
2	2	2	4	4	2	4	4
2	4	4	2	2	3	2	2
2	2	3	2	2	2	2	2
2	2	2	2	2	2	3	3
3	4	4	4	4	2	4	2
4	2	2	2	4	2	4	4
2	2	2	3	3	3	3	3
2	2	2	2	2	2	2	3
0	4	4	4	2	2	2	2
4	2	2	2	2	0	2	2
1	1	1	3	0	2	0	0
4	4	4	4	4	2	2	2
2	4	4	4	3	0	3	3
2	3	3	3	4	3	4	4
0	0	0	0	4	2	4	4
2	4	4	2	2	0	2	2
2	1	1	1	1	0	3	0
4	4	4	4	2	2	2	2
2	2	4	4	4	0	3	3
2	2	3	3	3	3	4	4
0	0	0	4	4	2	4	2
4	4	2	2	2	0	2	2
2	1	1	1	3	3	0	0
4	4	4	4	4	2	4	4
2	2	4	4	4	0	3	3
3	3	3	4	4	3	4	4
0	0	4	4	2	2	2	2
4	2	2	2	2	0	2	2
1	1	1	1	3	0	0	0
4	4	4	4	4	2	2	2
2	2	4	4	4	0	4	3
2	2	3	3	3	3	4	4
0	0	0	4	4	2	4	4
4	4	4	2	2	0	2	2
4	2	1	1	1	2	1	3
4	4	4	4	4	2	4	4
2	2	2	4	4	0	4	4
2	3	3	3	4	3	4	4
0	0	4	4	4	2	2	2
4	4	2	2	2	0	2	2
2	2	1	1	1	0	3	3
3	3	4	4	4	0	4	4
2	2	3	3	3	2	4	4
3	3	3	4	4	0	4	4
2	2	3	3	3	2	4	4
3	4	4	4	4	0	4	2
2	2	2	3	3	2	3	4
3	3	4	4	4	0	4	4
2	2	2	3	3	2	3	3

3	3	3	4	4	0	4	4
2	2	2	2	3	2	3	3
3	4	4	4	2	2	2	2
2	2	2	2	2	2	2	2
3	3	4	4	2	2	2	2
4	2	2	2	2	2	2	2
3	3	4	4	4	2	2	2
4	2	2	2	2	2	2	2
3	3	4	4	4	2	2	2
4	4	2	2	2	2	2	2
3	3	3	4	4	2	4	2
4	4	2	2	2	2	2	2
2	2	4	4	4	0	4	4
2	2	4	4	4	2	4	4
2	2	4	4	4	0	4	4
2	2	4	4	4	2	4	4
2	2	4	4	4	0	4	4
2	2	4	4	4	2	4	4
2	2	2	4	4	0	4	4
2	2	2	4	4	2	4	4
2	2	2	4	4	0	4	4
2	2	2	4	4	2	4	4
3	3	3	3	3	2	3	3
2	2	4	4	2	2	2	2
3	3	3	3	3	2	3	3
2	2	4	4	4	2	2	2
3	3	3	3	3	2	3	3
2	2	4	4	4	2	2	2
3	3	3	3	3	2	3	3
2	2	2	4	4	2	4	2
3	3	3	3	3	2	3	3
2	2	2	4	4	2	4	4
3	3	3	3	3	2	3	3
2	0	4	3	3	2	4	4
2	2	2	4	4	2	4	2
3	4	2	1	1	2	1	1
4	4	4	3	4	2	2	2
3	0	0	2	2	0	2	2
4	4	4	4	2	2	2	2
4	0	0	0	0	2	4	0
3	3	2	2	4	2	4	4
2	4	4	4	3	3	3	3
4	4	2	2	4	2	4	4
4	4	4	3	4	0	4	4
2	2	2	2	2	2	2	2
2	2	3	3	2	2	2	2
0	0	0	0	0	3	4	4
4	4	4	4	2	2	2	2
2	2	2	2	2	2	3	3
3	2	2	2	3	2	3	2
4	4	3	3	3	0	3	3
2	2	3	2	2	2	2	2

2	0	4	3	3	2	4	4
2	2	2	4	4	2	4	4
2	2	2	1	1	2	1	1
4	4	2	2	2	2	2	2
1	3	0	2	2	0	2	2
4	4	4	4	2	2	2	2
0	0	0	0	0	2	4	0
3	3	2	2	2	2	4	4
2	2	4	4	4	3	3	3
4	4	2	2	2	2	4	4
4	4	4	4	4	2	4	4
2	2	2	2	2	2	2	2
2	3	3	2	2	2	2	2
0	0	0	0	0	3	4	4
4	4	4	4	4	2	2	2
2	3	3	4	4	2	4	4
3	2	2	2	2	2	3	3
4	4	3	3	3	0	3	3
2	2	3	3	2	2	2	2
3	3	4	4	4	2	2	4
4	2	2	2	4	2	4	4
2	0	3	3	4	2	4	4
2	2	2	2	4	2	4	4
4	3	2	2	1	2	1	1
4	4	4	4	3	2	4	2
1	3	0	2	2	0	2	2
4	4	4	4	4	2	2	2
4	4	0	0	0	2	4	0
3	3	2	2	2	2	4	4
2	2	4	4	4	3	3	3
4	4	2	2	2	2	4	4
2	4	4	3	4	0	4	4
2	2	2	2	2	2	2	2
2	2	2	3	3	2	2	2
0	0	0	0	0	3	4	4
4	4	4	4	4	2	2	2
2	3	3	3	4	2	4	4
3	3	2	2	2	2	3	3
4	4	3	3	3	0	3	3
2	0	4	3	4	2	4	4
2	2	2	2	4	2	4	4
2	2	4	2	1	2	1	1
4	4	4	2	2	2	2	4
1	1	0	0	2	0	2	2
4	4	4	4	4	2	2	2
0	0	0	0	0	2	4	0
3	3	3	2	2	2	2	4
2	2	4	4	4	3	4	3
4	4	4	2	2	2	2	4
4	4	4	4	4	3	4	4
2	2	2	2	2	2	2	2
2	2	3	3	2	2	2	2

0	0	0	0	0	3	4	0
4	4	4	4	4	2	4	2
2	2	2	2	3	2	3	3
3	3	2	2	2	2	2	3
4	4	4	3	3	0	3	3
2	2	2	3	2	2	2	2
3	3	4	4	4	2	4	2
4	4	2	2	2	2	4	4
2	0	3	3	4	2	4	3
2	2	2	2	2	2	4	4
4	4	2	2	2	2	1	1
4	4	4	4	4	2	3	2
1	1	3	0	2	0	2	2
4	4	4	4	4	2	4	2
3	4	0	0	0	2	4	0
3	3	3	2	2	2	2	2
2	2	2	4	4	3	4	4
4	4	4	2	2	2	2	2
4	4	4	3	4	0	4	4
2	2	2	2	2	2	2	2
2	2	3	3	3	2	2	2
0	0	0	0	0	3	4	0
4	4	4	4	4	2	4	4
2	2	2	2	2	2	3	3
3	3	2	2	2	2	2	2
4	4	4	3	3	0	3	3
2	2	2	3	3	2	2	2
3	3	4	4	4	2	4	4
4	4	2	2	2	2	4	4
2	0	4	3	3	2	4	4
2	2	2	2	2	2	4	4
4	2	3	4	2	2	1	1
3	4	4	4	2	2	2	3
1	1	3	0	0	0	2	2
2	4	4	4	4	2	4	4
4	0	0	0	0	2	4	0
4	3	3	3	2	2	2	2
3	2	2	4	4	3	4	4
4	4	4	4	2	2	2	2
2	2	2	2	2	3	2	2
4	4	3	3	3	3	2	2
3	2	2	2	2	3	2	2
4	4	3	3	3	3	2	2
3	2	2	2	2	3	2	2
4	4	3	3	3	3	3	2
3	3	2	2	2	3	2	2
4	4	4	3	3	3	3	3
2	3	2	2	2	3	2	2
4	4	4	3	3	3	3	3
2	3	2	2	2	3	2	2
4	4	4	4	4	2	4	4
4	4	4	4	4	2	2	2

4	4	4	4	4	2	4	4
4	4	4	4	4	2	2	2
4	4	4	4	4	2	2	2
4	4	4	4	4	2	4	2
2	2	2	3	3	0	3	3
4	4	4	2	2	2	2	2
2	2	2	2	3	0	3	3
4	4	4	2	2	2	2	3
2	2	2	3	3	0	3	3
4	2	2	3	3	2	3	2
4	2	2	2	2	0	3	3
4	2	2	2	3	2	3	3
2	2	2	2	3	0	3	3
4	4	2	2	3	2	3	3
2	2	2	2	2	0	3	3
4	4	2	2	2	2	3	3
4	2	2	2	4	3	4	4
4	4	2	2	2	3	4	4
4	4	2	2	2	3	4	4
4	4	2	2	2	3	2	4
4	4	4	2	2	3	2	2
2	2	0	3	0	2	0	2
2	2	0	3	0	2	0	2
0	2	0	3	0	2	0	2
0	2	0	3	0	2	0	2
0	0	0	3	0	2	0	2
4	4	4	4	4	2	4	4
4	4	2	2	2	2	2	2
4	2	2	2	2	0	2	2
2	2	4	4	4	3	2	2
4	4	4	4	4	2	4	4
4	4	2	2	2	2	2	2
4	4	2	2	2	0	2	2
2	4	4	4	2	3	2	2
4	4	4	4	4	2	4	4
4	4	2	2	2	2	2	2
4	4	2	2	2	0	2	2
2	2	4	4	4	3	2	2
4	4	4	4	4	2	4	4
4	4	4	2	2	2	2	2
4	4	2	2	2	0	2	2
2	2	4	4	2	3	2	2
2	2	2	2	2	2	2	2
4	4	4	4	4	2	4	4
0	4	4	2	2	2	2	2
4	4	4	2	2	0	2	2
2	2	2	4	4	3	4	2
2	2	2	2	2	2	2	2
2	4	4	4	4	2	4	4
4	4	4	2	2	2	2	2
4	4	4	2	2	2	2	2
4	4	4	4	2	2	2	2

4	4	4	4	2	2	2	2
4	4	4	4	4	2	2	2
3	4	4	4	4	2	2	2
2	2	2	2	4	2	4	4
2	2	2	2	4	2	4	4
2	2	2	2	4	2	4	4
2	2	2	2	4	2	4	4
2	2	2	2	2	2	4	4
2	2	2	2	2	2	4	4
2	2	2	2	2	2	4	4
2	2	2	2	2	2	4	4
2	2	2	2	2	2	2	4
2	2	2	2	2	2	2	4
3	3	4	0	0	2	4	0
3	3	4	0	0	2	4	0
3	3	4	4	0	2	4	0
3	3	3	4	0	2	4	0
3	3	3	4	4	2	4	0
4	2	2	2	2	2	2	2
4	4	2	2	2	2	2	2
4	4	2	2	2	2	2	2
4	4	2	2	2	2	2	2
4	4	4	2	2	2	2	2
4	4	4	2	2	2	2	2
3	3	3	3	3	2	3	3
3	3	3	3	3	2	3	3
2	2	2	3	3	3	3	3
3	3	3	3	3	2	3	3
3	3	3	3	3	2	3	3
2	2	2	2	3	3	3	3
3	3	3	3	3	2	3	3
2	2	2	2	3	3	3	3
3	3	3	3	3	2	3	3
2	3	3	3	4	2	4	4
2	3	3	3	4	2	4	4
2	2	3	3	3	2	4	4
2	2	3	3	3	2	4	4
2	2	3	3	3	2	3	4
4	4	4	2	2	2	2	2
2	4	4	2	2	2	2	2
2	4	4	4	2	2	2	2
2	2	4	4	2	2	2	2
2	2	4	4	4	2	2	2
2	4	4	3	4	2	4	4
3	3	3	3	4	2	4	0
2	4	4	4	4	0	4	4
4	2	2	2	4	2	4	4
2	2	4	3	4	2	4	4
3	3	3	3	3	2	4	4
2	0	3	3	4	0	4	3
2	2	4	4	4	0	4	4
4	4	2	2	2	2	4	4

2	2	2	2	2	2	2	3
2	0	4	3	4	2	4	4
3	3	3	3	3	2	4	4
2	2	0	0	4	0	4	2
2	2	4	4	4	0	4	4
4	4	2	2	2	2	4	4
2	0	4	3	34	2	4	4
3	3	3	3	3	2	3	4
2	0	3	3	4	0	4	2
2	2	4	4	4	0	4	4
4	4	2	2	2	2	2	4
2	2	2	2	2	2	2	2
2	0	4	3	3	2	4	4
3	3	3	3	3	2	3	3
0	0	2	3	4	0	4	3
2	2	2	4	4	0	4	4
4	4	4	2	2	2	2	2
2	2	2	2	2	2	2	2
2	0	4	3	3	2	4	4
3	3	3	3	3	2	3	3
2	0	2	3	4	0	4	2
4	2	2	4	4	0	4	4

DAAS13	DAAS14	DAAS15	DAAS16	DAAS17	DAAS18	DAAS19	DAAS20
4	3	4	3	3	4	3	2
2	2	2	0	0	2	0	3
0	3	0	2	2	0	2	0
2	3	2	3	3	3	2	3
2	0	4	0	0	4	0	4
2	2	2	0	0	2	0	4
2	2	0	2	0	0	2	2
4	0	4	0	0	4	0	4
4	2	4	2	2	4	2	4
2	2	2	2	2	4	0	4
3	0	3	0	2	2	3	2
3	2	0	2	0	0	2	2
4	0	2	0	2	2	2	4
4	0	4	2	2	2	0	2
2	2	2	2	3	2	2	3
1	2	1	0	2	1	2	3
3	2	2	0	0	2	2	2
2	0	0	0	0	3	0	2
4	2	4	2	0	4	0	2
3	0	3	2	0	3	0	3
0	2	4	3	3	4	2	2
2	2	2	0	0	2	0	2
4	0	3	2	3	3	0	3
3	3	3	2	3	3	3	3
4	2	4	2	2	4	2	2
3	2	4	2	2	0	2	0
3	2	3	2	0	4	2	4
4	2	4	2	3	4	2	4
4	2	2	0	2	2	3	3
4	2	4	2	2	2	2	2
3	2	3	2	2	2	2	2
4	3	2	3	3	2	3	2
2	2	2	2	2	2	3	4
2	0	3	2	2	3	2	3
2	3	4	3	2	4	2	4
3	3	3	2	2	4	2	4
4	2	4	0	2	4	2	3
3	0	3	2	2	3	2	2
4	2	4	0	0	4	0	4
4	3	4	2	2	4	3	4
2	2	2	2	2	4	2	4
0	3	0	0	0	0	2	0
3	0	2	0	2	2	2	4
2	2	2	2	2	2	2	2
2	2	2	2	2	2	2	2
2	3	4	3	2	4	2	2
2	0	2	0	2	2	2	4
4	0	4	2	3	4	3	2
2	2	2	2	0	2	0	2
2	0	2	2	3	2	2	2
2	2	2	2	0	0	0	3

2	0	3	2	0	3	0	4
3	3	3	2	3	3	2	3
4	2	2	2	2	2	2	2
2	2	2	2	0	2	2	2
2	2	2	2	0	4	2	4
2	3	2	2	3	2	3	2
3	2	3	0	0	4	2	4
2	0	4	0	2	1	2	1
4	0	4	0	2	4	2	4
4	3	4	3	3	4	3	4
3	2	3	3	2	4	3	4
2	2	2	0	0	3	0	3
2	2	2	0	0	4	0	4
2	2	0	0	0	0	2	0
2	2	2	2	2	4	2	4
3	0	3	0	2	2	2	2
4	3	4	3	2	2	2	2
2	2	2	0	0	2	0	2
2	2	2	0	0	2	0	2
0	2	3	0	0	2	2	0
2	2	4	2	2	4	2	4
3	0	3	0	2	2	2	2
4	3	4	3	2	4	2	3
2	2	2	0	0	2	0	2
2	2	2	0	0	2	0	2
2	2	3	0	0	0	2	0
2	2	2	2	2	4	2	4
3	0	3	0	2	2	2	2
4	3	2	3	2	2	2	2
2	2	2	0	0	3	0	3
2	2	2	0	0	4	0	4
2	2	0	0	0	0	2	0
2	2	2	2	2	4	2	4
3	0	3	0	2	2	2	2
4	3	4	3	2	2	2	2
2	2	2	0	0	2	0	2
2	2	2	0	0	2	0	2
0	2	0	0	0	2	2	0
2	2	2	2	2	2	2	4
3	0	3	0	2	3	2	2
4	3	4	3	2	3	2	4
2	2	2	0	0	2	0	3
2	2	2	0	0	2	0	4
0	2	0	0	0	3	2	0
2	2	2	2	2	2	2	2
4	2	4	2	2	2	2	2
4	2	4	2	2	2	2	2
4	2	4	2	2	2	2	2
2	2	2	2	2	2	2	2
4	2	4	2	2	2	2	2
4	2	2	2	2	2	2	2
4	2	4	2	2	2	2	2

4	2	4	2	2	2	2	2
4	2	4	2	2	4	2	2
2	3	4	2	2	4	3	4
2	0	2	2	0	3	0	3
2	3	2	2	2	4	3	4
2	0	2	2	0	3	0	3
2	3	2	2	2	4	3	4
2	0	2	2	0	3	0	3
2	3	2	2	2	4	3	4
2	0	2	2	0	3	0	3
2	3	2	2	2	4	3	4
2	0	2	2	0	2	0	3
4	2	4	0	0	4	0	4
4	0	2	0	2	2	2	2
4	2	4	0	0	4	0	4
4	0	4	0	2	2	2	2
4	2	4	0	0	4	0	4
4	0	4	0	2	2	2	2
4	2	4	0	0	4	0	4
4	0	4	0	2	2	2	2
4	2	4	0	0	4	0	4
4	0	4	0	2	2	2	2
3	0	3	2	0	3	0	3
2	2	2	2	2	2	2	2
3	0	3	2	0	3	0	3
2	2	2	2	2	2	2	2
3	0	3	2	0	3	0	3
2	2	2	2	2	2	2	2
3	0	3	2	0	3	0	3
2	2	2	2	2	2	2	2
3	0	3	2	0	3	0	3
2	2	2	2	2	2	2	2
3	0	3	2	0	3	0	3
4	2	4	2	2	4	2	4
2	2	2	2	2	4	0	4
3	2	3	2	0	0	2	2
1	2	1	0	2	1	2	3
2	0	0	0	0	3	2	2
2	2	4	2	0	4	0	2
0	2	0	3	3	4	2	4
4	0	4	2	3	3	0	3
3	3	3	2	3	3	3	3
4	2	4	2	2	4	2	4
4	2	4	2	2	2	3	2
4	2	4	2	2	2	2	2
2	2	2	2	2	3	2	2
4	3	4	3	3	2	3	2
2	2	2	2	2	2	3	4
3	3	3	2	2	4	2	4
2	0	2	2	3	2	2	2
3	3	3	2	3	3	2	3
2	3	2	2	3	2	3	2

4	2	4	2	2	4	2	4
2	2	2	2	2	2	0	4
1	2	3	2	0	0	2	2
1	2	1	0	2	1	2	1
2	0	0	0	0	3	0	2
2	2	2	2	0	4	0	4
0	2	0	3	3	4	2	4
4	0	4	2	3	3	0	3
3	3	3	2	3	3	3	3
4	2	4	2	2	4	2	4
4	2	4	0	2	2	3	2
4	2	4	2	2	4	2	2
2	2	3	2	2	2	2	2
4	3	4	3	3	2	3	2
2	2	2	2	2	2	3	2
2	3	2	2	2	2	2	4
2	0	2	2	3	2	2	2
3	3	3	2	3	3	2	3
2	3	2	2	3	2	3	2
3	0	2	0	2	2	2	1
4	0	4	0	2	4	2	4
4	2	4	2	2	4	2	4
2	2	2	2	2	2	0	4
1	2	1	2	0	0	2	2
2	2	1	0	2	1	2	1
2	0	0	0	0	3	3	2
2	2	2	2	0	4	0	4
0	2	0	3	3	0	2	4
4	0	4	2	3	3	0	3
3	3	3	2	3	3	3	3
4	2	4	2	2	4	2	4
4	2	4	3	2	4	3	2
2	2	4	2	2	4	2	2
2	2	2	2	2	3	2	3
4	3	4	3	3	2	3	2
2	2	2	2	2	2	3	2
2	3	2	2	2	2	2	4
2	0	2	2	3	2	2	2
3	3	3	2	3	3	2	3
4	2	4	2	2	4	2	4
4	2	2	2	2	2	0	2
1	2	1	2	0	0	2	0
2	2	2	0	2	1	2	1
2	0	0	0	0	3	0	2
2	2	2	2	0	4	0	4
0	2	0	3	3	4	2	4
4	0	4	2	3	3	0	3
3	3	3	2	3	3	3	3
4	2	4	2	2	4	2	4
4	2	4	0	2	2	3	2
2	2	2	2	2	4	2	4
2	2	2	2	2	2	2	2

4	3	4	3	3	2	3	2
2	2	2	2	2	2	3	2
3	3	4	2	2	4	2	2
3	0	2	2	3	2	2	2
3	3	3	2	3	3	2	3
2	3	2	2	3	2	3	2
2	0	3	0	2	2	2	1
4	0	4	0	2	4	2	4
4	2	4	2	2	4	2	4
4	2	4	2	2	2	0	2
1	2	1	2	0	3	2	0
2	2	2	0	2	1	2	1
2	0	0	0	0	3	2	2
2	2	2	2	0	4	0	4
0	2	0	3	3	0	2	4
4	0	4	2	3	4	0	3
3	3	3	2	3	3	3	3
4	2	4	2	2	4	2	4
4	2	4	2	2	2	3	2
2	2	2	2	2	4	2	4
2	2	2	2	2	3	2	2
4	3	4	3	3	4	3	2
2	2	2	2	2	2	3	2
3	3	3	2	2	4	2	4
3	0	3	2	3	2	2	2
3	3	3	2	3	3	2	3
2	3	2	2	3	2	3	2
4	0	2	0	2	2	2	2
4	0	4	0	2	4	2	4
4	2	4	2	2	4	2	4
4	2	4	2	2	2	0	2
1	2	1	2	0	3	2	0
4	2	2	0	2	1	2	1
2	0	0	0	0	3	0	2
2	2	2	2	0	2	0	4
0	2	0	3	3	4	2	4
4	0	4	2	3	4	0	3
3	3	3	2	3	3	3	3
4	2	4	2	2	4	2	4
2	3	2	3	3	3	2	3
2	3	2	3	2	4	2	4
2	3	2	3	3	3	2	3
2	3	2	3	2	4	2	4
2	3	2	3	3	3	2	3
2	3	2	3	2	4	2	4
2	3	2	3	3	2	2	3
2	3	2	3	2	4	2	4
2	3	2	3	3	2	2	3
2	3	2	3	2	2	2	4
2	3	2	3	3	2	2	2
2	2	2	3	2	3	0	3
2	0	3	0	2	3	3	2

2	3	2	3	2	2	0	3
2	0	2	0	2	3	4	2
2	2	2	4	2	3	3	3
2	0	2	3	2	3	2	3
4	0	4	0	0	4	0	2
3	2	3	0	0	2	2	2
3	0	3	0	0	4	0	4
3	2	3	0	0	2	2	2
4	0	4	0	0	4	0	4
2	2	2	0	0	2	2	3
3	0	3	0	0	4	0	4
2	2	2	0	0	2	2	3
3	0	4	0	0	4	0	4
2	2	2	0	0	2	2	2
3	0	3	0	0	4	0	4
3	2	3	0	0	2	2	2
4	0	4	2	3	4	3	2
4	0	4	2	3	4	3	4
4	0	4	2	3	4	3	4
4	0	4	2	3	4	3	4
4	0	4	2	3	4	3	4
2	2	2	2	0	0	0	0
2	2	2	2	0	0	0	3
2	2	2	2	0	0	0	0
2	2	2	2	0	0	0	4
2	2	2	2	0	0	0	3
4	0	4	0	2	2	2	2
3	2	3	2	0	3	2	4
4	2	4	0	2	4	2	3
2	2	2	2	0	2	2	2
4	0	4	0	2	2	2	2
3	2	3	2	0	3	2	4
2	2	4	0	2	4	2	4
2	2	2	2	0	2	2	4
4	0	4	0	2	2	2	2
2	2	3	2	0	3	2	3
2	2	2	0	2	4	2	4
2	2	2	2	0	2	2	2
4	0	4	0	2	2	2	2
2	2	2	2	0	3	2	3
2	2	2	0	2	4	2	4
2	2	2	2	0	2	2	4
3	2	3	3	2	3	3	4
4	0	4	0	2	4	2	2
2	2	2	2	0	3	2	3
2	2	2	0	2	4	2	4
2	2	2	2	0	2	2	2
3	2	3	3	2	3	3	4
4	0	4	0	2	4	2	2
2	2	2	2	3	2	2	2
2	2	2	2	3	2	2	2
2	2	2	2	3	2	2	2

2	2	2	2	3	2	2	2
2	2	2	2	3	2	2	2
2	2	2	2	3	2	2	2
4	0	3	2	2	3	2	3
2	2	2	2	0	2	2	2
4	0	4	2	2	3	2	3
4	2	2	2	0	2	2	2
4	0	4	2	2	3	2	3
4	2	4	2	0	2	2	2
4	0	4	2	2	3	2	3
4	2	4	2	0	2	2	2
4	0	4	2	2	3	2	3
4	2	4	2	0	2	2	2
0	3	0	0	0	0	2	0
0	3	0	0	0	0	2	0
0	3	0	0	0	0	2	0
0	3	0	0	0	0	2	0
0	3	0	0	0	0	2	0
4	0	4	2	2	4	0	2
2	0	4	2	2	4	0	2
2	0	2	2	2	4	0	4
2	0	2	2	2	4	0	4
2	0	2	2	2	4	0	4
2	0	2	2	2	4	0	4
3	2	3	2	2	0	2	0
3	2	3	2	2	4	2	0
4	3	4	3	3	4	3	4
3	2	3	2	2	4	2	0
3	2	3	2	2	4	2	4
3	3	4	3	3	4	3	4
3	2	3	2	2	3	2	4
3	3	3	3	3	4	3	4
3	2	3	2	2	3	2	4
4	0	4	2	2	2	2	2
4	0	4	2	2	2	2	2
4	0	4	2	2	4	2	2
4	0	4	2	2	4	2	2
4	0	4	2	2	4	2	4
4	2	4	2	3	4	2	4
4	2	4	2	3	4	2	4
2	2	4	2	3	4	2	4
2	2	2	2	3	4	2	4
2	2	2	2	3	4	2	4
4	3	4	3	3	4	3	4
0	3	0	2	2	0	2	0
4	2	2	0	0	2	0	2
4	2	2	2	0	2	0	2
4	3	4	3	3	4	3	4
0	3	0	2	2	0	2	0
0	0	2	0	0	4	0	4
4	2	4	0	0	2	0	2
4	2	4	2	0	2	0	2

3	2	3	0	0	4	2	4
4	3	4	3	3	4	3	4
0	3	0	2	2	0	2	0
0	0	0	0	0	2	0	4
4	2	4	0	0	2	0	2
4	2	4	2	0	2	0	2
4	3	4	3	3	4	3	4
4	3	0	2	2	0	2	0
0	0	0	0	0	4	0	4
4	2	4	0	0	2	0	2
4	2	4	2	0	2	0	2
3	2	3	0	0	4	2	4
4	3	4	3	3	4	3	4
4	3	4	2	2	0	2	0
0	0	0	0	0	4	0	4
4	2	4	0	0	2	0	2
4	2	4	2	0	2	0	2
3	2	3	0	0	3	2	4
4	3	4	3	3	4	3	4
4	3	4	2	2	0	2	0
0	0	0	0	0	4	0	4
4	2	4	0	0	4	0	2

DAAS21	ANXIETY	DAAS22	DAAS23	DAAS24	DAAS25	DAAS26	DAAS27
3	#NULL!	2	0	4	2	3	3
0	#NULL!	2	0	2	0	0	4
2	#NULL!	2	3	0	3	2	4
3	#NULL!	2	0	3	2	3	4
0	#NULL!	2	0	4	0	0	4
0	#NULL!	2	0	2	0	0	2
3	0.00	0	0	0	0	2	0
0	#NULL!	2	0	4	2	2	2
2	#NULL!	2	2	4	2	2	4
2	#NULL!	0	0	4	2	2	4
2	#NULL!	2	2	3	0	2	3
0	#NULL!	2	3	0	0	2	0
2	#NULL!	0	2	2	2	2	4
0	#NULL!	0	2	4	2	2	2
3	#NULL!	0	2	2	3	2	4
3	#NULL!	2	3	1	2	3	3
4	0.00	2	2	2	2	2	2
0	#NULL!	2	0	2	2	2	2
2	#NULL!	2	0	4	0	2	2
0	#NULL!	2	3	3	0	2	4
2	#NULL!	3	2	4	2	3	2
0	#NULL!	0	0	2	2	2	4
0	#NULL!	2	0	3	0	2	3
2	#NULL!	3	3	3	2	2	3
2	#NULL!	2	2	4	0	0	2
2	#NULL!	2	2	0	2	2	0
0	#NULL!	2	2	3	2	2	4
3	#NULL!	2	2	4	2	2	4
3	#NULL!	3	2	2	2	0	2
2	#NULL!	2	2	2	2	2	4
2	#NULL!	2	0	2	2	2	2
3	#NULL!	3	2	2	3	3	3
3	#NULL!	2	3	2	2	2	2
3	#NULL!	2	2	3	2	2	4
0	#NULL!	0	0	4	2	3	3
3	#NULL!	2	2	4	2	2	2
2	#NULL!	0	2	4	0	0	2
0	#NULL!	0	2	3	2	0	4
2	#NULL!	2	2	4	2	0	4
2	#NULL!	2	2	4	3	2	2
2	#NULL!	0	0	4	2	0	4
3	#NULL!	2	2	0	2	2	2
0	#NULL!	2	2	2	2	0	3
3	#NULL!	2	0	2	2	2	4
3	#NULL!	0	0	2	2	2	2
2	#NULL!	3	2	2	3	2	1
2	#NULL!	2	2	2	2	2	2
2	#NULL!	2	2	4	2	2	4
2	#NULL!	0	2	2	0	2	2
2	#NULL!	3	3	2	3	3	3
2	#NULL!	2	0	3	0	2	2

0	#NULL!	2	2	3	2	2	2
0	#NULL!	3	3	3	2	2	3
2	#NULL!	2	3	2	3	2	4
2	#NULL!	0	2	2	2	0	4
0	#NULL!	0	2	2	0	2	3
3	#NULL!	2	2	2	0	3	3
0	#NULL!	2	2	4	0	2	2
2	#NULL!	0	2	2	2	2	3
2	#NULL!	0	0	4	2	2	2
2	#NULL!	2	3	4	2	2	2
2	#NULL!	3	2	3	2	3	4
0	#NULL!	2	0	3	0	0	4
0	#NULL!	2	0	4	0	0	2
3	0.00	0	0	3	0	2	0
2	#NULL!	0	0	4	2	0	4
0	#NULL!	2	2	2	2	0	4
2	#NULL!	3	2	4	3	2	1
0	#NULL!	2	0	2	0	0	3
0	#NULL!	2	0	2	0	0	4
0	#NULL!	0	0	0	0	2	0
2	#NULL!	0	0	4	2	0	2
0	#NULL!	2	2	2	2	0	4
2	#NULL!	3	2	2	3	2	1
0	#NULL!	2	0	2	0	0	3
0	#NULL!	2	0	2	0	0	2
0	3.00	0	0	3	0	2	0
2	#NULL!	0	0	2	2	0	4
0	#NULL!	2	2	2	2	0	4
2	#NULL!	3	2	3	3	2	1
0	#NULL!	2	0	2	0	0	4
0	#NULL!	2	0	2	0	0	2
0	3.00	0	0	0	0	2	0
2	#NULL!	0	0	4	2	0	4
0	#NULL!	2	2	3	2	0	4
2	#NULL!	3	2	4	3	2	1
0	#NULL!	2	0	2	0	0	3
0	#NULL!	2	0	2	0	0	4
0	#NULL!	0	3	3	0	2	0
2	#NULL!	0	0	2	2	0	4
0	#NULL!	2	2	3	2	0	4
2	#NULL!	3	2	2	3	2	1
0	#NULL!	2	0	2	0	0	4
0	#NULL!	2	0	2	0	0	2
0	#NULL!	0	0	0	0	2	0
3	#NULL!	0	0	2	2	2	2
2	#NULL!	2	3	2	3	2	4
3	#NULL!	0	0	2	2	2	2
2	#NULL!	2	3	2	3	2	4
3	#NULL!	0	0	2	2	2	3
2	#NULL!	2	3	2	3	2	4
3	#NULL!	0	0	2	2	2	2
2	#NULL!	2	3	4	3	2	4

3	#NULL!	0	0	2	2	2	2
2	#NULL!	2	3	4	3	2	4
2	#NULL!	2	2	4	3	2	2
0	#NULL!	2	2	3	2	2	2
2	#NULL!	2	2	4	3	2	2
0	#NULL!	2	2	3	2	2	4
2	#NULL!	2	2	4	3	2	4
0	#NULL!	2	2	3	2	2	4
2	#NULL!	2	2	4	3	2	4
0	#NULL!	2	2	2	2	2	4
2	#NULL!	2	2	2	3	2	4
0	#NULL!	2	2	2	2	2	4
2	#NULL!	2	2	4	2	0	2
2	#NULL!	2	2	2	2	2	2
2	#NULL!	2	2	4	2	0	2
2	#NULL!	2	2	2	2	2	4
2	#NULL!	2	2	4	2	0	2
2	#NULL!	2	2	2	2	2	4
2	#NULL!	2	2	4	2	0	2
2	#NULL!	2	2	2	2	2	4
2	#NULL!	2	2	4	2	0	2
2	#NULL!	2	2	4	2	2	4
0	#NULL!	2	3	3	0	2	0
3	#NULL!	2	0	2	2	2	4
0	#NULL!	2	3	3	0	2	0
3	#NULL!	2	0	2	2	2	4
0	#NULL!	2	3	3	0	2	0
3	#NULL!	2	0	2	2	2	4
0	#NULL!	2	3	3	0	2	0
3	#NULL!	2	0	2	2	2	2
0	#NULL!	2	3	3	0	2	4
3	#NULL!	2	0	2	2	2	2
0	#NULL!	2	3	3	0	2	4
2	#NULL!	2	2	4	2	2	4
2	#NULL!	0	0	2	2	2	4
0	#NULL!	2	3	0	0	2	0
3	#NULL!	2	3	1	2	3	3
0	#NULL!	2	0	2	2	2	2
2	#NULL!	2	0	4	0	2	2
2	#NULL!	3	2	0	2	3	2
0	#NULL!	2	0	3	0	2	3
2	#NULL!	3	3	3	2	2	3
2	#NULL!	2	2	4	0	0	2
3	#NULL!	3	2	2	2	0	3
2	#NULL!	2	2	4	2	2	4
2	#NULL!	2	2	3	2	2	2
3	#NULL!	3	2	2	3	3	3
3	#NULL!	2	3	2	2	2	2
3	#NULL!	2	2	4	2	2	2
2	#NULL!	3	3	2	3	3	2
0	#NULL!	3	3	3	2	2	3
3	#NULL!	2	2	2	0	3	3

2	#NULL!	2	2	4	2	2	4
2	#NULL!	0	0	2	2	2	4
0	#NULL!	2	3	0	0	2	0
3	#NULL!	2	3	1	2	3	3
0	#NULL!	2	0	2	2	2	2
2	#NULL!	2	0	4	0	2	2
2	#NULL!	3	2	4	2	3	2
0	#NULL!	2	0	3	0	2	3
2	#NULL!	3	3	3	2	2	3
2	#NULL!	2	2	4	0	0	2
3	#NULL!	3	2	2	2	0	2
2	#NULL!	2	2	4	2	2	4
2	#NULL!	2	0	2	2	2	2
3	#NULL!	3	2	2	3	3	3
3	#NULL!	2	3	2	2	2	2
3	#NULL!	2	2	2	2	2	4
2	#NULL!	3	3	2	3	3	2
0	#NULL!	3	3	3	2	2	3
3	#NULL!	2	2	2	0	3	3
2	#NULL!	0	2	2	2	2	3
2	#NULL!	0	0	4	2	2	4
2	#NULL!	2	2	4	2	2	4
2	#NULL!	0	0	2	2	2	4
0	#NULL!	2	3	0	0	2	0
3	#NULL!	2	3	1	2	3	3
0	#NULL!	2	0	2	2	2	2
2	#NULL!	2	0	4	0	2	2
2	#NULL!	3	2	0	2	3	2
0	#NULL!	2	0	3	0	2	3
2	#NULL!	3	3	3	2	2	3
2	#NULL!	2	2	4	0	0	2
3	#NULL!	3	2	4	2	0	3
2	#NULL!	2	2	4	2	2	4
2	#NULL!	2	3	2	2	2	2
3	#NULL!	3	2	2	3	3	3
3	#NULL!	2	3	2	2	2	4
3	#NULL!	2	2	2	2	2	4
2	#NULL!	3	3	2	3	3	2
0	#NULL!	3	3	3	2	2	3
2	#NULL!	2	2	4	2	2	4
2	#NULL!	0	0	2	2	2	4
0	#NULL!	2	3	3	0	2	0
3	#NULL!	2	3	1	2	3	0
0	#NULL!	2	0	2	2	2	2
2	#NULL!	2	0	4	0	2	2
2	#NULL!	3	2	4	2	3	2
0	#NULL!	2	0	4	0	2	3
2	#NULL!	3	3	3	2	2	3
2	#NULL!	2	2	4	0	0	2
3	#NULL!	3	2	2	2	0	2
2	#NULL!	2	2	4	2	2	2
2	#NULL!	2	0	3	2	2	2

3	#NULL!	3	2	4	3	3	2
3	#NULL!	2	3	2	2	2	4
3	#NULL!	2	2	4	2	2	4
2	#NULL!	3	3	2	3	3	2
0	#NULL!	3	3	3	2	2	3
3	#NULL!	2	2	2	0	3	3
2	#NULL!	0	2	2	2	2	1
2	#NULL!	0	0	4	2	2	4
2	#NULL!	2	2	4	2	2	4
2	#NULL!	0	0	2	2	2	4
0	#NULL!	2	3	3	0	2	0
3	#NULL!	2	3	1	2	3	0
0	#NULL!	2	0	2	2	2	2
2	#NULL!	2	0	2	0	2	2
2	#NULL!	3	2	0	2	3	2
0	#NULL!	2	0	4	0	2	3
2	#NULL!	3	3	3	2	2	3
2	#NULL!	2	2	4	0	0	2
3	#NULL!	3	2	4	2	0	3
2	#NULL!	2	2	4	2	2	2
2	#NULL!	2	2	3	2	2	2
3	#NULL!	3	2	4	3	3	2
3	#NULL!	2	3	2	2	2	4
3	#NULL!	2	2	4	2	2	2
2	#NULL!	3	3	2	3	3	2
0	#NULL!	3	3	3	2	2	3
3	#NULL!	2	2	2	0	3	3
2	#NULL!	0	2	4	2	2	1
2	#NULL!	0	0	4	2	2	4
2	#NULL!	2	2	4	2	2	2
2	#NULL!	0	0	2	2	2	4
0	#NULL!	2	3	1	0	2	0
3	#NULL!	2	3	1	2	3	0
0	#NULL!	2	0	2	2	2	2
2	#NULL!	2	0	2	0	2	2
2	#NULL!	3	2	4	2	3	2
0	#NULL!	2	0	4	0	2	3
2	#NULL!	3	3	3	2	2	3
2	#NULL!	2	2	4	0	0	2
3	#NULL!	2	0	3	2	3	4
0	#NULL!	0	0	4	2	3	3
3	#NULL!	2	0	3	2	3	4
0	#NULL!	0	0	4	2	3	3
3	#NULL!	2	0	2	2	3	4
0	#NULL!	0	0	4	2	3	3
3	#NULL!	2	0	2	2	3	4
0	#NULL!	0	0	2	2	3	3
3	#NULL!	2	0	2	2	3	4
0	#NULL!	0	0	2	2	3	3
3	#NULL!	2	0	2	2	3	3
2	#NULL!	2	2	2	0	2	2
2	#NULL!	2	2	3	0	2	3

2	#NULL!	2	2	2	0	2	2
2	#NULL!	2	2	3	0	2	2
2	#NULL!	2	2	3	0	2	2
2	#NULL!	2	2	3	0	2	2
2	0.00	2	0	4	2	2	2
3	4.00	2	2	2	2	2	3
0	2.00	2	0	4	2	2	2
2	3.00	2	2	2	2	2	2
3	0.00	2	0	4	2	2	2
0	#NULL!	2	2	2	2	2	2
0	3.00	2	0	4	2	2	2
0	#NULL!	2	2	2	2	2	2
2	0.00	2	0	4	2	2	4
0	#NULL!	2	2	2	2	2	2
0	2.00	2	0	4	2	2	3
0	2.00	2	2	2	2	2	2
2	#NULL!	2	2	4	2	2	4
2	#NULL!	2	2	4	2	2	2
2	#NULL!	2	2	4	2	2	2
2	#NULL!	2	2	4	2	2	2
2	#NULL!	2	2	4	2	2	2
2	#NULL!	2	0	3	0	2	2
2	#NULL!	2	0	3	0	2	2
2	#NULL!	2	0	3	0	2	2
2	#NULL!	2	0	3	0	2	2
2	#NULL!	2	0	3	0	2	2
2	#NULL!	0	2	2	2	2	4
0	#NULL!	2	2	3	2	2	2
2	#NULL!	0	2	4	0	0	2
0	#NULL!	0	2	2	0	2	4
2	#NULL!	0	2	2	2	2	4
0	#NULL!	2	2	3	2	2	4
2	#NULL!	0	2	4	0	0	3
0	#NULL!	0	2	2	0	2	3
2	#NULL!	0	2	2	2	2	4
0	#NULL!	2	2	3	2	2	4
2	#NULL!	0	2	4	0	0	3
0	#NULL!	0	2	2	0	2	4
2	#NULL!	0	2	4	2	2	4
0	#NULL!	2	2	3	2	2	4
2	#NULL!	0	2	4	0	0	3
0	#NULL!	0	2	2	0	2	3
2	#NULL!	3	2	3	2	3	4
2	#NULL!	0	2	4	2	2	4
0	#NULL!	2	2	3	2	2	4
2	#NULL!	0	2	4	0	0	3
0	#NULL!	0	2	2	0	2	4
2	#NULL!	3	2	3	2	3	4
2	#NULL!	0	2	4	2	2	4
3	#NULL!	0	2	2	3	2	4
3	#NULL!	0	2	2	3	2	3
3	#NULL!	0	2	2	3	2	3

3	#NULL!	0	2	2	3	2	3
3	#NULL!	0	2	2	3	2	3
3	#NULL!	0	2	2	3	2	3
0	#NULL!	0	2	3	2	0	4
2	#NULL!	0	2	2	2	0	4
0	#NULL!	0	2	3	2	0	2
2	#NULL!	0	2	2	2	0	4
0	#NULL!	0	2	3	2	0	2
2	#NULL!	0	2	2	2	0	4
0	#NULL!	0	2	3	2	0	2
2	#NULL!	0	2	2	2	0	2
0	#NULL!	0	2	4	2	0	2
2	#NULL!	0	2	2	2	0	2
3	#NULL!	2	2	0	2	2	4
3	#NULL!	2	2	0	2	2	4
3	#NULL!	2	2	0	2	2	4
3	#NULL!	2	2	0	2	2	4
3	#NULL!	2	2	0	2	2	4
0	#NULL!	0	2	4	2	2	2
0	#NULL!	0	2	4	2	2	2
0	#NULL!	0	2	4	2	2	2
0	#NULL!	0	2	4	2	2	2
0	#NULL!	0	2	4	2	2	2
0	#NULL!	0	2	2	2	2	2
2	#NULL!	2	2	4	2	2	0
2	#NULL!	2	2	4	2	2	0
2	#NULL!	2	3	4	2	2	2
2	#NULL!	2	2	4	2	2	0
2	#NULL!	2	2	3	2	2	0
2	#NULL!	2	3	4	2	2	2
2	#NULL!	2	2	3	2	2	0
2	#NULL!	2	3	4	2	2	2
2	#NULL!	2	2	3	2	2	0
3	#NULL!	2	2	2	2	2	2
3	#NULL!	2	2	4	2	2	2
3	#NULL!	2	2	4	2	2	2
3	#NULL!	2	2	4	2	2	2
3	#NULL!	2	2	4	2	2	2
3	#NULL!	2	2	4	2	2	4
3	#NULL!	2	2	4	2	2	4
3	#NULL!	2	2	4	2	2	4
3	#NULL!	2	2	4	2	2	4
3	#NULL!	2	2	4	2	2	4
3	#NULL!	2	0	4	2	3	3
2	#NULL!	2	3	0	3	2	0
0	#NULL!	0	0	2	2	2	4
2	#NULL!	0	2	2	0	2	2
3	#NULL!	2	0	4	2	3	2
2	#NULL!	2	3	0	3	2	0
0	#NULL!	2	0	4	0	0	4
0	#NULL!	0	0	2	2	2	4
2	#NULL!	0	2	2	0	2	2

0	#NULL!	2	2	4	0	2	2
3	#NULL!	2	0	4	2	3	2
2	#NULL!	2	3	0	3	2	0
0	#NULL!	2	0	0	0	0	4
0	#NULL!	0	0	2	2	2	2
2	#NULL!	0	2	2	0	2	2
3	#NULL!	2	0	4	2	3	2
2	#NULL!	2	3	0	3	2	0
0	#NULL!	2	0	4	0	0	4
0	#NULL!	0	0	2	2	2	2
2	#NULL!	0	2	2	0	2	2
0	#NULL!	2	2	3	0	2	2
3	#NULL!	2	0	4	2	3	2
2	#NULL!	2	3	0	3	2	0
0	#NULL!	2	0	4	0	0	4
0	#NULL!	0	0	4	2	2	2
2	#NULL!	0	2	4	0	2	2
0	#NULL!	2	2	3	0	2	2
3	#NULL!	2	0	4	2	3	2
2	#NULL!	2	3	0	3	2	0
0	#NULL!	2	0	2	0	0	4
0	#NULL!	0	0	4	2	2	2

DAAS28	DAAS29	DAAS30	DAAS31	DAAS32	DAAS33	DAAS34	DAAS35
2	3	2	2	2	2	2	3
0	2	0	4	0	3	0	0
3	2	3	2	3	0	3	3
3	2	2	2	3	4	2	2
0	0	0	2	3	4	0	0
0	0	0	4	0	4	0	2
0	0	0	0	0	2	0	0
2	2	2	1	2	2	2	2
3	2	2	2	2	4	2	3
2	2	2	4	0	4	2	0
0	2	2	2	2	2	2	2
0	2	0	2	0	2	2	2
2	0	2	2	2	4	2	0
0	0	2	4	0	2	0	0
2	3	3	2	3	3	2	2
3	3	2	3	2	0	2	3
0	2	0	2	0	3	0	0
0	0	0	0	0	0	0	0
0	0	0	2	0	2	0	0
2	0	2	0	2	3	0	2
3	3	0	3	2	2	3	3
0	2	0	2	0	2	0	2
0	2	2	3	0	3	0	2
2	2	3	4	2	3	3	3
2	2	0	4	2	2	2	0
3	3	2	4	2	0	3	2
2	2	0	2	2	4	2	0
2	2	2	2	2	4	2	0
2	2	2	3	2	3	0	2
2	2	2	4	2	2	3	2
2	2	2	3	2	2	2	2
3	3	2	4	3	2	3	3
3	2	2	2	2	4	3	2
0	2	3	2	3	4	0	0
3	2	2	3	3	3	3	2
2	2	3	2	2	4	2	3
0	0	2	4	2	3	2	0
2	2	0	3	2	2	2	2
2	0	2	4	2	2	2	2
2	3	2	4	3	4	0	2
3	3	2	2	2	4	2	0
2	3	2	2	3	4	2	2
0	3	3	3	0	4	2	2
2	2	2	2	0	2	2	2
3	2	2	4	0	2	0	2
2	3	0	0	2	1	3	3
2	0	0	2	0	4	0	2
2	3	2	2	0	2	2	2
2	0	2	4	0	2	3	0
3	2	2	4	2	2	3	2
0	3	0	2	2	2	2	2

2	2	2	4	3	4	2	0
2	3	0	0	2	3	2	2
0	2	2	2	3	4	2	3
0	0	2	3	0	2	2	2
3	2	2	2	0	4	2	0
2	2	2	4	3	3	2	2
2	0	2	4	0	2	2	2
3	0	0	0	2	1	0	2
2	2	0	4	0	4	2	0
3	2	2	2	2	2	3	2
3	2	3	2	2	4	3	3
0	2	0	2	0	3	0	0
0	0	0	4	4	2	0	2
0	0	0	0	2	2	0	0
3	3	2	2	2	4	2	0
0	3	3	3	0	4	2	2
2	3	0	3	2	2	3	3
0	2	0	4	0	3	0	0
0	0	0	2	0	4	0	2
0	0	0	0	0	2	0	0
3	3	2	2	2	4	2	0
0	3	3	3	0	4	2	2
2	3	0	3	2	2	3	3
0	2	0	4	0	3	0	0
0	0	0	4	0	4	0	2
0	0	0	0	3	2	0	0
3	3	2	2	2	4	2	0
0	3	3	3	0	4	2	2
2	3	0	0	2	1	3	3
0	2	0	4	0	3	0	0
0	0	0	4	3	4	0	2
0	0	0	0	0	2	0	0
3	3	2	2	2	4	2	0
0	3	3	3	0	2	2	2
2	3	0	1	2	4	3	3
0	2	0	4	0	3	0	0
0	0	0	2	0	4	0	2
0	0	0	0	0	2	0	0
3	3	2	2	2	4	2	0
0	3	3	3	0	2	2	2
2	3	0	0	2	2	3	3
0	2	0	2	0	3	0	0
0	0	0	4	2	4	0	2
0	0	0	0	2	2	0	0
3	2	2	3	0	2	0	2
0	2	2	2	3	4	2	3
3	2	2	3	0	2	0	2
0	2	2	4	3	4	2	3
3	2	2	4	0	2	0	2
0	2	2	4	3	2	2	3
3	2	2	3	0	2	0	2
0	2	2	4	3	2	2	3

3	2	2	3	0	2	0	2
0	2	2	4	3	2	2	3
2	3	2	4	3	4	0	2
2	2	2	4	3	4	2	0
2	3	2	4	3	4	0	2
2	2	2	2	3	4	2	0
2	3	2	2	3	4	0	2
2	2	2	2	3	4	2	0
2	3	2	2	3	4	0	2
2	2	2	2	3	3	2	0
2	3	2	2	3	4	0	2
2	2	2	2	3	3	2	0
2	0	2	4	2	2	2	2
2	0	0	2	0	4	0	2
2	0	2	4	2	2	2	2
2	0	0	2	0	4	0	2
2	0	2	4	2	4	2	2
2	0	0	2	0	4	0	2
2	0	2	4	2	4	2	2
2	0	0	2	0	2	0	2
2	0	2	4	2	4	2	2
2	0	0	2	0	2	0	2
2	0	2	0	2	4	0	2
2	2	2	2	0	2	2	2
2	0	2	0	2	4	0	2
2	2	2	2	0	2	2	2
2	0	2	0	2	4	0	2
2	2	2	2	0	2	2	2
2	0	2	0	2	3	0	2
2	2	2	2	0	2	2	2
2	0	2	0	2	3	0	2
2	2	2	4	0	2	2	2
2	0	2	0	2	3	0	2
2	2	2	3	2	4	2	3
2	2	2	4	0	4	2	0
0	2	0	2	0	2	2	2
3	3	2	3	2	0	2	3
0	0	0	0	0	0	0	0
0	0	0	2	0	2	0	0
3	3	0	3	2	4	3	3
0	2	2	3	0	3	0	2
2	2	3	3	2	3	3	3
2	2	0	4	2	2	2	0
2	2	2	2	2	3	0	2
2	2	2	4	2	2	3	2
2	2	2	2	2	2	2	2
3	3	2	4	3	2	3	3
3	2	2	2	2	4	3	2
2	2	3	2	2	4	2	3
3	2	2	3	2	2	3	2
2	3	0	0	2	3	2	2
2	2	2	4	3	3	2	2

0	2	2	2	2	4	2	3
2	2	2	4	0	4	2	0
0	2	0	2	0	2	2	2
3	3	2	3	2	0	2	3
0	0	0	0	0	0	0	0
0	0	0	2	0	2	0	0
3	3	0	3	2	2	3	3
0	2	2	3	0	3	0	2
2	2	3	3	2	3	3	3
2	2	0	4	2	2	2	0
2	2	2	3	2	3	0	2
2	2	2	4	2	2	3	2
2	2	2	3	2	2	2	2
3	3	2	4	3	2	3	3
3	2	2	2	2	4	3	2
2	2	3	2	2	4	2	3
3	2	2	3	2	2	3	2
2	3	0	0	2	3	2	2
2	2	2	4	3	2	2	2
3	0	0	0	2	1	0	2
2	2	0	4	0	4	2	0
3	2	2	2	2	4	2	3
2	2	2	4	0	4	2	0
0	2	0	2	0	2	2	2
3	3	2	3	2	3	2	3
0	0	0	0	0	0	0	0
0	0	0	2	0	2	0	0
3	3	0	2	2	4	3	3
0	2	2	3	0	3	0	2
2	2	3	3	2	3	3	3
2	2	0	2	2	2	2	0
2	2	2	2	2	2	0	2
2	2	2	4	2	2	3	2
2	2	2	2	2	2	2	2
3	3	2	4	3	2	3	3
3	2	2	2	2	4	3	2
2	2	3	2	2	4	2	3
3	2	2	3	2	2	3	2
2	3	0	0	2	3	2	2
3	2	2	2	2	4	2	3
2	2	2	4	0	4	2	0
0	2	0	2	0	2	2	2
3	3	2	3	2	3	2	3
0	0	0	0	0	0	0	0
0	0	0	2	0	2	0	0
3	3	0	3	2	2	3	3
0	2	2	3	0	3	0	2
2	2	3	3	2	3	3	3
2	2	0	2	2	4	2	0
2	2	2	2	2	3	0	2
2	2	2	4	2	2	3	2
2	2	2	2	2	2	2	2

3	3	2	3	3	2	3	3
3	2	2	2	2	4	3	2
2	2	3	4	2	2	2	3
3	2	2	3	2	2	3	2
2	3	0	4	2	3	2	2
2	2	2	4	3	2	2	2
3	0	0	0	2	1	0	2
2	2	0	2	0	4	2	0
2	2	2	2	2	4	2	3
2	2	2	4	0	4	2	0
0	2	0	2	0	2	2	2
3	3	2	3	2	1	2	3
0	0	0	0	0	0	0	0
0	0	0	2	0	4	0	0
3	3	0	2	2	4	3	3
0	2	2	3	0	3	0	2
2	2	3	3	2	3	3	3
2	2	0	2	2	4	2	0
2	2	2	2	2	2	0	2
2	2	2	4	2	2	3	2
2	2	2	2	2	2	2	2
3	3	2	3	3	2	3	3
3	2	2	2	2	2	3	2
2	2	3	4	2	2	2	3
3	2	2	3	2	2	3	2
2	3	0	4	2	3	2	2
2	2	2	4	3	2	2	2
3	0	0	0	2	1	0	2
2	2	0	2	0	4	2	0
0	2	2	3	2	4	2	3
2	2	2	4	0	4	2	0
0	2	0	2	0	2	2	2
3	3	2	3	2	1	2	3
0	0	0	0	0	0	0	0
0	0	0	2	0	4	0	0
3	3	0	3	2	2	3	3
0	2	2	3	0	3	0	2
2	2	3	3	2	3	3	3
2	2	0	2	2	4	2	0
3	2	2	2	3	4	2	2
3	2	2	3	3	3	3	2
3	2	2	2	3	3	2	2
3	2	2	3	3	3	3	2
3	2	2	4	3	3	2	2
3	2	2	3	3	4	3	2
3	2	2	4	3	3	2	2
3	2	2	3	3	4	3	2
3	2	2	4	3	3	2	2
3	2	2	3	3	4	3	2
3	2	2	4	3	3	2	2
0	2	2	2	2	2	2	2
2	2	2	2	2	2	2	2

4	2	2	3	2	3	2	2
0	2	2	2	2	2	2	2
3	2	2	2	2	2	2	2
0	2	2	2	2	2	2	2
2	2	2	1	2	3	2	2
0	2	0	2	0	2	0	0
2	2	2	1	2	2	2	2
0	2	0	2	0	2	0	0
2	2	2	1	2	2	2	2
0	2	0	2	0	2	0	0
2	2	2	1	2	4	2	2
0	2	0	2	0	2	0	0
2	2	2	1	2	4	2	2
0	2	0	2	0	3	0	0
2	2	2	1	2	4	2	2
0	2	0	2	0	3	0	0
2	3	2	2	0	2	2	2
2	3	2	2	0	2	2	2
2	3	2	4	0	2	2	2
2	3	2	4	0	2	2	2
2	3	2	4	0	4	2	2
0	3	0	2	2	2	2	2
0	3	0	2	2	2	2	2
0	3	0	2	2	2	2	2
0	3	0	2	2	2	2	2
0	3	0	2	2	2	2	2
2	0	2	2	2	4	2	0
2	2	0	2	2	4	2	0
0	0	2	4	2	3	2	0
3	2	2	3	0	4	2	0
2	0	2	4	2	4	2	0
2	2	0	2	2	4	2	0
0	0	2	4	2	3	2	0
3	2	2	2	0	4	2	0
2	0	2	4	2	4	2	0
2	2	0	4	2	4	2	0
0	0	2	2	2	3	2	0
3	2	2	3	0	4	2	0
2	0	2	4	2	2	2	0
2	2	0	2	2	4	2	0
0	0	2	2	2	3	2	0
3	2	2	2	0	4	2	0
3	2	3	2	2	4	3	3
2	0	2	4	2	2	2	0
2	2	0	3	2	4	2	0
0	0	2	2	2	4	2	0
3	2	2	3	0	4	2	0
3	2	3	2	2	4	3	3
2	0	2	4	2	2	2	0
2	3	3	2	3	3	2	2
2	3	3	2	3	3	2	2
2	3	3	4	3	3	2	2

2	3	3	4	3	2	2	2
2	3	3	4	3	2	2	2
2	3	3	4	3	2	2	2
2	2	0	3	2	2	2	2
0	0	2	3	0	2	2	2
2	2	0	4	2	2	2	2
0	0	2	3	0	2	2	2
2	2	0	4	2	2	2	2
0	0	2	3	0	2	2	2
2	2	0	4	2	3	2	2
0	0	2	4	0	2	2	2
2	2	0	4	2	3	2	2
0	0	2	4	0	2	2	2
2	3	2	2	3	4	2	2
2	3	2	2	3	4	2	2
2	3	2	2	3	0	2	2
2	3	2	2	3	0	2	2
2	3	2	2	3	0	2	2
0	0	2	4	0	2	0	0
0	0	2	4	0	2	0	0
0	0	2	4	0	2	0	0
0	0	2	4	0	2	0	0
0	0	2	2	0	2	0	0
0	0	2	2	0	4	0	0
3	3	2	0	2	0	3	2
3	3	2	0	2	0	3	2
3	2	2	2	2	2	3	2
3	3	2	0	2	0	3	2
3	3	2	0	2	0	3	2
3	2	2	2	2	4	3	2
3	3	2	0	2	0	3	2
3	2	2	2	2	4	3	2
3	3	2	0	2	0	3	2
0	2	3	3	3	2	0	0
0	2	3	3	3	2	0	0
0	2	3	2	3	2	0	0
0	2	3	2	3	2	0	0
0	2	3	2	3	2	0	0
2	2	2	2	2	4	2	0
2	2	2	2	2	4	2	0
2	2	2	2	2	4	2	0
2	2	2	4	2	4	2	0
2	2	2	4	2	4	2	0
2	3	2	2	2	2	2	3
3	2	3	2	3	0	3	3
0	2	0	2	0	2	0	2
2	0	2	4	0	2	3	0
2	3	2	2	2	2	2	3
3	2	3	4	3	0	3	3
0	0	0	4	0	4	0	0
0	2	0	2	0	2	0	2
2	0	2	4	0	2	3	0

2	0	2	4	0	2	2	2
2	3	2	2	2	2	2	3
3	2	3	4	3	0	3	3
0	0	0	4	0	4	0	0
0	2	0	2	0	2	0	2
2	0	2	4	0	2	3	0
2	3	2	3	2	4	2	3
3	2	3	4	3	0	3	3
0	0	0	4	0	4	0	0
0	2	0	4	0	2	0	2
2	0	2	4	0	2	3	0
2	0	2	4	0	4	2	2
2	3	2	3	2	4	2	3
3	2	3	4	3	0	3	3
0	0	0	4	3	4	0	0
0	2	0	4	0	2	0	2
2	0	2	2	0	2	3	0
2	0	2	4	0	4	2	2
2	3	2	3	2	4	2	3
3	2	3	4	3	0	3	3
0	0	0	4	0	4	0	0
0	2	0	4	0	2	0	2

DAAS236	DAAS37	DAAS38	DAAS39	DAAS40	DAAS41	DAAS42	MSPSS
2	3	3	2	0	0	2	#NULL!
0	0	0	0	2	2	2	#NULL!
3	2	3	3	2	3	2	#NULL!
3	3	3	3	2	2	2	#NULL!
0	0	3	0	0	2	2	#NULL!
0	0	0	0	2	2	2	#NULL!
0	2	0	2	2	0	0	#NULL!
2	2	2	2	2	3	2	#NULL!
2	2	2	3	2	2	2	#NULL!
2	2	3	2	2	2	2	#NULL!
2	2	2	2	2	2	0	#NULL!
2	2	2	0	2	0	2	#NULL!
2	0	2	2	0	2	0	#NULL!
0	2	2	2	0	2	2	#NULL!
0	0	2	2	3	3	3	#NULL!
3	3	3	2	3	0	0	#NULL!
0	0	0	0	0	2	0	#NULL!
4	0	3	2	0	0	3	#NULL!
0	2	2	2	0	0	0	#NULL!
2	0	0	2	2	2	2	#NULL!
2	2	3	3	2	0	2	#NULL!
2	2	2	2	0	2	0	#NULL!
2	2	2	2	0	2	0	#NULL!
2	3	2	2	2	2	3	#NULL!
2	2	2	0	2	2	2	#NULL!
2	3	2	2	2	3	3	#NULL!
2	3	2	0	2	0	2	#NULL!
0	0	2	2	2	3	3	#NULL!
0	2	2	2	0	2	2	#NULL!
2	2	2	2	2	2	2	#NULL!
2	2	2	2	2	2	2	#NULL!
3	2	2	3	3	3	3	#NULL!
3	2	3	2	3	2	3	#NULL!
2	2	2	2	2	3	2	#NULL!
3	2	3	2	0	2	2	#NULL!
3	3	2	3	2	2	2	#NULL!
2	2	2	2	2	0	0	#NULL!
0	2	2	2	2	2	0	#NULL!
2	2	2	2	2	2	2	#NULL!
2	2	3	2	3	2	3	#NULL!
0	0	2	0	2	3	3	#NULL!
2	3	3	2	3	2	3	#NULL!
2	2	2	2	0	2	0	#NULL!
3	3	2	2	2	2	2	#NULL!
0	2	0	2	2	2	2	#NULL!
2	2	2	3	2	2	0	#NULL!
2	2	0	2	0	2	2	#NULL!
2	2	2	2	3	3	2	#NULL!
0	2	0	2	0	0	0	#NULL!
2	2	2	3	3	0	0	#NULL!
2	2	0	0	0	2	0	#NULL!

0	2	0	2	2	2	2	#NULL!
2	3	3	3	2	2	2	#NULL!
2	2	0	2	3	2	3	#NULL!
2	0	2	2	0	2	0	#NULL!
2	2	0	2	2	2	2	#NULL!
3	2	3	2	2	3	3	#NULL!
2	2	0	0	2	2	2	#NULL!
2	2	2	0	2	0	2	#NULL!
2	2	2	0	2	0	0	#NULL!
3	2	3	2	2	2	2	#NULL!
3	2	0	2	2	2	3	#NULL!
0	0	0	0	2	2	2	#NULL!
0	0	0	0	2	2	2	#NULL!
0	2	2	2	2	0	0	#NULL!
0	0	2	0	2	3	3	#NULL!
2	2	2	2	0	2	0	#NULL!
2	2	2	3	2	2	0	#NULL!
0	0	0	0	2	2	2	#NULL!
0	0	0	0	2	2	2	#NULL!
0	2	0	2	2	0	0	#NULL!
0	0	2	0	2	3	3	#NULL!
2	2	2	2	0	2	0	#NULL!
2	2	2	3	2	2	0	#NULL!
0	0	0	0	2	2	2	#NULL!
0	0	0	0	2	2	2	#NULL!
0	2	3	2	2	0	0	#NULL!
0	0	2	0	2	3	3	#NULL!
2	2	2	2	0	2	0	#NULL!
2	2	2	3	2	2	0	#NULL!
0	0	0	0	2	2	2	#NULL!
0	0	0	0	2	2	2	#NULL!
0	2	0	2	2	0	0	#NULL!
0	0	2	0	2	3	3	#NULL!
2	2	2	2	0	2	0	#NULL!
2	2	2	3	2	2	0	#NULL!
0	0	0	0	2	2	2	#NULL!
0	0	0	0	2	2	2	#NULL!
0	2	0	2	2	0	0	#NULL!
0	0	2	0	2	3	3	#NULL!
2	2	2	2	0	2	0	#NULL!
2	2	2	3	2	2	0	#NULL!
0	0	0	0	2	2	2	#NULL!
0	0	0	0	2	2	2	#NULL!
0	2	2	2	2	0	0	#NULL!
0	2	0	2	2	2	2	#NULL!
2	2	0	2	3	2	3	#NULL!
0	2	0	2	2	2	2	#NULL!
2	2	0	2	3	2	3	#NULL!
0	2	0	2	2	2	2	#NULL!
2	2	0	2	3	2	3	#NULL!
0	2	0	2	2	2	2	#NULL!
2	2	0	2	3	2	3	#NULL!

0	2	0	2	2	2	2	#NULL!
2	2	0	2	3	2	3	#NULL!
2	2	3	2	3	2	3	#NULL!
0	2	0	2	2	2	2	#NULL!
2	2	3	2	3	2	3	#NULL!
0	2	0	2	2	2	2	#NULL!
2	2	3	2	3	2	3	#NULL!
0	2	0	2	2	2	2	#NULL!
2	2	3	2	3	2	3	#NULL!
0	2	0	2	2	2	2	#NULL!
2	2	3	2	3	2	3	#NULL!
0	2	0	2	2	2	2	#NULL!
2	2	2	2	2	2	2	#NULL!
2	2	0	2	0	2	2	#NULL!
2	2	2	2	2	2	2	#NULL!
2	2	0	2	0	2	2	#NULL!
2	2	2	2	2	2	2	#NULL!
2	2	0	2	0	2	2	#NULL!
2	2	2	2	2	2	2	#NULL!
2	2	0	2	0	2	2	#NULL!
2	2	2	2	2	2	2	#NULL!
2	2	0	2	0	2	2	#NULL!
2	0	0	2	2	2	2	#NULL!
3	3	2	2	2	2	2	#NULL!
2	0	0	2	2	2	2	#NULL!
3	3	2	2	2	2	2	#NULL!
2	0	0	2	2	2	2	#NULL!
3	3	2	2	2	2	2	#NULL!
2	0	0	2	2	2	2	#NULL!
3	3	2	2	2	2	2	#NULL!
2	0	0	2	2	2	2	#NULL!
3	3	2	2	2	2	2	#NULL!
2	0	0	2	2	2	2	#NULL!
2	2	2	3	2	2	2	#NULL!
2	2	3	2	2	2	2	#NULL!
2	2	2	0	2	0	2	#NULL!
3	3	3	2	3	0	0	#NULL!
3	0	2	2	0	0	0	#NULL!
0	2	2	2	0	0	0	#NULL!
2	2	3	3	2	0	2	#NULL!
2	2	2	2	0	2	0	#NULL!
2	3	2	2	2	2	3	#NULL!
2	2	2	0	2	2	2	#NULL!
3	2	2	2	3	2	2	#NULL!
2	2	2	2	2	2	2	#NULL!
2	2	2	2	2	2	2	#NULL!
3	2	2	3	3	3	3	#NULL!
3	2	3	2	3	2	3	#NULL!
3	3	2	3	2	2	2	#NULL!
2	2	2	3	3	0	0	#NULL!
2	3	3	3	2	2	2	#NULL!
3	2	3	2	2	3	3	#NULL!

2	2	2	3	2	2	2	#NULL!
2	2	3	2	2	2	2	#NULL!
2	2	2	0	2	0	2	#NULL!
3	3	3	2	3	0	0	#NULL!
2	0	0	2	0	0	3	#NULL!
0	2	2	2	0	0	0	#NULL!
2	2	3	3	2	0	2	#NULL!
2	2	2	2	0	2	0	#NULL!
2	3	2	2	2	2	3	#NULL!
2	2	2	0	2	2	2	#NULL!
2	2	2	2	2	2	2	#NULL!
2	2	2	2	2	2	2	#NULL!
2	2	2	2	2	2	2	#NULL!
3	2	2	3	3	3	3	#NULL!
3	2	3	2	3	2	3	#NULL!
3	3	2	3	2	2	2	#NULL!
2	2	2	3	3	0	0	#NULL!
2	3	3	3	2	2	2	#NULL!
3	2	3	2	2	3	3	#NULL!
2	2	2	0	2	0	2	#NULL!
2	2	2	0	2	0	0	#NULL!
2	2	2	3	2	2	2	#NULL!
2	2	3	2	2	2	2	#NULL!
2	2	2	0	2	0	2	#NULL!
3	3	3	2	3	0	0	#NULL!
0	0	0	2	0	0	0	#NULL!
0	2	2	2	0	0	0	#NULL!
2	2	3	3	2	0	2	#NULL!
2	2	2	2	0	2	0	#NULL!
2	3	2	2	2	2	3	#NULL!
2	2	2	0	2	2	2	#NULL!
2	2	2	2	2	2	2	#NULL!
2	2	2	2	2	2	2	#NULL!
2	2	2	2	2	2	2	#NULL!
3	2	2	3	3	3	3	#NULL!
3	2	3	2	3	2	3	#NULL!
3	3	2	3	2	2	2	#NULL!
2	2	2	3	3	0	0	#NULL!
2	3	3	3	2	2	2	#NULL!
2	2	2	3	2	2	2	#NULL!
2	2	3	2	2	2	2	#NULL!
2	2	2	0	2	0	2	#NULL!
3	3	3	2	3	0	0	#NULL!
0	0	0	2	0	0	4	#NULL!
0	2	2	2	0	0	0	#NULL!
2	2	3	3	2	0	2	#NULL!
2	2	2	2	0	2	0	#NULL!
2	3	2	2	2	2	3	#NULL!
2	2	2	0	2	2	2	#NULL!
0	2	2	2	0	2	2	#NULL!
2	2	2	2	2	2	2	#NULL!
2	2	2	2	2	2	2	#NULL!

3	2	2	3	3	3	3	#NULL!
3	2	3	2	3	2	3	#NULL!
3	3	2	3	2	2	2	#NULL!
2	2	2	3	3	0	0	#NULL!
2	3	3	3	2	2	2	#NULL!
3	2	3	2	2	3	3	#NULL!
2	2	2	0	2	0	2	#NULL!
2	2	2	0	2	0	0	#NULL!
2	2	2	3	2	2	2	#NULL!
2	2	3	2	2	2	2	#NULL!
2	2	2	0	2	0	2	#NULL!
3	3	3	2	3	0	0	#NULL!
0	0	0	2	0	0	3	#NULL!
0	2	2	2	0	0	0	#NULL!
2	2	3	3	2	0	2	#NULL!
2	2	2	2	0	2	0	#NULL!
2	3	2	2	2	2	3	#NULL!
2	2	2	0	2	2	2	#NULL!
0	2	2	2	0	2	2	#NULL!
2	2	2	2	2	2	2	#NULL!
2	2	2	2	2	2	2	#NULL!
3	2	2	3	3	3	3	#NULL!
3	2	3	2	3	2	3	#NULL!
3	3	2	3	2	2	2	#NULL!
2	2	2	3	3	0	0	#NULL!
2	3	3	3	2	2	2	#NULL!
3	2	3	2	2	3	3	#NULL!
2	2	2	0	2	0	2	#NULL!
2	2	2	0	2	0	0	#NULL!
2	2	2	3	2	2	2	#NULL!
2	2	3	2	2	2	2	#NULL!
2	2	2	0	2	0	2	#NULL!
3	3	3	2	3	0	0	#NULL!
0	0	0	2	0	0	2	#NULL!
0	2	2	2	0	0	0	#NULL!
2	2	3	3	2	0	2	#NULL!
2	2	2	2	0	2	0	#NULL!
2	3	2	2	2	2	3	#NULL!
2	2	2	0	2	2	2	#NULL!
3	3	3	3	2	2	2	#NULL!
3	2	3	2	0	2	2	#NULL!
3	3	3	3	2	2	2	#NULL!
3	2	3	2	0	2	2	#NULL!
3	3	3	3	2	2	2	#NULL!
3	2	3	2	0	2	2	#NULL!
3	3	3	3	2	2	2	#NULL!
3	2	3	2	0	2	2	#NULL!
3	3	3	3	2	2	2	#NULL!
3	2	3	2	0	2	2	#NULL!
3	3	3	3	2	2	2	#NULL!
2	2	2	2	2	2	2	#NULL!
2	2	2	2	2	2	2	#NULL!

2	2	2	2	2	2	0	#NULL!
2	2	2	2	2	2	0	#NULL!
2	2	2	2	2	2	3	#NULL!
2	2	2	2	2	2	0	#NULL!
2	2	2	2	2	3	2	#NULL!
0	0	0	0	0	2	0	#NULL!
2	2	2	2	2	3	2	#NULL!
0	0	0	0	0	2	0	#NULL!
2	2	2	2	2	3	2	#NULL!
0	0	0	0	2	2	0	#NULL!
2	2	2	2	2	3	2	#NULL!
0	0	0	0	0	2	0	#NULL!
2	2	2	2	2	3	2	#NULL!
0	0	0	0	0	2	0	#NULL!
2	2	2	2	2	3	2	#NULL!
0	0	0	0	0	2	0	#NULL!
2	2	2	2	3	3	2	#NULL!
2	2	2	2	3	3	2	#NULL!
2	2	2	2	3	3	2	#NULL!
2	2	2	2	3	3	2	#NULL!
2	2	2	2	3	3	2	#NULL!
2	2	0	0	0	2	0	#NULL!
2	2	0	0	0	2	0	#NULL!
2	2	0	0	0	2	0	#NULL!
2	2	0	0	0	2	0	#NULL!
2	2	0	0	0	2	0	#NULL!
2	0	2	2	0	2	0	#NULL!
2	3	2	0	2	0	2	#NULL!
2	2	2	2	2	0	0	#NULL!
2	2	0	2	2	2	2	#NULL!
2	0	2	2	0	2	0	#NULL!
2	3	2	0	2	0	2	#NULL!
2	2	2	2	2	0	0	#NULL!
2	2	0	2	2	2	2	#NULL!
2	0	2	2	0	2	0	#NULL!
2	3	2	0	2	0	2	#NULL!
2	2	2	2	2	0	0	#NULL!
2	2	0	2	2	2	2	#NULL!
2	0	2	2	0	2	0	#NULL!
2	3	2	0	2	0	2	#NULL!
2	2	2	2	2	0	0	#NULL!
2	2	0	2	2	2	2	#NULL!
3	2	0	2	2	2	3	#NULL!
2	0	2	2	0	2	0	#NULL!
2	3	2	0	2	0	2	#NULL!
2	2	2	2	2	0	0	#NULL!
2	2	0	2	2	2	2	#NULL!
3	2	0	2	2	2	3	#NULL!
2	0	2	2	0	2	0	#NULL!
0	0	2	2	3	3	3	#NULL!
0	0	2	2	3	3	3	#NULL!
0	0	2	2	3	3	3	#NULL!

0	0	2	2	3	3	3	#NULL!
0	0	2	2	3	3	3	#NULL!
0	0	2	2	3	3	3	#NULL!
0	2	2	2	2	2	0	#NULL!
2	0	2	2	0	2	0	#NULL!
0	2	2	2	2	2	0	#NULL!
2	0	2	2	0	2	0	#NULL!
0	2	2	2	2	2	0	#NULL!
2	0	2	2	0	2	0	#NULL!
0	2	2	2	2	2	0	#NULL!
2	0	2	2	0	2	0	#NULL!
0	2	2	2	2	2	0	#NULL!
2	0	2	2	0	2	0	#NULL!
2	3	3	2	3	2	3	#NULL!
2	3	3	2	3	2	3	#NULL!
2	3	3	2	3	2	3	#NULL!
2	3	3	2	3	2	3	#NULL!
2	3	3	2	3	2	3	#NULL!
0	2	2	2	0	2	2	#NULL!
0	2	2	2	0	2	2	#NULL!
0	2	2	2	0	2	2	#NULL!
0	2	2	2	0	2	2	#NULL!
0	2	2	2	0	2	2	#NULL!
0	2	2	2	0	2	2	#NULL!
2	3	2	2	2	3	3	#NULL!
2	3	2	2	2	3	3	#NULL!
3	2	3	2	2	2	2	#NULL!
2	3	2	2	2	3	3	#NULL!
2	3	2	2	2	3	3	#NULL!
3	2	3	2	2	2	2	#NULL!
2	3	2	2	2	3	3	#NULL!
3	2	3	2	2	2	2	#NULL!
2	3	2	2	2	3	3	#NULL!
2	2	2	2	2	3	2	#NULL!
2	2	2	2	2	3	2	#NULL!
2	2	2	2	2	3	2	#NULL!
2	2	2	2	2	3	2	#NULL!
2	2	2	2	2	3	2	#NULL!
0	0	2	2	2	3	3	#NULL!
0	0	2	2	2	3	3	#NULL!
0	0	2	2	2	3	3	#NULL!
0	0	2	2	2	3	3	#NULL!
0	0	2	2	2	3	3	#NULL!
2	3	3	2	0	0	2	#NULL!
3	2	3	3	2	3	2	#NULL!
2	2	2	2	0	2	0	#NULL!
0	2	0	2	0	0	0	#NULL!
2	3	3	2	0	0	2	#NULL!
3	2	3	3	2	3	2	#NULL!
3	0	0	0	3	2	2	#NULL!
2	2	2	2	0	2	0	#NULL!
0	2	0	2	0	0	0	#NULL!

2	2	0	0	2	2	2	#NULL!
2	3	3	2	0	0	2	#NULL!
3	2	3	3	2	3	2	#NULL!
0	0	0	0	4	2	2	#NULL!
2	2	2	2	0	2	0	#NULL!
0	2	0	2	0	0	0	#NULL!
2	3	3	2	0	0	2	#NULL!
3	2	3	3	2	3	2	#NULL!
3	0	0	0	3	2	2	#NULL!
2	2	2	2	0	2	0	#NULL!
0	2	0	2	0	0	0	#NULL!
2	2	0	0	2	2	2	#NULL!
2	3	3	2	0	0	2	#NULL!
3	2	3	3	2	3	2	#NULL!
0	0	3	0	0	2	2	#NULL!
2	2	2	2	0	2	0	#NULL!
0	2	0	2	0	0	0	#NULL!
2	2	0	0	2	2	2	#NULL!
2	3	3	2	0	0	2	#NULL!
3	2	3	3	2	3	2	#NULL!
0	0	4	0	0	2	2	#NULL!
2	2	2	2	0	2	0	#NULL!

MSPSS1	MSPSS2	MSPSS3	MSPSS4	MSPSS5	MSPSS6	MSPSS7	MSPSS8
7	5	6	5	5	2	5	7
6	7	7	7	6	5	4	6
4	4	4	4	5	5	3	3
7	7	7	7	6	5	2	2
7	7	7	7	7	6	6	6
6	6	7	7	7	6	6	6
7	7	6	7	7	2	4	5
3	4	5	3	5	3	4	5
7	7	7	6	7	6	5	7
6	5	5	4	5	4	6	7
4	4	2	2	5	2	3	2
5	7	7	7	7	2	4	6
5	5	2	3	3	4	5	3
6	6	6	6	6	2	2	4
2	2	2	2	6	2	3	2
4	4	4	2	7	2	2	5
7	7	6	6	6	6	3	5
7	7	7	7	7	7	7	7
7	7	7	7	7	7	7	7
7	7	7	7	7	4	3	7
2	2	4	4	2	2	2	2
7	7	6	6	7	5	3	4
3	5	5	4	6	4	2	5
2	2	3	2	3	3	4	2
6	5	5	4	4	5	6	5
2	2	3	3	3	2	2	3
6	6	5	4	3	2	3	4
6	6	6	5	5	6	7	5
5	5	5	5	5	3	4	2
2	4	5	5	4	4	5	5
5	4	4	2	7	2	2	3
2	6	7	7	5	2	6	6
7	7	6	6	5	4	4	2
4	7	2	2	6	4	2	2
4	3	3	5	3	4	5	3
0	7	0	0	6	2	2	0
6	7	6	7	6	6	7	6
7	7	7	7	6	5	7	5
7	7	5	7	5	4	6	4
4	4	4	5	5	5	3	5
5	7	5	7	5	7	6	6
4	4	4	4	4	4	4	4
6	6	5	2	4	5	4	5
7	6	5	6	7	5	6	4
5	5	4	6	7	5	7	4
4	4	5	2	2	2	2	2
4	4	3	5	6	5	6	7
3	2	4	5	5	5	7	3
7	7	7	7	6	5	5	6
4	4	4	3	7	2	2	2
7	7	6	7	6	5	4	5

5	6	6	5	4	3	3	4
5	5	5	4	2	2	2	3
4	4	2	4	5	7	7	2
7	7	7	6	7	2	2	6
2	4	2	2	4	2	2	3
2	2	2	2	7	2	3	2
6	6	5	5	5	4	5	5
3	3	4	5	7	3	3	2
6	6	6	5	7	4	6	6
2	7	2	2	6	2	3	2
2	7	2	2	6	4	4	3
6	7	7	7	6	5	4	6
6	6	7	7	7	6	6	6
7	7	6	7	7	2	4	5
5	7	5	7	5	7	6	6
6	6	5	2	4	5	4	5
4	4	5	2	2	2	2	2
6	7	7	7	6	5	4	6
6	6	7	7	7	6	6	6
7	7	6	7	7	2	4	5
5	7	5	7	5	7	6	6
6	6	5	2	4	5	4	5
4	4	5	2	2	2	2	2
6	7	7	7	6	5	4	6
6	6	7	7	7	6	6	6
7	7	6	7	7	2	4	5
5	7	5	7	5	7	6	6
6	6	5	2	4	5	4	5
4	4	5	2	2	2	2	2
6	7	7	7	6	5	4	6
6	6	7	7	7	6	6	6
7	7	6	7	7	2	4	5
5	7	5	7	5	7	6	6
6	6	5	2	4	5	4	5
4	4	5	2	2	2	2	2
6	7	7	7	6	5	4	6
6	6	7	7	7	6	6	6
7	7	6	7	7	2	4	5
5	7	5	7	5	7	6	6
6	6	5	2	4	5	4	5
4	4	5	2	2	2	2	2
6	7	7	7	6	5	4	6
6	6	7	7	7	6	6	6
7	7	6	7	7	2	4	5
5	7	4	6	7	5	7	4
4	4	2	4	5	7	7	2
5	7	4	6	7	5	7	4
4	4	2	4	5	7	7	2
5	5	4	6	7	5	7	4
4	4	2	4	5	7	7	2
5	7	4	6	7	5	7	4
4	4	2	4	5	7	7	2

5	7	4	6	6	5	7	4
4	4	2	4	5	7	7	2
4	4	4	5	5	5	3	5
5	6	6	5	4	3	3	4
4	4	4	5	5	5	3	5
5	6	6	5	4	3	3	4
4	4	4	5	5	5	3	5
5	6	6	5	6	3	3	4
4	4	4	5	5	5	3	5
5	6	6	5	6	3	3	4
4	4	4	5	3	5	3	5
5	6	6	5	6	3	3	4
7	7	5	7	5	4	6	4
4	4	3	5	6	5	6	7
7	7	5	7	5	4	6	4
4	4	3	5	6	5	6	7
7	7	5	7	5	4	6	4
4	4	3	5	5	5	6	7
7	7	5	7	5	4	6	4
4	4	3	5	5	5	6	7
7	7	5	7	5	4	6	4
4	4	3	5	5	5	6	7
7	7	7	7	7	4	3	7
7	6	5	6	7	5	6	4
7	7	7	7	7	4	3	7
7	6	5	6	7	5	6	4
7	7	7	7	7	4	3	7
7	6	5	6	7	5	6	4
7	7	7	7	7	4	3	7
7	6	5	6	7	5	6	4
7	7	7	7	7	4	3	7
7	6	5	6	7	5	6	4
7	7	7	7	7	4	3	7
7	7	7	6	7	6	5	7
6	5	5	4	5	4	6	7
5	7	7	7	7	2	4	6
4	4	4	2	7	2	2	5
7	7	7	7	7	7	7	7
7	7	7	7	7	7	7	7
2	2	4	4	2	2	2	2
3	5	5	4	6	4	2	5
2	2	3	2	3	3	4	2
6	5	5	4	4	5	6	5
5	5	5	5	5	3	4	2
2	4	5	5	4	4	5	5
5	4	4	2	7	2	2	3
2	6	7	7	5	2	6	6
7	7	6	6	5	4	4	2
0	7	0	0	6	2	2	0
4	4	4	3	7	2	2	2
5	5	5	4	2	2	2	3
2	2	2	2	7	2	3	2

7	7	7	6	7	6	5	7
6	5	5	4	5	4	6	7
5	7	7	7	7	2	4	6
4	4	4	2	7	2	2	5
7	7	7	7	7	7	7	7
7	7	7	7	7	7	7	7
2	2	4	4	2	2	2	2
3	5	5	4	6	4	2	5
2	2	3	2	3	3	4	2
6	5	5	4	4	5	6	5
5	5	5	5	5	3	4	2
2	4	5	5	4	4	5	5
5	4	4	2	7	2	2	3
2	6	7	7	5	2	6	6
7	7	6	6	5	4	4	2
0	0	0	0	0	2	0	0
4	4	4	3	7	2	2	2
5	5	5	4	2	2	2	3
2	2	2	2	7	2	3	2
3	3	4	5	2	3	3	2
6	6	6	5	7	4	6	6
7	7	7	6	7	6	5	7
6	5	5	4	5	4	6	7
5	7	7	7	7	2	4	6
4	4	4	2	7	2	2	5
7	7	7	7	7	7	7	7
7	7	7	7	7	7	7	7
2	2	4	4	2	2	2	2
3	5	5	4	6	4	2	5
2	2	3	2	3	3	4	2
6	5	5	4	4	5	6	5
5	5	5	5	5	3	4	2
2	4	5	5	4	4	5	5
5	4	4	2	7	2	2	3
2	6	7	7	5	2	6	6
7	7	6	6	5	4	4	2
0	0	0	0	0	2	0	0
4	4	4	3	7	2	2	2
5	5	5	4	2	2	2	3
7	7	7	6	7	6	5	7
6	5	5	4	5	4	6	7
5	7	7	7	7	2	4	6
4	4	4	2	7	2	2	5
7	7	7	7	7	7	7	7
7	7	7	7	7	7	7	7
2	2	4	4	2	2	2	2
3	5	5	4	6	4	2	5
2	2	3	2	3	3	4	2
6	5	5	4	4	5	6	5
5	5	5	5	5	3	4	2
2	4	5	5	4	4	5	5
5	4	4	2	7	2	2	3

2	6	7	7	5	2	6	6
7	7	6	6	5	4	4	2
2	2	2	2	2	2	2	2
4	4	4	3	7	2	2	2
5	5	5	4	2	2	2	3
2	2	2	2	7	2	3	2
3	3	4	5	2	3	3	2
6	6	6	5	7	4	6	6
7	7	7	6	7	6	5	7
6	5	5	4	5	4	6	7
5	7	7	7	7	2	4	6
4	4	4	2	7	2	2	5
7	7	7	7	7	7	7	7
7	7	7	7	7	7	7	7
2	2	4	4	2	2	2	2
3	5	5	4	6	4	2	5
2	2	3	2	3	3	4	2
6	5	5	4	4	5	6	5
5	5	5	5	5	3	4	2
2	4	5	5	4	4	5	5
5	4	4	2	7	2	2	3
2	6	7	7	5	2	6	6
7	7	6	6	5	4	4	2
2	2	2	2	2	2	2	2
4	4	4	3	7	2	2	2
5	5	5	4	2	2	2	3
2	2	2	2	7	2	3	2
3	3	4	5	2	3	3	2
6	6	6	5	7	4	6	6
7	7	7	6	7	6	5	7
6	5	5	4	5	4	6	7
5	7	7	7	7	2	4	6
4	4	4	2	7	2	2	5
7	7	7	7	7	7	7	7
7	7	7	7	7	7	7	7
2	2	4	4	2	2	2	2
3	5	5	4	6	4	2	5
2	2	3	2	3	3	4	2
6	5	5	4	4	5	6	5
7	7	7	7	6	5	2	2
4	3	3	5	3	4	5	3
7	7	7	7	6	5	2	2
4	3	3	5	3	4	5	3
7	7	7	7	6	5	2	2
4	3	3	5	3	4	5	3
7	7	7	7	7	5	2	2
4	3	3	5	3	4	5	3
7	7	7	7	7	5	2	2
4	3	3	5	3	4	5	3
7	7	7	7	7	5	2	2
4	4	2	2	5	2	3	2
4	4	2	2	5	2	3	2

4	4	2	2	5	2	3	2
4	4	2	2	5	2	3	2
4	4	2	2	5	2	3	2
4	4	2	2	5	2	3	2
3	4	5	3	5	3	4	5
7	7	6	6	5	6	3	5
3	4	5	3	5	3	4	5
7	7	6	6	5	6	3	5
3	4	5	3	5	3	4	5
7	7	6	6	6	6	3	5
3	4	5	3	5	3	4	5
7	7	6	6	6	6	3	5
3	4	5	3	5	3	4	5
7	7	6	6	6	6	3	5
3	4	5	3	5	3	4	5
7	7	6	6	6	6	3	5
3	2	4	5	5	5	7	3
3	2	4	5	5	5	7	3
3	2	4	5	5	5	7	3
3	2	4	5	5	5	7	3
3	2	4	5	5	5	7	3
7	7	6	7	6	5	4	5
7	7	6	7	6	5	4	5
7	7	6	7	6	5	4	5
7	7	6	7	6	5	4	5
7	7	6	7	6	5	4	5
5	5	2	3	3	4	5	3
6	6	5	4	3	2	3	4
6	7	6	7	6	6	7	6
2	4	2	2	4	2	2	3
5	5	2	3	3	4	5	3
6	6	5	4	3	2	3	4
6	7	6	7	6	6	7	6
2	4	2	2	4	2	2	3
5	5	2	3	3	4	5	3
6	6	5	4	3	2	3	4
6	7	6	7	6	6	7	6
2	4	2	2	4	2	2	3
5	5	2	3	3	4	5	3
6	6	5	4	3	2	3	4
6	7	6	7	6	6	7	6
2	4	2	2	4	2	2	3
2	2	2	2	6	4	4	3
5	5	2	3	3	4	5	3
6	6	5	4	3	2	3	4
6	7	6	7	6	6	7	6
2	4	2	2	4	2	2	3
2	2	2	2	6	4	4	3
5	5	2	3	3	4	5	3
2	2	2	2	6	2	3	2
2	2	2	2	6	2	3	2
2	2	2	2	6	2	3	2

2	2	2	2	6	2	3	2
2	2	2	2	6	2	3	2
2	2	2	2	3	2	3	2
7	7	7	7	6	5	7	5
7	7	7	6	7	2	2	6
7	7	7	7	6	5	7	5
7	7	7	6	7	2	2	6
7	7	7	7	6	5	7	5
7	7	7	6	7	2	2	6
7	7	7	7	6	5	7	5
7	7	7	6	7	2	2	6
7	7	7	7	6	5	7	5
7	7	7	6	7	2	2	6
4	4	4	4	4	4	4	4
4	4	4	4	4	4	4	4
4	4	4	4	4	4	4	4
4	4	4	4	4	4	4	4
4	4	4	4	4	4	4	4
6	6	6	6	6	2	2	4
6	6	6	6	6	2	2	4
6	6	6	6	6	2	2	4
6	6	6	6	6	2	2	4
6	6	6	6	6	2	2	4
6	6	6	6	6	2	2	4
2	2	3	3	3	2	2	3
2	2	3	3	3	2	2	3
2	7	2	2	6	2	3	2
2	2	3	3	3	2	2	3
2	2	3	3	3	2	2	3
2	7	2	2	6	2	3	2
2	2	3	3	3	2	2	3
2	7	2	2	6	2	3	2
2	2	3	3	3	2	2	3
4	7	2	2	6	2	3	2
4	7	2	2	6	2	3	2
4	7	2	2	6	2	3	2
4	7	2	2	6	2	3	2
4	7	2	2	6	2	3	2
6	6	6	5	5	6	7	5
6	6	6	5	5	6	7	5
6	6	6	5	5	6	7	5
6	6	6	5	5	6	7	5
6	6	6	5	5	6	7	5
7	5	6	5	5	2	5	7
4	4	4	4	5	5	3	3
7	7	6	6	7	5	3	4
7	7	7	7	6	5	5	6
7	5	6	5	5	2	5	7
4	4	4	4	5	5	3	3
7	7	7	7	7	6	6	6
7	7	6	6	7	5	3	4
7	7	7	7	6	5	5	6

6	6	5	5	5	4	5	5
7	5	6	5	5	2	5	7
4	4	4	4	5	5	3	3
7	7	7	7	7	6	6	6
7	7	6	6	7	5	3	4
7	7	7	7	6	5	5	6
7	5	6	5	5	2	5	7
4	4	4	4	5	5	3	3
7	7	7	7	7	6	6	6
7	7	6	6	7	5	3	4
7	7	7	7	6	5	5	6
6	6	5	5	5	4	5	5
7	5	6	5	5	2	5	7
4	4	4	4	5	5	3	3
7	7	7	7	7	6	6	6
7	7	6	6	7	5	3	4
7	7	7	7	6	5	5	6
6	6	5	5	5	4	5	5
7	5	6	5	5	2	5	7
4	4	4	4	5	5	3	3
7	7	7	7	7	6	6	6
7	7	6	6	7	5	3	4

MSPSS9	MSPSS10	MSPSS11	MSPSS12	Namtheperson	Age2	weightbefore2
3	5	6	5	Sister	2.00	3.00
5	6	6	5	Sister	2.00	1.00
3	4	3	2	no body	2.00	3.00
7	7	5	2	Sister	2.00	1.00
6	6	6	6	Sister	3.00	3.00
6	6	6	6	Husband	2.00	1.00
6	7	2	2	Sister and Husband	2.00	1.00
3	5	3	5	Sister	1.00	2.00
7	7	6	5	Sister	1.00	1.00
5	6	5	6	Husband	1.00	1.00
3	2	4	4	Sister and Husband	2.00	3.00
3	7	2	2	Sister	1.00	1.00
4	2	3	5	Husband	2.00	2.00
2	7	6	2	Husband	2.00	2.00
4	6	2	3	Daughter	3.00	3.00
5	2	4	2	Sister	3.00	3.00
4	7	3	4	Sister, Husband	2.00	1.00
7	7	7	7	Sister, Husband	2.00	2.00
7	7	7	7	Husband	1.00	1.00
2	7	7	2	Mother, Sister, Husband	2.00	1.00
3	6	2	2	no body	3.00	3.00
5	7	5	4	Husband	2.00	2.00
5	6	5	4	Mother	2.00	1.00
2	6	2	3	Mother	3.00	3.00
6	4	6	4	Husband	1.00	1.00
4	3	4	5	no body	2.00	1.00
5	4	5	5	Sister	2.00	2.00
5	4	4	6	Husband	2.00	2.00
4	4	4	4	Sister	3.00	2.00
6	5	6	6	Husband	1.00	2.00
2	7	2	4	Sister	2.00	1.00
5	6	5	6	Sister	2.00	3.00
2	7	4	4	Husband	2.00	2.00
2	7	3	2	Sister	2.00	1.00
4	5	5	4	Mother	2.00	3.00
0	0	0	2	Sister and Husband	2.00	3.00
7	6	7	6	Husband, Mother	2.00	2.00
2	5	6	4	Husband, Mother	2.00	1.00
2	5	3	5	Husband	2.00	3.00
6	6	4	5	Friend	2.00	1.00
4	5	6	4	Husband	3.00	2.00
2	6	2	2	no body	2.00	2.00
7	5	7	5	Mother	2.00	2.00
2	2	5	7	Husband	1.00	1.00
3	6	5	7	Brother	3.00	2.00
2	2	2	4	Sister	2.00	3.00
5	4	6	6	Huaband	1.00	1.00
7	2	2	4	Friend	2.00	3.00
7	6	5	7	Husband	2.00	1.00
2	7	2	4	Sister	2.00	3.00
6	6	7	7	Sister, Husband	1.00	1.00

5	6	6	7	Daughters	2.00	1.00
2	3	3	2	Mother	2.00	2.00
6	4	2	7	Friend	2.00	2.00
7	7	5	2	Husband	2.00	1.00
2	3	2	2	Husband	2.00	3.00
3	7	2	2	Sister	3.00	3.00
4	5	6	7	Friend	2.00	3.00
2	3	4	2	Sister	2.00	2.00
6	5	6	5	Husband	2.00	2.00
4	6	2	2	Sister, Husband	2.00	3.00
4	3	2	2	Sister and Husband	2.00	3.00
5	6	6	5	Sister	2.00	1.00
6	6	6	6	Husband	2.00	1.00
6	7	2	2	Sister and Husband	2.00	1.00
4	5	6	4	Husband	3.00	2.00
7	5	7	5	Mother	2.00	2.00
2	2	2	4	Sister	2.00	3.00
5	6	6	5	Sister	2.00	1.00
6	6	6	6	Husband	2.00	1.00
6	7	2	2	Sister and Husband	2.00	1.00
4	5	6	4	Husband	3.00	2.00
7	5	7	5	Mother	2.00	2.00
2	2	2	4	Sister	2.00	3.00
5	6	6	5	Sister	2.00	1.00
6	6	6	6	Husband	2.00	1.00
6	7	2	2	Sister and Husband	2.00	1.00
4	5	6	4	Husband	3.00	2.00
7	5	7	5	Mother	2.00	2.00
2	2	2	4	Sister	2.00	3.00
5	6	6	5	Sister	2.00	1.00
6	6	6	6	Husband	2.00	1.00
6	7	2	2	Sister and Husband	2.00	1.00
4	5	6	4	Husband	3.00	2.00
7	5	7	5	Mother	2.00	2.00
2	2	2	4	Sister	2.00	3.00
5	6	6	5	Sister	2.00	1.00
6	6	6	6	Husband	2.00	1.00
6	7	2	2	Sister and Husband	2.00	1.00
4	5	6	4	Husband	3.00	2.00
7	5	7	5	Mother	2.00	2.00
2	2	2	4	Sister	2.00	3.00
5	6	6	5	Sister	2.00	1.00
6	6	6	6	Husband	2.00	1.00
6	7	2	2	Sister and Husband	2.00	1.00
3	6	5	7	Brother	3.00	2.00
6	4	2	7	Friend	2.00	2.00
3	6	5	7	Brother	3.00	2.00
6	4	2	7	Friend	2.00	2.00
3	6	5	7	Brother	3.00	2.00
6	4	2	7	Friend	2.00	2.00
3	6	5	7	Brother	3.00	2.00
6	4	2	7	Friend	2.00	2.00

3	6	5	7	Brother	3.00	2.00
6	4	2	7	Friend	2.00	2.00
6	6	4	5	Friend	2.00	1.00
5	6	6	7	Daughters	2.00	1.00
6	6	4	5	Friend	2.00	1.00
5	6	6	7	Daughters	2.00	1.00
6	6	4	5	Friend	2.00	1.00
5	6	6	7	Daughters	2.00	1.00
6	6	4	5	Friend	2.00	1.00
5	6	6	7	Daughters	2.00	1.00
6	6	4	5	Friend	2.00	1.00
5	6	6	7	Daughters	2.00	1.00
2	5	3	5	Husband	2.00	3.00
5	4	6	6	Huaband	1.00	1.00
2	5	3	5	Husband	2.00	3.00
5	4	6	6	Huaband	1.00	1.00
2	5	3	5	Husband	2.00	3.00
5	4	6	6	Huaband	1.00	1.00
2	5	3	5	Husband	2.00	3.00
5	4	6	6	Huaband	1.00	1.00
2	5	3	5	Husband	2.00	3.00
5	4	6	6	Huaband	1.00	1.00
2	7	7	2	Mother, Sister, Husband	2.00	1.00
2	2	5	7	Husband	1.00	1.00
2	7	7	2	Mother, Sister, Husband	2.00	1.00
2	2	5	7	Husband	1.00	1.00
2	7	7	2	Mother, Sister, Husband	2.00	1.00
2	2	5	7	Husband	1.00	1.00
2	7	7	2	Mother, Sister, Husband	2.00	1.00
2	2	5	7	Husband	1.00	1.00
2	7	7	2	Mother, Sister, Husband	2.00	1.00
2	2	5	7	Husband	1.00	1.00
2	7	7	2	Mother, Sister, Husband	2.00	1.00
7	7	6	5	Sister	1.00	1.00
5	6	5	6	Husband	1.00	1.00
3	7	2	2	Sister	1.00	1.00
5	2	4	2	Sister	3.00	3.00
7	7	7	7	Sister, Husband	2.00	2.00
7	7	7	7	Husband	1.00	1.00
3	6	2	2	no body	3.00	3.00
5	6	5	4	Mother	2.00	1.00
2	6	2	3	Mother	3.00	3.00
6	4	6	4	Husband	1.00	1.00
4	4	4	4	Sister	3.00	2.00
6	5	6	6	Husband	1.00	2.00
2	7	2	4	Sister	3.00	1.00
5	6	5	6	Sister	2.00	3.00
2	7	4	4	Husband	2.00	2.00
0	0	0	2	Sister and Husband	2.00	3.00
2	7	2	4	Sister	2.00	3.00
2	6	3	2	Mother	2.00	2.00
3	7	2	2	Sister	3.00	3.00

7	7	6	5	Sister	1.00	1.00
5	6	5	6	Husband	1.00	1.00
3	7	2	2	Sister	1.00	1.00
5	2	4	2	Sister	3.00	3.00
7	7	7	7	Sister, Husband	2.00	2.00
7	7	7	7	Husband	1.00	1.00
3	6	2	2	no body	3.00	3.00
5	6	5	4	Mother	2.00	1.00
2	6	2	3	Mother	3.00	3.00
6	4	6	4	Husband	1.00	1.00
4	4	4	4	Sister	3.00	2.00
6	5	6	6	Husband	1.00	2.00
2	7	2	4	Sister	3.00	1.00
5	6	5	6	Sister	2.00	3.00
2	7	4	4	Husband	2.00	2.00
0	0	0	7	Friend	2.00	3.00
2	7	2	4	Sister	2.00	3.00
2	6	3	2	Mother	2.00	2.00
3	7	2	2	Sister	3.00	3.00
2	3	4	2	Sister	2.00	2.00
6	5	6	5	Husband	2.00	2.00
7	7	6	5	Sister	1.00	1.00
5	6	5	6	Husband	1.00	1.00
3	7	2	2	Sister	1.00	1.00
5	2	4	2	Sister	3.00	3.00
7	7	7	7	Sister, Husband	2.00	2.00
7	7	7	7	Husband	1.00	1.00
3	6	2	2	no body	3.00	3.00
5	6	5	4	Mother	2.00	1.00
2	6	2	3	Mother	3.00	3.00
6	4	6	4	Husband	1.00	1.00
4	4	4	4	Sister	3.00	2.00
6	5	6	6	Husband	1.00	2.00
2	7	2	4	Sister	2.00	1.00
5	6	5	6	Sister	2.00	3.00
2	7	4	4	Husband	2.00	2.00
0	0	0	7	Friend	2.00	3.00
2	7	2	4	Sister	2.00	3.00
2	6	3	2	Mother	2.00	2.00
7	7	6	5	Sister	1.00	1.00
5	6	5	6	Husband	1.00	1.00
3	7	2	2	Sister	1.00	1.00
5	2	4	2	Sister	3.00	3.00
7	7	7	7	Sister, Husband	2.00	2.00
7	7	7	7	Husband	1.00	1.00
3	6	2	2	no body	3.00	3.00
5	6	5	4	Mother	2.00	1.00
2	6	2	3	Mother	3.00	3.00
6	4	6	4	Husband	1.00	1.00
4	4	4	4	Sister	3.00	2.00
6	5	6	6	Husband	1.00	2.00
2	7	2	4	Sister	2.00	1.00

5	6	5	6	Sister	2.00	3.00
2	7	4	4	Husband	2.00	2.00
7	2	3	7	Friend	2.00	3.00
2	7	2	4	Sister	2.00	3.00
2	6	3	2	Mother	2.00	2.00
3	7	2	2	Sister	3.00	3.00
2	3	4	2	Sister	2.00	2.00
6	5	6	5	Husband	2.00	2.00
7	7	6	5	Sister	1.00	1.00
5	6	5	6	Husband	1.00	1.00
3	7	2	2	Sister	1.00	1.00
5	2	4	2	Sister	3.00	3.00
7	7	7	7	Sister, Husband	2.00	2.00
7	7	7	7	Husband	1.00	1.00
3	6	2	2	no body	3.00	3.00
5	6	5	4	Mother	2.00	1.00
2	6	2	3	Mother	3.00	3.00
6	4	6	4	Husband	1.00	1.00
4	4	4	4	Sister	3.00	2.00
6	5	6	6	Husband	1.00	2.00
2	7	2	4	Sister	1.00	1.00
5	6	5	6	Sister	2.00	3.00
2	7	4	4	Husband	2.00	2.00
7	2	3	7	Friend	2.00	3.00
2	7	2	4	Sister	2.00	3.00
2	6	3	2	Mother	2.00	2.00
3	7	2	2	Sister	3.00	3.00
2	3	4	2	Sister	2.00	2.00
6	5	6	5	Husband	2.00	2.00
7	7	6	5	Sister	1.00	1.00
5	6	5	6	Husband	1.00	1.00
3	7	2	2	Sister	1.00	1.00
5	2	4	2	Sister	3.00	3.00
7	7	7	7	Sister, Husband	2.00	2.00
7	7	7	7	Husband	1.00	1.00
3	6	2	2	no body	3.00	3.00
5	6	5	4	Mother	2.00	1.00
2	6	2	3	Mother	3.00	3.00
6	4	6	4	Husband	1.00	1.00
7	7	5	2	Sister	2.00	1.00
4	5	5	4	Mother	2.00	3.00
7	7	5	2	Sister	2.00	1.00
4	5	5	4	Mother	2.00	3.00
7	7	5	2	Sister	2.00	1.00
4	5	5	4	Mother	2.00	3.00
7	7	5	2	Sister	2.00	1.00
4	5	5	4	Mother	2.00	3.00
7	7	5	2	Sister	2.00	1.00
4	5	5	4	Mother	2.00	3.00
7	7	5	2	Sister	2.00	1.00
3	2	4	4	Sister and Husband	2.00	3.00
3	2	4	4	Sister and Husband	2.00	3.00

3	2	4	4	Sister	2.00	3.00
3	2	4	4	Sister and Husband	2.00	3.00
3	2	4	4	Sister and Husband	2.00	3.00
3	2	4	4	Sister and Husband	2.00	3.00
3	5	3	5	Sister	1.00	2.00
4	7	3	4	Sister and Husband	2.00	1.00
3	5	3	5	Sister	1.00	2.00
4	7	3	4	Sister, Husband	2.00	1.00
3	5	3	5	Sister	1.00	2.00
4	7	3	4	Sister, Husband	2.00	1.00
3	5	3	5	Sister	1.00	2.00
4	7	3	4	Sister, Husband	2.00	1.00
3	5	3	5	Sister	1.00	2.00
4	7	3	4	Sister, Husband	2.00	1.00
3	5	3	5	Sister	1.00	2.00
4	7	3	4	Sister, Husband	2.00	1.00
7	2	2	4	Friend	2.00	3.00
7	2	2	4	Friend	2.00	3.00
7	2	2	4	Friend	2.00	3.00
7	2	2	4	Friend	2.00	3.00
7	2	2	4	Friend	2.00	3.00
6	6	7	7	Sister, Husband	1.00	1.00
6	6	7	7	Sister, Husband	1.00	1.00
6	6	7	7	Sister, Husband	1.00	1.00
6	6	7	7	Sister, Husband	1.00	1.00
6	6	7	7	Sister, Husband	1.00	1.00
4	2	3	5	Husband	2.00	2.00
5	4	5	5	Sister	2.00	2.00
7	6	7	6	Husband, Mother	2.00	2.00
2	3	2	2	Husband	2.00	3.00
4	2	3	5	Husband	2.00	2.00
5	4	5	5	Sister	2.00	2.00
7	6	7	6	Husband, Mother	2.00	2.00
2	3	2	2	Husband	2.00	3.00
4	2	3	5	Husband	2.00	2.00
5	4	5	5	Sister	2.00	2.00
7	6	7	6	Husband, Mother	2.00	2.00
2	3	2	2	Husband	2.00	3.00
4	2	3	5	Husband	2.00	2.00
5	4	5	5	Sister	2.00	2.00
7	6	7	6	Husband, Mother	2.00	2.00
2	3	2	2	Husband	2.00	3.00
4	3	2	2	Sister and Husband	2.00	3.00
4	2	3	5	Husband	2.00	2.00
5	4	5	5	Sister	2.00	2.00
7	6	7	6	Husband, Mother	2.00	2.00
2	3	2	2	Husband	2.00	3.00
4	7	2	2	Sister	2.00	3.00
4	2	3	5	Husband	2.00	2.00
4	6	2	3	Daughter	2.00	3.00
4	6	2	3	Daughter	3.00	3.00
4	6	2	3	Daughter	2.00	3.00

4	6	2	3	Daughter	1.00	3.00
4	6	2	3	Daughter	2.00	3.00
4	6	2	3	Daughter	2.00	3.00
2	5	6	4	Husband, Mother	2.00	1.00
7	7	5	2	Husband	2.00	1.00
2	5	6	4	Husband, Mother	2.00	1.00
7	7	5	2	Husband	2.00	1.00
2	5	6	4	Husband, Mother	2.00	1.00
7	7	5	2	Husband	2.00	1.00
2	5	6	4	Husband, Mother	2.00	1.00
7	7	5	2	Husband	2.00	1.00
2	5	6	4	Husband, Mother	2.00	1.00
7	7	5	2	Husband	2.00	1.00
2	6	2	2	no body	2.00	2.00
2	6	2	2	no body	2.00	2.00
2	6	2	2	no body	2.00	2.00
2	6	2	2	no body	2.00	2.00
2	6	2	2	no body	2.00	2.00
2	7	6	2	Husband	2.00	2.00
2	7	6	2	Husband	2.00	2.00
2	7	6	2	Husband	2.00	2.00
2	7	6	2	Husband	2.00	2.00
2	7	6	2	Husband	2.00	2.00
2	7	6	2	Husband	2.00	2.00
4	3	4	5	no body	2.00	1.00
4	3	4	5	no body	2.00	1.00
4	4	2	2	Sister and Husband	2.00	3.00
4	3	4	5	no body	2.00	1.00
4	3	4	5	no body	2.00	1.00
4	4	2	2	Sister and Husband	2.00	3.00
4	3	4	5	no body	2.00	1.00
4	4	2	2	Sister and Husband	2.00	3.00
4	3	4	5	no body	2.00	1.00
3	6	4	4	Sister, Husband	2.00	1.00
3	6	4	4	Sister, Husband	2.00	1.00
3	6	4	4	Sister, Husband	2.00	1.00
3	6	4	4	Sister, Husband	2.00	1.00
3	6	4	4	Sister, Husband	2.00	1.00
5	4	4	6	Husband	2.00	2.00
5	4	4	6	Husband	2.00	2.00
5	4	4	6	Husband	2.00	2.00
5	4	4	6	Husband	2.00	2.00
5	4	4	6	Husband	2.00	2.00
3	5	6	5	Sister	2.00	3.00
3	4	3	2	no body	2.00	3.00
5	7	5	4	Husband	2.00	2.00
7	6	5	7	Husband	2.00	1.00
3	5	6	5	Sister	2.00	3.00
3	4	3	2	no body	2.00	3.00
6	6	6	6	Sister	3.00	3.00
5	7	5	4	Husband	2.00	2.00
7	6	5	7	Husband	2.00	1.00

4	5	6	7	Friend	2.00	3.00
3	5	6	5	Sister	2.00	3.00
3	4	3	2	no body	2.00	3.00
6	6	6	6	Sister, Husband	3.00	3.00
5	7	5	4	Husband	2.00	2.00
7	6	5	7	Husband	2.00	1.00
3	5	6	5	Sister	2.00	3.00
3	4	3	2	no body	2.00	3.00
6	6	6	6	Sister	3.00	3.00
5	7	5	4	Husband	2.00	2.00
7	6	5	7	Husband	2.00	1.00
4	5	6	7	Friend	2.00	3.00
3	5	6	5	Sister	2.00	3.00
3	4	3	2	no body	2.00	3.00
6	6	6	6	Sister	3.00	3.00
5	7	5	4	Husband	2.00	2.00
7	6	5	7	Husband	2.00	1.00
4	5	6	7	Friend	2.00	3.00
3	5	6	5	Sister	2.00	3.00
3	4	3	2	no body	2.00	3.00
6	6	6	6	Sister	3.00	3.00
5	7	5	4	Husband	2.00	2.00

weightcurrently2	height2	fasting2	pregduration2	Age	TotNumberofChildren
3.00	2.00	2.00	1.00	28	4
2.00	2.00	1.00	1.00	30	8
3.00	2.00	3.00	1.00	35	5
2.00	2.00	1.00	2.00	32	1
3.00	2.00	1.00	1.00	42	8
1.00	2.00	1.00	2.00	30	1
2.00	2.00	1.00	1.00	32	4
3.00	3.00	2.00	1.00	25	6
2.00	2.00	1.00	1.00	22	2
2.00	2.00	1.00	1.00	23	4
3.00	2.00	1.00	2.00	29	6
2.00	2.00	1.00	2.00	22	3
3.00	2.00	2.00	2.00	32	4
3.00	3.00	1.00	1.00	38	9
3.00	2.00	3.00	2.00	42	6
3.00	2.00	3.00	2.00	42	11
1.00	2.00	1.00	1.00	29	6
2.00	2.00	2.00	2.00	27	1
2.00	2.00	1.00	1.00	19	1
2.00	2.00	1.00	1.00	40	6
3.00	2.00	3.00	2.00	43	7
1.00	2.00	1.00	2.00	32	1
2.00	2.00	2.00	1.00	29	6
3.00	2.00	3.00	2.00	42	7
1.00	2.00	1.00	2.00	23	1
3.00	2.00	1.00	1.00	26	1
3.00	2.00	3.00	1.00	33	4
2.00	2.00	1.00	2.00	32	4
3.00	2.00	2.00	1.00	42	6
3.00	2.00	1.00	2.00	25	3
2.00	2.00	1.00	1.00	34	5
3.00	2.00	3.00	2.00	40	6
3.00	2.00	3.00	2.00	36	11
2.00	2.00	1.00	1.00	32	8
3.00	2.00	3.00	2.00	36	1
3.00	2.00	3.00	1.00	35	5
3.00	2.00	2.00	2.00	34	7
2.00	2.00	1.00	2.00	31	4
3.00	2.00	1.00	2.00	27	1
2.00	2.00	1.00	2.00	30	8
2.00	2.00	2.00	2.00	42	9
2.00	2.00	3.00	1.00	34	5
2.00	2.00	2.00	2.00	29	6
1.00	2.00	1.00	2.00	18	1
3.00	2.00	2.00	1.00	42	8
3.00	2.00	3.00	2.00	39	11
1.00	2.00	1.00	1.00	24	4
3.00	2.00	3.00	2.00	29	6
2.00	2.00	1.00	1.00	38	6
3.00	2.00	3.00	2.00	32	11
2.00	3.00	1.00	2.00	24	3

1.00	2.00	1.00	1.00	28	4
3.00	2.00	3.00	2.00	38	9
3.00	2.00	3.00	2.00	30	8
2.00	2.00	1.00	1.00	29	1
3.00	2.00	3.00	2.00	34	7
3.00	2.00	3.00	1.00	42	8
3.00	2.00	2.00	1.00	40	7
3.00	2.00	2.00	1.00	38	9
2.00	2.00	1.00	1.00	38	9
3.00	2.00	3.00	2.00	28	4
3.00	2.00	3.00	1.00	29	6
2.00	2.00	1.00	1.00	30	8
1.00	2.00	1.00	2.00	30	1
2.00	2.00	1.00	1.00	32	4
2.00	2.00	2.00	2.00	42	9
2.00	2.00	2.00	2.00	29	6
3.00	2.00	3.00	2.00	39	7
2.00	2.00	1.00	1.00	30	8
1.00	2.00	1.00	2.00	30	1
2.00	2.00	1.00	1.00	32	4
2.00	2.00	2.00	2.00	42	9
2.00	2.00	2.00	2.00	29	6
3.00	2.00	3.00	2.00	39	7
2.00	2.00	1.00	1.00	30	8
1.00	2.00	1.00	2.00	30	1
2.00	2.00	1.00	1.00	32	4
2.00	2.00	2.00	2.00	42	9
2.00	2.00	2.00	2.00	29	6
3.00	2.00	3.00	2.00	39	11
2.00	2.00	1.00	1.00	30	8
1.00	2.00	1.00	2.00	30	1
2.00	2.00	1.00	1.00	32	4
2.00	2.00	2.00	2.00	42	9
2.00	2.00	2.00	2.00	29	6
3.00	2.00	3.00	2.00	39	7
2.00	2.00	1.00	1.00	30	8
1.00	2.00	1.00	2.00	30	1
2.00	2.00	1.00	1.00	32	4
2.00	2.00	2.00	2.00	42	9
2.00	2.00	2.00	2.00	29	6
3.00	2.00	3.00	2.00	39	11
2.00	2.00	1.00	1.00	30	8
1.00	2.00	1.00	2.00	30	1
2.00	2.00	1.00	1.00	32	4
3.00	2.00	2.00	1.00	42	9
3.00	2.00	3.00	2.00	30	8
3.00	2.00	2.00	1.00	42	8
3.00	2.00	3.00	2.00	30	8
3.00	2.00	2.00	1.00	42	8
3.00	2.00	3.00	2.00	30	8
3.00	2.00	2.00	1.00	42	8
3.00	2.00	3.00	2.00	30	8

3.00	2.00	2.00	1.00	42	8
3.00	2.00	3.00	2.00	30	8
2.00	2.00	1.00	2.00	30	8
1.00	2.00	1.00	1.00	28	4
2.00	2.00	1.00	2.00	30	8
1.00	2.00	1.00	1.00	28	4
2.00	2.00	1.00	2.00	30	8
1.00	2.00	1.00	1.00	28	4
2.00	2.00	1.00	2.00	30	8
1.00	2.00	1.00	1.00	28	4
2.00	2.00	1.00	2.00	30	8
1.00	2.00	1.00	1.00	28	4
3.00	2.00	1.00	2.00	27	1
1.00	2.00	1.00	1.00	24	4
3.00	2.00	1.00	2.00	27	1
1.00	2.00	1.00	1.00	24	4
3.00	2.00	1.00	2.00	27	1
1.00	2.00	1.00	1.00	24	4
3.00	2.00	1.00	2.00	27	1
1.00	2.00	1.00	1.00	24	4
3.00	2.00	1.00	2.00	27	1
1.00	2.00	1.00	1.00	24	4
2.00	2.00	1.00	1.00	40	6
1.00	2.00	1.00	2.00	18	1
2.00	2.00	1.00	1.00	40	6
1.00	2.00	1.00	2.00	18	1
2.00	2.00	1.00	1.00	40	6
1.00	2.00	1.00	2.00	18	1
2.00	2.00	1.00	1.00	40	6
1.00	2.00	1.00	2.00	18	1
2.00	2.00	1.00	1.00	40	6
1.00	2.00	1.00	2.00	18	1
2.00	2.00	1.00	1.00	40	6
2.00	2.00	1.00	1.00	22	2
2.00	2.00	1.00	1.00	23	3
2.00	2.00	1.00	2.00	22	3
3.00	2.00	3.00	2.00	42	11
2.00	2.00	2.00	2.00	27	1
2.00	2.00	1.00	1.00	19	1
3.00	2.00	3.00	2.00	43	7
2.00	2.00	2.00	1.00	29	6
3.00	2.00	3.00	2.00	42	7
1.00	2.00	1.00	2.00	23	4
3.00	2.00	2.00	1.00	42	6
3.00	2.00	1.00	2.00	25	3
2.00	2.00	1.00	1.00	42	6
3.00	2.00	3.00	2.00	40	6
3.00	2.00	3.00	2.00	36	11
3.00	2.00	3.00	1.00	35	5
3.00	2.00	3.00	2.00	32	11
3.00	2.00	3.00	2.00	38	9
3.00	2.00	3.00	1.00	42	8

2.00	2.00	1.00	1.00	22	2
2.00	2.00	1.00	1.00	23	3
2.00	2.00	1.00	2.00	22	3
3.00	2.00	3.00	2.00	42	3
2.00	2.00	2.00	2.00	27	1
2.00	2.00	1.00	1.00	19	1
3.00	2.00	3.00	2.00	43	7
2.00	2.00	2.00	1.00	29	6
3.00	2.00	3.00	2.00	42	7
1.00	2.00	1.00	2.00	23	4
3.00	2.00	2.00	1.00	42	6
3.00	2.00	1.00	2.00	25	3
2.00	2.00	1.00	1.00	42	6
3.00	2.00	3.00	2.00	40	6
3.00	2.00	3.00	2.00	36	11
3.00	2.00	3.00	1.00	35	5
3.00	2.00	3.00	2.00	32	11
3.00	2.00	3.00	2.00	38	9
3.00	2.00	3.00	1.00	42	7
3.00	2.00	2.00	1.00	38	9
2.00	2.00	1.00	1.00	38	9
2.00	2.00	1.00	1.00	22	2
2.00	2.00	1.00	1.00	23	3
2.00	2.00	1.00	2.00	22	3
3.00	2.00	3.00	2.00	42	9
2.00	2.00	2.00	2.00	27	1
2.00	2.00	1.00	1.00	19	1
3.00	2.00	3.00	2.00	43	11
2.00	2.00	2.00	1.00	29	6
3.00	2.00	3.00	2.00	42	7
1.00	2.00	1.00	2.00	23	4
3.00	2.00	2.00	1.00	42	6
3.00	2.00	1.00	2.00	25	3
2.00	2.00	1.00	1.00	39	1
3.00	2.00	3.00	2.00	40	6
3.00	2.00	3.00	2.00	36	11
3.00	2.00	3.00	1.00	35	5
3.00	2.00	3.00	2.00	32	11
3.00	2.00	3.00	2.00	38	9
2.00	2.00	1.00	1.00	22	2
2.00	2.00	1.00	1.00	23	3
2.00	2.00	1.00	2.00	22	3
3.00	2.00	3.00	2.00	42	9
2.00	2.00	2.00	2.00	27	1
2.00	2.00	1.00	1.00	19	1
3.00	2.00	3.00	2.00	43	7
2.00	2.00	2.00	1.00	29	6
3.00	2.00	3.00	2.00	42	7
1.00	2.00	1.00	2.00	23	4
3.00	2.00	2.00	1.00	42	6
3.00	2.00	1.00	2.00	25	3
2.00	2.00	1.00	1.00	40	6

3.00	2.00	3.00	2.00	40	6
3.00	2.00	3.00	2.00	36	11
3.00	2.00	3.00	1.00	35	5
3.00	2.00	3.00	2.00	32	11
3.00	2.00	3.00	2.00	38	9
3.00	2.00	3.00	1.00	42	7
3.00	2.00	2.00	1.00	38	9
2.00	2.00	1.00	1.00	38	9
2.00	2.00	1.00	1.00	22	6
2.00	2.00	1.00	1.00	23	4
2.00	2.00	1.00	2.00	22	2
3.00	2.00	3.00	2.00	42	9
2.00	2.00	2.00	2.00	27	1
2.00	2.00	1.00	1.00	19	1
3.00	2.00	3.00	2.00	43	11
2.00	2.00	2.00	1.00	29	6
3.00	2.00	3.00	2.00	42	7
1.00	2.00	1.00	2.00	23	4
3.00	2.00	2.00	1.00	42	6
3.00	2.00	1.00	2.00	25	4
2.00	2.00	1.00	1.00	23	4
3.00	2.00	3.00	2.00	40	6
3.00	2.00	3.00	2.00	36	11
3.00	2.00	3.00	1.00	35	5
3.00	2.00	3.00	2.00	32	11
3.00	2.00	3.00	2.00	38	9
3.00	2.00	3.00	1.00	42	8
3.00	2.00	2.00	1.00	38	9
2.00	2.00	1.00	1.00	38	9
2.00	2.00	1.00	1.00	22	2
2.00	2.00	1.00	1.00	23	3
2.00	2.00	1.00	2.00	22	2
3.00	2.00	3.00	2.00	42	9
2.00	2.00	2.00	2.00	27	1
2.00	2.00	1.00	1.00	19	1
3.00	2.00	3.00	2.00	43	7
2.00	2.00	2.00	1.00	29	6
3.00	2.00	3.00	2.00	42	7
1.00	2.00	1.00	2.00	23	4
2.00	2.00	1.00	2.00	32	1
3.00	2.00	3.00	2.00	36	1
2.00	2.00	1.00	2.00	32	1
3.00	2.00	3.00	2.00	36	1
2.00	2.00	1.00	2.00	32	1
3.00	2.00	3.00	2.00	36	1
2.00	2.00	1.00	2.00	32	1
3.00	2.00	3.00	2.00	36	1
2.00	2.00	1.00	2.00	32	1
3.00	2.00	3.00	2.00	36	1
2.00	2.00	1.00	2.00	32	1
3.00	2.00	1.00	2.00	29	6
3.00	2.00	1.00	2.00	29	6

3.00	2.00	1.00	2.00	29	1
3.00	2.00	1.00	2.00	29	6
3.00	2.00	1.00	2.00	29	6
3.00	2.00	1.00	2.00	29	6
3.00	3.00	2.00	1.00	25	6
1.00	2.00	1.00	1.00	29	6
3.00	3.00	2.00	1.00	25	6
1.00	2.00	1.00	1.00	29	6
3.00	3.00	2.00	1.00	25	6
1.00	2.00	1.00	1.00	29	11
3.00	3.00	2.00	1.00	25	6
1.00	2.00	1.00	1.00	29	6
3.00	3.00	2.00	1.00	25	6
1.00	2.00	1.00	1.00	29	6
3.00	3.00	2.00	1.00	25	6
1.00	2.00	1.00	1.00	29	6
3.00	2.00	3.00	2.00	29	6
3.00	2.00	3.00	2.00	29	6
3.00	2.00	3.00	2.00	29	6
3.00	2.00	3.00	2.00	29	6
3.00	2.00	3.00	2.00	29	6
2.00	3.00	1.00	2.00	24	3
2.00	3.00	1.00	2.00	24	1
2.00	3.00	1.00	2.00	24	1
2.00	3.00	1.00	2.00	24	1
2.00	3.00	1.00	2.00	24	3
3.00	2.00	2.00	2.00	32	4
3.00	2.00	3.00	1.00	33	4
3.00	2.00	2.00	2.00	34	7
3.00	2.00	3.00	2.00	34	7
3.00	2.00	2.00	2.00	32	4
3.00	2.00	3.00	1.00	33	4
3.00	2.00	2.00	2.00	34	7
3.00	2.00	3.00	2.00	34	7
3.00	2.00	2.00	2.00	32	4
3.00	2.00	3.00	1.00	33	4
3.00	2.00	2.00	2.00	34	7
3.00	2.00	3.00	2.00	34	7
3.00	2.00	2.00	2.00	32	4
3.00	2.00	3.00	1.00	33	4
3.00	2.00	2.00	2.00	34	7
3.00	2.00	3.00	2.00	34	7
3.00	2.00	3.00	1.00	29	6
3.00	2.00	2.00	2.00	32	4
3.00	2.00	3.00	1.00	33	4
3.00	2.00	2.00	2.00	34	7
3.00	2.00	3.00	2.00	34	7
3.00	2.00	3.00	1.00	29	6
3.00	2.00	2.00	2.00	32	4
3.00	2.00	3.00	2.00	36	5
3.00	2.00	3.00	2.00	42	6
3.00	2.00	3.00	2.00	30	8

3.00	2.00	3.00	2.00	23	3
3.00	2.00	3.00	2.00	36	5
3.00	2.00	3.00	2.00	37	9
2.00	2.00	1.00	2.00	31	1
2.00	2.00	1.00	1.00	29	1
2.00	2.00	1.00	2.00	31	1
2.00	2.00	1.00	1.00	29	1
2.00	2.00	1.00	2.00	31	1
2.00	2.00	1.00	1.00	29	1
2.00	2.00	1.00	2.00	31	4
2.00	2.00	1.00	1.00	29	1
2.00	2.00	1.00	2.00	31	4
2.00	2.00	1.00	1.00	29	6
2.00	2.00	3.00	1.00	34	5
2.00	2.00	3.00	1.00	34	5
2.00	2.00	3.00	1.00	34	7
2.00	2.00	3.00	1.00	34	7
2.00	2.00	3.00	1.00	34	7
3.00	3.00	1.00	1.00	38	9
3.00	3.00	1.00	1.00	38	9
3.00	3.00	1.00	1.00	38	9
3.00	3.00	1.00	1.00	38	9
3.00	3.00	1.00	1.00	38	9
3.00	3.00	1.00	1.00	38	9
3.00	2.00	1.00	1.00	26	1
3.00	2.00	1.00	1.00	26	1
3.00	2.00	3.00	2.00	28	4
3.00	2.00	1.00	1.00	26	1
3.00	2.00	1.00	1.00	26	1
3.00	2.00	3.00	2.00	28	4
3.00	2.00	1.00	1.00	26	1
3.00	2.00	3.00	2.00	28	4
3.00	2.00	1.00	1.00	26	3
2.00	2.00	1.00	1.00	32	4
2.00	2.00	1.00	1.00	32	4
2.00	2.00	1.00	1.00	32	4
2.00	2.00	1.00	1.00	32	8
2.00	2.00	1.00	1.00	32	8
2.00	2.00	1.00	2.00	32	4
2.00	2.00	1.00	2.00	32	4
2.00	2.00	1.00	2.00	32	4
2.00	2.00	1.00	2.00	32	4
2.00	2.00	1.00	2.00	32	4
3.00	2.00	2.00	1.00	28	4
3.00	2.00	3.00	1.00	35	5
1.00	2.00	1.00	2.00	32	1
2.00	2.00	1.00	1.00	38	6
3.00	2.00	2.00	1.00	28	4
3.00	2.00	3.00	1.00	35	5
3.00	2.00	1.00	1.00	42	8
1.00	2.00	1.00	2.00	32	1
2.00	2.00	1.00	1.00	38	6

3.00	2.00	2.00	1.00	40	7
3.00	2.00	2.00	1.00	28	4
3.00	2.00	3.00	1.00	35	5
3.00	2.00	1.00	1.00	42	8
1.00	2.00	1.00	2.00	32	1
2.00	2.00	1.00	1.00	38	6
3.00	2.00	2.00	1.00	28	4
3.00	2.00	3.00	1.00	35	5
3.00	2.00	1.00	1.00	42	8
1.00	2.00	1.00	2.00	32	1
2.00	2.00	1.00	1.00	38	6
3.00	2.00	2.00	1.00	40	7
3.00	2.00	2.00	1.00	28	4
3.00	2.00	3.00	1.00	35	5
3.00	2.00	1.00	1.00	42	8
1.00	2.00	1.00	2.00	32	1
2.00	2.00	1.00	1.00	38	6
3.00	2.00	2.00	1.00	40	7
3.00	2.00	2.00	1.00	28	4
3.00	2.00	3.00	1.00	35	5
3.00	2.00	1.00	1.00	42	8
1.00	2.00	1.00	2.00	32	1

HBA1C2	PregDuration	weightdifference	weigytgain	Weightfinalscale	Height
2.00	25	8.00	1.00	61.00	150
1.00	25	11.00	2.00	83.00	160
3.00	25	10.00	2.00	65.00	155
2.00	26	11.00	2.00	83.00	154
2.00	24	10.00	2.00	67.00	155
1.00	26	12.00	2.00	112.00	162
2.00	24	12.00	2.00	92.00	170
2.00	25	10.00	2.00	70.00	155
2.00	24	12.00	2.00	82.00	160
2.00	25	11.00	2.00	83.00	160
2.00	26	11.00	2.00	69.00	155
1.00	28	12.00	2.00	92.00	170
2.00	28	11.00	2.00	71.00	155
2.00	24	11.00	2.00	78.00	165
3.00	26	8.00	1.00	61.00	150
3.00	28	6.00	1.00	48.00	148
2.00	25	12.00	2.00	112.00	162
2.00	26	11.00	2.00	78.00	162
2.00	24	11.00	2.00	83.00	154
2.00	25	11.00	2.00	83.00	154
3.00	28	7.00	1.00	53.00	148
2.00	28	12.00	2.00	122.00	165
2.00	24	11.00	2.00	83.00	154
3.00	28	6.00	1.00	47.00	150
2.00	28	12.00	2.00	102.00	160
2.00	24	11.00	2.00	73.00	165
2.00	24	10.00	2.00	67.00	155
2.00	27	11.00	1.00	78.00	162
2.00	25	11.00	2.00	71.00	155
1.00	26	11.00	2.00	78.00	162
1.00	24	11.00	2.00	83.00	154
3.00	26	8.00	1.00	56.00	148
2.00	26	11.00	2.00	69.00	155
2.00	24	11.00	2.00	83.00	160
2.00	28	8.00	1.00	62.00	155
3.00	24	7.00	1.00	47.00	155
2.00	26	11.00	2.00	71.00	155
1.00	26	11.00	2.00	83.00	165
2.00	27	8.00	1.00	58.00	150
2.00	28	12.00	2.00	92.00	170
2.00	26	11.00	2.00	78.00	160
3.00	25	11.00	2.00	78.00	162
2.00	26	11.00	2.00	78.00	162
1.00	26	12.00	2.00	102.00	160
2.00	24	11.00	2.00	71.00	155
3.00	26	8.00	1.00	61.00	150
1.00	24	12.00	2.00	112.00	162
2.00	27	8.00	1.00	56.00	150
1.00	24	11.00	2.00	83.00	154
3.00	26	8.00	1.00	61.00	150
2.00	26	12.00	2.00	82.00	160

1.00	24	12.00	2.00	102.00	160
3.00	26	10.00	2.00	67.00	155
3.00	26	11.00	2.00	69.00	155
2.00	24	12.00	2.00	92.00	160
2.00	27	8.00	1.00	61.00	150
3.00	25	8.00	1.00	56.00	148
2.00	25	8.00	1.00	64.00	155
3.00	24	11.00	2.00	69.00	155
2.00	24	11.00	2.00	83.00	160
3.00	26	10.00	2.00	65.00	155
3.00	24	8.00	1.00	58.00	150
1.00	25	11.00	2.00	83.00	160
1.00	26	12.00	2.00	112.00	162
2.00	24	12.00	2.00	92.00	160
2.00	26	11.00	2.00	78.00	160
2.00	26	11.00	2.00	78.00	162
3.00	26	8.00	1.00	61.00	150
1.00	25	11.00	2.00	83.00	160
1.00	26	12.00	2.00	112.00	162
2.00	24	12.00	2.00	92.00	160
2.00	26	11.00	2.00	78.00	162
2.00	26	11.00	2.00	78.00	162
3.00	26	8.00	1.00	61.00	150
1.00	25	11.00	2.00	83.00	160
1.00	26	12.00	2.00	102.00	160
2.00	24	12.00	2.00	92.00	160
2.00	26	11.00	2.00	78.00	160
2.00	26	11.00	2.00	78.00	162
3.00	26	8.00	1.00	61.00	150
1.00	25	11.00	2.00	83.00	160
1.00	26	12.00	2.00	112.00	162
2.00	24	12.00	2.00	92.00	160
2.00	26	11.00	2.00	78.00	160
2.00	26	11.00	2.00	78.00	162
3.00	26	8.00	1.00	61.00	150
1.00	25	11.00	2.00	83.00	160
1.00	26	12.00	2.00	102.00	160
2.00	24	12.00	2.00	92.00	160
2.00	26	11.00	2.00	78.00	160
2.00	26	11.00	2.00	78.00	162
3.00	26	8.00	1.00	61.00	150
1.00	25	11.00	2.00	83.00	160
1.00	26	12.00	2.00	102.00	160
2.00	24	12.00	2.00	92.00	160
2.00	24	11.00	2.00	71.00	155
3.00	26	11.00	2.00	69.00	155
2.00	24	11.00	2.00	71.00	155
3.00	26	11.00	2.00	69.00	155
2.00	24	11.00	2.00	69.00	155
3.00	26	10.00	2.00	70.00	155
2.00	24	11.00	2.00	71.00	155
3.00	26	11.00	2.00	69.00	155

2.00	24	11.00	2.00	71.00	155
3.00	26	11.00	2.00	69.00	155
2.00	28	12.00	2.00	92.00	170
1.00	24	12.00	2.00	102.00	160
2.00	28	12.00	2.00	92.00	170
1.00	24	12.00	2.00	102.00	160
2.00	28	12.00	2.00	92.00	170
1.00	24	12.00	2.00	102.00	160
2.00	28	12.00	2.00	92.00	170
1.00	24	12.00	2.00	102.00	160
2.00	28	12.00	2.00	92.00	170
1.00	24	12.00	2.00	102.00	160
2.00	27	8.00	1.00	58.00	150
1.00	24	12.00	2.00	112.00	162
2.00	27	8.00	1.00	58.00	150
1.00	24	12.00	2.00	112.00	162
2.00	27	8.00	1.00	58.00	150
1.00	24	12.00	2.00	112.00	162
2.00	27	8.00	1.00	58.00	150
1.00	24	12.00	2.00	112.00	162
2.00	27	8.00	1.00	58.00	150
1.00	24	12.00	2.00	112.00	162
2.00	25	11.00	2.00	83.00	154
1.00	26	12.00	2.00	102.00	160
2.00	25	11.00	2.00	83.00	154
1.00	26	12.00	2.00	102.00	160
2.00	25	11.00	2.00	83.00	154
1.00	26	12.00	2.00	102.00	160
2.00	25	11.00	2.00	83.00	154
1.00	26	12.00	2.00	102.00	160
2.00	25	11.00	2.00	83.00	154
1.00	26	12.00	2.00	102.00	160
2.00	25	11.00	2.00	83.00	154
2.00	24	12.00	2.00	82.00	160
2.00	25	11.00	2.00	83.00	160
1.00	28	12.00	2.00	92.00	170
3.00	28	6.00	1.00	48.00	148
2.00	26	11.00	2.00	78.00	162
2.00	24	11.00	2.00	83.00	154
3.00	28	7.00	1.00	53.00	148
2.00	24	11.00	2.00	83.00	154
3.00	28	6.00	1.00	47.00	150
2.00	28	12.00	2.00	102.00	160
2.00	25	11.00	2.00	72.00	165
1.00	26	11.00	2.00	78.00	165
1.00	24	11.00	2.00	83.00	154
3.00	26	8.00	1.00	56.00	148
2.00	26	11.00	2.00	69.00	155
3.00	24	7.00	1.00	47.00	155
3.00	26	8.00	1.00	61.00	150
3.00	26	10.00	2.00	65.00	155
3.00	25	8.00	1.00	56.00	148

2.00	24	12.00	2.00	82.00	160
2.00	25	11.00	2.00	83.00	160
1.00	28	12.00	2.00	92.00	170
3.00	28	6.00	1.00	48.00	148
2.00	26	11.00	2.00	78.00	162
2.00	24	11.00	2.00	83.00	154
3.00	28	7.00	1.00	53.00	148
2.00	24	11.00	2.00	83.00	154
3.00	28	6.00	1.00	47.00	150
2.00	28	12.00	2.00	102.00	160
2.00	25	11.00	2.00	71.00	155
1.00	26	11.00	2.00	78.00	165
1.00	24	11.00	2.00	83.00	154
3.00	26	8.00	1.00	56.00	148
2.00	26	11.00	2.00	69.00	155
3.00	24	7.00	1.00	47.00	155
3.00	26	8.00	1.00	61.00	150
3.00	26	10.00	2.00	65.00	155
3.00	25	8.00	1.00	56.00	148
3.00	24	11.00	2.00	69.00	155
2.00	24	11.00	2.00	83.00	160
2.00	24	12.00	2.00	82.00	160
2.00	25	11.00	2.00	83.00	160
1.00	28	12.00	2.00	92.00	170
3.00	28	6.00	1.00	48.00	148
2.00	26	11.00	2.00	78.00	162
2.00	24	11.00	2.00	83.00	154
3.00	28	7.00	1.00	53.00	148
2.00	24	11.00	2.00	83.00	154
3.00	28	6.00	1.00	47.00	150
2.00	28	12.00	2.00	102.00	160
2.00	25	11.00	2.00	72.00	165
1.00	26	11.00	2.00	78.00	162
1.00	24	11.00	2.00	83.00	154
3.00	26	8.00	1.00	56.00	150
2.00	26	11.00	2.00	69.00	155
3.00	24	7.00	1.00	47.00	155
3.00	26	8.00	1.00	61.00	150
3.00	26	10.00	2.00	65.00	155
2.00	24	12.00	2.00	82.00	160
2.00	25	11.00	2.00	83.00	160
1.00	28	12.00	2.00	92.00	170
3.00	28	6.00	1.00	48.00	148
2.00	26	11.00	2.00	78.00	162
2.00	24	11.00	2.00	83.00	154
3.00	28	7.00	1.00	53.00	148
2.00	24	11.00	2.00	83.00	154
3.00	28	6.00	1.00	47.00	150
2.00	28	12.00	2.00	102.00	160
2.00	25	11.00	2.00	72.00	165
1.00	26	11.00	2.00	78.00	162
1.00	24	11.00	2.00	83.00	154

3.00	26	8.00	1.00	56.00	148
2.00	26	11.00	2.00	69.00	155
3.00	24	7.00	1.00	47.00	155
3.00	26	8.00	1.00	61.00	150
3.00	26	10.00	2.00	65.00	155
3.00	25	8.00	1.00	56.00	148
3.00	24	11.00	2.00	69.00	155
2.00	24	11.00	2.00	83.00	160
2.00	24	12.00	2.00	82.00	160
2.00	25	11.00	2.00	83.00	160
1.00	28	12.00	2.00	92.00	170
3.00	28	6.00	1.00	48.00	148
2.00	26	11.00	2.00	78.00	162
2.00	24	11.00	2.00	83.00	154
3.00	28	6.00	1.00	49.00	148
2.00	24	11.00	2.00	83.00	154
3.00	28	6.00	1.00	47.00	150
2.00	28	12.00	2.00	92.00	170
2.00	25	11.00	2.00	72.00	165
1.00	26	11.00	2.00	78.00	162
1.00	24	11.00	2.00	83.00	154
3.00	26	8.00	1.00	56.00	148
2.00	26	11.00	2.00	69.00	155
3.00	24	7.00	1.00	47.00	155
3.00	26	8.00	1.00	61.00	150
3.00	26	10.00	2.00	65.00	155
3.00	25	7.00	1.00	57.00	150
3.00	24	11.00	2.00	69.00	155
2.00	24	11.00	2.00	83.00	160
2.00	24	12.00	2.00	82.00	160
2.00	25	11.00	2.00	78.00	160
1.00	28	12.00	2.00	92.00	170
3.00	28	6.00	1.00	48.00	148
2.00	26	11.00	2.00	78.00	162
2.00	24	11.00	2.00	83.00	154
3.00	28	7.00	1.00	53.00	148
2.00	24	11.00	2.00	83.00	165
3.00	28	6.00	1.00	47.00	150
2.00	28	12.00	2.00	92.00	170
2.00	26	11.00	2.00	83.00	154
2.00	28	8.00	1.00	62.00	155
2.00	26	11.00	2.00	83.00	154
2.00	28	8.00	1.00	62.00	155
2.00	26	11.00	2.00	83.00	154
2.00	28	8.00	1.00	62.00	155
2.00	26	11.00	2.00	83.00	154
2.00	28	8.00	1.00	62.00	155
2.00	26	11.00	2.00	83.00	154
2.00	28	8.00	1.00	62.00	155
2.00	26	11.00	2.00	83.00	165
2.00	26	11.00	2.00	69.00	155
2.00	26	11.00	2.00	69.00	155

2.00	26	10.00	2.00	67.00	155
2.00	26	11.00	2.00	69.00	155
2.00	26	11.00	2.00	69.00	155
2.00	26	11.00	2.00	69.00	155
2.00	25	10.00	2.00	70.00	155
2.00	25	12.00	2.00	112.00	162
2.00	25	10.00	2.00	70.00	155
2.00	25	12.00	2.00	112.00	162
2.00	25	10.00	2.00	70.00	155
2.00	25	12.00	2.00	112.00	162
2.00	25	10.00	2.00	70.00	155
2.00	25	12.00	2.00	112.00	162
2.00	25	10.00	2.00	70.00	155
2.00	25	12.00	2.00	112.00	162
2.00	25	10.00	2.00	70.00	155
2.00	25	12.00	2.00	112.00	162
2.00	27	8.00	1.00	56.00	150
2.00	27	8.00	1.00	56.00	150
2.00	27	8.00	1.00	56.00	150
2.00	27	8.00	1.00	56.00	150
2.00	27	8.00	1.00	56.00	150
2.00	26	12.00	2.00	82.00	160
2.00	26	12.00	2.00	82.00	160
2.00	26	12.00	2.00	82.00	160
2.00	26	12.00	2.00	82.00	160
2.00	26	11.00	2.00	83.00	154
2.00	28	11.00	2.00	69.00	155
2.00	24	10.00	2.00	67.00	155
2.00	26	11.00	2.00	71.00	155
2.00	27	8.00	1.00	61.00	150
2.00	28	11.00	2.00	69.00	155
2.00	24	10.00	2.00	67.00	155
2.00	26	11.00	2.00	71.00	155
2.00	27	8.00	1.00	61.00	150
2.00	28	11.00	2.00	69.00	155
2.00	24	10.00	2.00	67.00	155
2.00	26	11.00	2.00	71.00	155
2.00	27	8.00	1.00	61.00	150
2.00	28	11.00	2.00	69.00	155
2.00	24	10.00	2.00	67.00	155
2.00	26	11.00	2.00	71.00	155
2.00	27	8.00	1.00	61.00	150
3.00	24	8.00	1.00	56.00	150
2.00	28	11.00	2.00	69.00	155
2.00	24	10.00	2.00	67.00	155
2.00	26	11.00	2.00	71.00	155
2.00	27	8.00	1.00	61.00	150
3.00	24	8.00	1.00	56.00	150
2.00	28	11.00	2.00	69.00	155
3.00	26	8.00	1.00	61.00	150
3.00	26	8.00	1.00	61.00	150
3.00	26	8.00	1.00	61.00	150

3.00	26	8.00	1.00	61.00	150
3.00	26	8.00	1.00	61.00	150
3.00	26	8.00	1.00	61.00	150
1.00	26	11.00	2.00	83.00	165
2.00	24	12.00	2.00	92.00	160
1.00	26	11.00	2.00	83.00	165
2.00	24	12.00	2.00	92.00	160
1.00	26	11.00	2.00	83.00	165
2.00	24	12.00	2.00	92.00	160
1.00	26	11.00	2.00	83.00	165
2.00	24	12.00	2.00	92.00	160
1.00	26	11.00	2.00	83.00	165
2.00	24	12.00	2.00	82.00	160
3.00	25	11.00	2.00	78.00	162
3.00	25	11.00	2.00	78.00	162
3.00	25	11.00	2.00	78.00	162
3.00	25	11.00	2.00	78.00	162
3.00	25	11.00	2.00	78.00	162
2.00	24	11.00	2.00	75.00	165
2.00	24	11.00	2.00	75.00	165
2.00	24	11.00	2.00	75.00	165
2.00	24	11.00	2.00	75.00	165
2.00	24	11.00	2.00	75.00	165
2.00	24	11.00	2.00	75.00	165
2.00	24	11.00	2.00	73.00	165
2.00	24	11.00	2.00	73.00	165
3.00	26	10.00	2.00	65.00	155
2.00	24	11.00	2.00	73.00	165
2.00	24	11.00	2.00	73.00	165
3.00	26	8.00	1.00	64.00	155
2.00	24	11.00	2.00	73.00	165
3.00	26	8.00	1.00	64.00	155
2.00	24	11.00	2.00	71.00	150
2.00	24	11.00	2.00	83.00	165
2.00	24	11.00	2.00	83.00	165
2.00	24	11.00	2.00	83.00	165
2.00	24	11.00	2.00	83.00	160
2.00	24	11.00	2.00	83.00	160
2.00	27	11.00	1.00	78.00	162
2.00	27	11.00	1.00	78.00	162
2.00	27	11.00	1.00	78.00	162
2.00	27	11.00	1.00	78.00	162
2.00	27	11.00	1.00	78.00	162
2.00	25	8.00	1.00	60.00	150
3.00	25	10.00	2.00	65.00	155
2.00	28	12.00	2.00	122.00	165
1.00	24	11.00	2.00	83.00	154
2.00	25	8.00	1.00	60.00	150
3.00	25	10.00	2.00	65.00	155
2.00	24	10.00	2.00	67.00	155
2.00	28	12.00	2.00	122.00	165
1.00	24	11.00	2.00	83.00	154

2.00	25	8.00	1.00	64.00	155
2.00	25	8.00	1.00	60.00	150
3.00	25	10.00	2.00	65.00	155
2.00	24	10.00	2.00	67.00	155
2.00	28	12.00	2.00	112.00	162
1.00	24	11.00	2.00	83.00	154
2.00	25	8.00	1.00	60.00	150
3.00	25	10.00	2.00	65.00	155
2.00	24	10.00	2.00	67.00	155
2.00	28	12.00	2.00	112.00	162
1.00	24	11.00	2.00	83.00	154
2.00	25	8.00	1.00	64.00	155
2.00	25	8.00	1.00	60.00	150
3.00	25	10.00	2.00	65.00	155
2.00	24	10.00	2.00	67.00	155
2.00	28	12.00	2.00	112.00	162
1.00	24	11.00	2.00	83.00	154
2.00	25	8.00	1.00	64.00	155
2.00	25	8.00	1.00	60.00	150
3.00	25	10.00	2.00	65.00	155
2.00	24	10.00	2.00	67.00	155
2.00	28	12.00	2.00	112.00	162

WeightBefoscale	stress1_5	depress1_5	anxiety1_5	bmi
53	3.00	5.00	5.00	27.11
72	1.00	2.00	2.00	32.42
55	4.00	2.00	2.00	27.06
72	3.00	3.00	3.00	35.00
57	2.00	2.00	2.00	27.89
100	3.00	2.00	2.00	42.68
80	3.00	2.00	2.00	31.83
60	3.00	3.00	3.00	29.14
70	3.00	3.00	3.00	32.03
72	2.00	3.00	3.00	32.42
58	5.00	3.00	3.00	28.72
80	4.00	2.00	2.00	31.83
60	3.00	3.00	3.00	29.55
67	4.00	3.00	3.00	28.65
53	4.00	3.00	3.00	27.11
42	4.00	3.00	3.00	21.91
100	4.00	3.00	3.00	42.68
67	2.00	1.00	1.00	29.72
72	4.00	3.00	3.00	35.00
72	4.00	3.00	3.00	35.00
46	3.00	3.00	3.00	24.20
110	4.00	3.00	3.00	44.81
72	4.00	3.00	3.00	35.00
41	3.00	3.00	4.00	20.89
90	5.00	3.00	3.00	39.84
62	3.00	3.00	3.00	26.81
57	2.00	3.00	3.00	27.89
67	3.00	5.00	5.00	29.72
60	4.00	3.00	3.00	29.55
67	4.00	3.00	3.00	29.72
72	4.00	3.00	3.00	35.00
48	1.00	3.00	3.00	25.57
58	3.00	3.00	3.00	28.72
72	2.00	3.00	3.00	32.42
54	4.00	3.00	3.00	25.81
40	3.00	3.00	3.00	19.56
60	3.00	3.00	3.00	29.55
72	4.00	3.00	3.00	30.49
50	4.00	3.00	3.00	25.78
80	3.00	5.00	5.00	31.83
67	4.00	3.00	3.00	30.47
67	4.00	2.00	2.00	29.72
67	2.00	3.00	3.00	29.72
90	3.00	3.00	3.00	39.84
60	3.00	3.00	3.00	29.55
53	3.00	4.00	4.00	27.11
100	3.00	3.00	3.00	42.68
48	3.00	5.00	5.00	24.89
72	4.00	3.00	3.00	35.00
53	3.00	3.00	3.00	27.11
70	3.00	1.00	1.00	32.03

90	4.00	2.00	2.00	39.84
57	3.00	3.00	3.00	27.89
58	3.00	3.00	3.00	28.72
80	3.00	3.00	3.00	35.94
53	3.00	3.00	3.00	27.11
48	3.00	3.00	3.00	25.57
56	4.00	3.00	3.00	26.64
58	3.00	3.00	3.00	28.72
72	3.00	3.00	3.00	32.42
55	3.00	3.00	5.00	27.06
50	3.00	3.00	3.00	25.78
72	1.00	2.00	2.00	32.42
100	3.00	2.00	2.00	42.68
80	3.00	1.00	1.00	35.94
67	4.00	3.00	3.00	30.47
67	2.00	3.00	3.00	29.72
53	3.00	5.00	5.00	27.11
72	1.00	2.00	2.00	32.42
100	3.00	2.00	2.00	42.68
80	4.00	1.00	1.00	35.94
67	4.00	3.00	3.00	29.72
67	2.00	3.00	3.00	29.72
53	3.00	5.00	5.00	27.11
72	1.00	2.00	2.00	32.42
90	3.00	2.00	2.00	39.84
80	3.00	2.00	1.00	35.94
67	4.00	4.00	4.00	30.47
67	2.00	3.00	3.00	29.72
53	3.00	3.00	3.00	27.11
72	1.00	2.00	2.00	32.42
100	3.00	2.00	2.00	42.68
80	3.00	1.00	1.00	35.94
67	4.00	3.00	3.00	30.47
67	2.00	3.00	3.00	29.72
53	3.00	5.00	5.00	27.11
72	1.00	2.00	2.00	32.42
90	3.00	2.00	2.00	39.84
80	4.00	1.00	1.00	35.94
67	4.00	3.00	3.00	30.47
67	2.00	3.00	3.00	29.72
53	3.00	3.00	4.00	27.11
72	1.00	2.00	2.00	32.42
90	3.00	2.00	2.00	39.84
80	3.00	1.00	1.00	35.94
60	3.00	3.00	3.00	29.55
58	3.00	3.00	3.00	28.72
60	2.00	3.00	3.00	29.55
58	3.00	3.00	3.00	28.72
58	3.00	3.00	3.00	28.72
60	3.00	3.00	3.00	29.14
60	2.00	3.00	3.00	29.55
58	3.00	3.00	3.00	28.72

60	2.00	3.00	3.00	29.55
58	3.00	3.00	3.00	28.72
80	3.00	3.00	5.00	31.83
90	4.00	2.00	2.00	39.84
80	3.00	3.00	3.00	31.83
90	4.00	2.00	2.00	39.84
80	2.00	3.00	3.00	31.83
90	4.00	2.00	2.00	39.84
80	2.00	3.00	3.00	31.83
90	4.00	2.00	2.00	39.84
80	2.00	3.00	3.00	31.83
90	4.00	2.00	2.00	39.84
50	4.00	3.00	3.00	25.78
100	3.00	3.00	3.00	42.68
50	4.00	3.00	3.00	25.78
100	3.00	3.00	3.00	42.68
50	4.00	3.00	3.00	25.78
100	4.00	3.00	3.00	42.68
50	3.00	3.00	3.00	25.78
100	3.00	3.00	3.00	42.68
50	3.00	3.00	3.00	25.78
100	3.00	3.00	3.00	42.68
72	5.00	3.00	3.00	35.00
90	3.00	3.00	3.00	39.84
72	5.00	3.00	3.00	35.00
90	3.00	3.00	3.00	39.84
72	5.00	3.00	3.00	35.00
90	4.00	3.00	3.00	39.84
72	5.00	3.00	3.00	35.00
90	4.00	3.00	3.00	39.84
72	5.00	3.00	3.00	35.00
90	4.00	3.00	3.00	39.84
72	4.00	3.00	3.00	35.00
70	3.00	3.00	3.00	32.03
72	2.00	3.00	3.00	32.42
80	4.00	2.00	2.00	31.83
42	4.00	3.00	3.00	21.91
67	2.00	1.00	1.00	29.72
72	4.00	3.00	3.00	35.00
46	4.00	3.00	3.00	24.20
72	4.00	3.00	3.00	35.00
41	3.00	3.00	4.00	20.89
90	5.00	3.00	3.00	39.84
61	3.00	3.00	3.00	26.45
67	4.00	3.00	3.00	28.65
72	4.00	3.00	3.00	35.00
48	2.00	3.00	3.00	25.57
58	3.00	3.00	3.00	28.72
40	3.00	3.00	3.00	19.56
53	3.00	3.00	3.00	27.11
55	3.00	3.00	3.00	27.06
48	3.00	3.00	3.00	25.57

70	3.00	3.00	3.00	32.03
72	2.00	3.00	3.00	32.42
80	4.00	2.00	2.00	31.83
42	4.00	3.00	3.00	21.91
67	2.00	1.00	1.00	29.72
72	4.00	3.00	3.00	35.00
46	3.00	2.00	2.00	24.20
72	4.00	3.00	3.00	35.00
41	3.00	5.00	5.00	20.89
90	5.00	3.00	3.00	39.84
60	4.00	3.00	3.00	29.55
67	4.00	3.00	3.00	28.65
72	4.00	3.00	3.00	35.00
48	2.00	3.00	3.00	25.57
58	3.00	3.00	3.00	28.72
40	3.00	3.00	3.00	19.56
53	3.00	3.00	3.00	27.11
55	3.00	3.00	3.00	27.06
48	3.00	3.00	3.00	25.57
58	3.00	3.00	3.00	28.72
72	3.00	3.00	3.00	32.42
70	3.00	3.00	3.00	32.03
72	2.00	3.00	3.00	32.42
80	4.00	2.00	2.00	31.83
42	4.00	3.00	3.00	21.91
67	2.00	1.00	1.00	29.72
72	3.00	3.00	3.00	35.00
46	4.00	3.00	3.00	24.20
72	4.00	3.00	3.00	35.00
41	3.00	3.00	4.00	20.89
90	5.00	3.00	3.00	39.84
61	3.00	3.00	3.00	26.45
67	4.00	3.00	3.00	29.72
72	4.00	3.00	3.00	35.00
48	3.00	3.00	3.00	24.89
58	3.00	3.00	3.00	28.72
40	3.00	3.00	3.00	19.56
53	3.00	3.00	3.00	27.11
55	3.00	3.00	3.00	27.06
70	3.00	5.00	5.00	32.03
72	2.00	3.00	3.00	32.42
80	4.00	2.00	2.00	31.83
42	4.00	3.00	3.00	21.91
67	3.00	1.00	1.00	29.72
72	3.00	3.00	3.00	35.00
46	3.00	2.00	2.00	24.20
72	4.00	3.00	3.00	35.00
41	3.00	3.00	4.00	20.89
90	5.00	3.00	3.00	39.84
61	4.00	4.00	3.00	26.45
67	4.00	3.00	3.00	29.72
72	4.00	3.00	3.00	35.00

48	3.00	5.00	5.00	25.57
58	4.00	3.00	3.00	28.72
40	4.00	3.00	3.00	19.56
53	4.00	3.00	3.00	27.11
55	3.00	3.00	3.00	27.06
48	3.00	3.00	3.00	25.57
58	3.00	3.00	3.00	28.72
72	3.00	3.00	3.00	32.42
70	3.00	3.00	3.00	32.03
72	3.00	3.00	3.00	32.42
80	4.00	2.00	2.00	31.83
42	4.00	3.00	3.00	21.91
67	3.00	1.00	1.00	29.72
72	4.00	3.00	3.00	35.00
43	3.00	3.00	3.00	22.37
72	4.00	3.00	3.00	35.00
41	3.00	3.00	4.00	20.89
80	5.00	3.00	3.00	31.83
61	3.00	3.00	3.00	26.45
67	4.00	3.00	3.00	29.72
72	4.00	3.00	3.00	35.00
48	3.00	3.00	5.00	25.57
58	4.00	3.00	3.00	28.72
40	4.00	3.00	3.00	19.56
53	4.00	3.00	3.00	27.11
55	3.00	3.00	3.00	27.06
50	3.00	3.00	3.00	25.33
58	3.00	3.00	3.00	28.72
72	3.00	3.00	3.00	32.42
70	3.00	3.00	3.00	32.03
67	3.00	3.00	3.00	30.47
80	4.00	2.00	2.00	31.83
42	3.00	3.00	3.00	21.91
67	3.00	1.00	1.00	29.72
72	4.00	3.00	3.00	35.00
46	4.00	3.00	3.00	24.20
72	4.00	3.00	3.00	30.49
41	3.00	3.00	4.00	20.89
80	5.00	3.00	3.00	31.83
72	3.00	3.00	3.00	35.00
54	4.00	3.00	3.00	25.81
72	3.00	3.00	3.00	35.00
54	4.00	3.00	3.00	25.81
72	3.00	3.00	3.00	35.00
54	4.00	3.00	3.00	25.81
72	3.00	3.00	3.00	35.00
54	4.00	5.00	5.00	25.81
72	3.00	3.00	3.00	35.00
54	4.00	3.00	3.00	25.81
72	3.00	3.00	3.00	30.49
58	4.00	3.00	3.00	28.72
58	5.00	3.00	3.00	28.72

57	4.00	3.00	3.00	27.89
58	5.00	3.00	3.00	28.72
58	5.00	3.00	3.00	28.72
58	5.00	3.00	3.00	28.72
60	3.00	2.00	2.00	29.14
100	4.00	3.00	3.00	42.68
60	2.00	2.00	2.00	29.14
100	4.00	3.00	3.00	42.68
60	3.00	3.00	3.00	29.14
100	4.00	3.00	3.00	42.68
60	2.00	2.00	2.00	29.14
100	4.00	3.00	2.00	42.68
60	3.00	3.00	3.00	29.14
100	4.00	3.00	3.00	42.68
60	2.00	2.00	2.00	29.14
100	4.00	3.00	3.00	42.68
48	3.00	5.00	5.00	24.89
48	3.00	5.00	5.00	24.89
48	3.00	5.00	5.00	24.89
48	3.00	3.00	3.00	24.89
48	3.00	3.00	3.00	24.89
70	3.00	1.00	1.00	32.03
70	2.00	1.00	1.00	32.03
70	2.00	1.00	1.00	32.03
70	2.00	2.00	2.00	32.03
72	2.00	1.00	1.00	35.00
58	3.00	3.00	3.00	28.72
57	2.00	3.00	3.00	27.89
60	3.00	3.00	3.00	29.55
53	3.00	3.00	3.00	27.11
58	3.00	3.00	3.00	28.72
57	2.00	3.00	3.00	27.89
60	3.00	3.00	3.00	29.55
53	3.00	3.00	3.00	27.11
58	3.00	3.00	3.00	28.72
57	2.00	3.00	3.00	27.89
60	3.00	3.00	3.00	29.55
53	3.00	3.00	3.00	27.11
58	3.00	3.00	3.00	28.72
57	2.00	3.00	3.00	27.89
60	3.00	3.00	3.00	29.55
53	3.00	3.00	3.00	27.11
48	3.00	3.00	3.00	24.89
58	3.00	3.00	3.00	28.72
57	1.00	3.00	3.00	27.89
60	3.00	3.00	3.00	29.55
53	3.00	3.00	3.00	27.11
48	3.00	3.00	3.00	24.89
58	3.00	3.00	3.00	28.72
53	4.00	3.00	3.00	27.11
53	4.00	3.00	3.00	27.11
53	4.00	3.00	3.00	27.11

53	4.00	3.00	3.00	27.11
53	3.00	3.00	3.00	27.11
53	3.00	3.00	3.00	27.11
72	4.00	3.00	3.00	30.49
80	3.00	3.00	3.00	35.94
72	4.00	3.00	3.00	30.49
80	3.00	3.00	3.00	35.94
72	4.00	3.00	3.00	30.49
80	3.00	3.00	3.00	35.94
72	4.00	3.00	3.00	30.49
80	3.00	3.00	3.00	35.94
72	4.00	3.00	3.00	30.49
70	3.00	3.00	3.00	32.03
67	4.00	2.00	2.00	29.72
67	4.00	2.00	2.00	29.72
67	4.00	3.00	3.00	29.72
67	4.00	2.00	2.00	29.72
67	4.00	3.00	3.00	29.72
64	4.00	3.00	3.00	27.55
64	4.00	3.00	3.00	27.55
64	4.00	3.00	3.00	27.55
64	4.00	3.00	3.00	27.55
64	4.00	3.00	3.00	27.55
64	4.00	3.00	3.00	27.55
62	3.00	3.00	3.00	26.81
62	3.00	3.00	3.00	26.81
55	3.00	3.00	3.00	27.06
62	3.00	3.00	3.00	26.81
62	3.00	3.00	3.00	26.81
56	3.00	3.00	3.00	26.64
62	4.00	3.00	3.00	26.81
56	3.00	3.00	3.00	26.64
60	4.00	3.00	3.00	31.56
72	2.00	3.00	3.00	30.49
72	2.00	3.00	3.00	30.49
72	2.00	3.00	3.00	30.49
72	2.00	3.00	3.00	32.42
72	2.00	3.00	3.00	32.42
67	3.00	5.00	5.00	29.72
67	3.00	5.00	5.00	29.72
67	3.00	5.00	5.00	29.72
67	3.00	3.00	3.00	29.72
67	3.00	3.00	3.00	29.72
52	2.00	3.00	4.00	26.67
55	4.00	3.00	3.00	27.06
110	4.00	3.00	3.00	44.81
72	4.00	3.00	3.00	35.00
52	2.00	5.00	5.00	26.67
55	4.00	3.00	3.00	27.06
57	2.00	2.00	2.00	27.89
110	4.00	3.00	3.00	44.81
72	4.00	3.00	3.00	35.00

56	4.00	3.00	3.00	26.64
52	2.00	3.00	3.00	26.67
55	4.00	3.00	3.00	27.06
57	3.00	1.00	1.00	27.89
100	4.00	3.00	3.00	42.68
72	4.00	3.00	3.00	35.00
52	2.00	3.00	4.00	26.67
55	4.00	3.00	3.00	27.06
57	3.00	1.00	1.00	27.89
100	4.00	3.00	3.00	42.68
72	3.00	3.00	3.00	35.00
56	4.00	2.00	2.00	26.64
52	2.00	3.00	3.00	26.67
55	4.00	3.00	3.00	27.06
57	2.00	1.00	1.00	27.89
100	4.00	3.00	3.00	42.68
72	3.00	3.00	3.00	35.00
56	4.00	3.00	3.00	26.64
52	2.00	3.00	3.00	26.67
55	4.00	3.00	3.00	27.06
57	3.00	1.00	1.00	27.89
100	4.00	3.00	3.00	42.68
